# EpiDBase: a manually curated database for small molecule modulators of epigenetic landscape

**DOI:** 10.1093/database/bav013

**Published:** 2015-03-16

**Authors:** Saurabh Loharch, Isha Bhutani, Kamal Jain, Pawan Gupta, Debendra K. Sahoo, Raman Parkesh

**Affiliations:** Department of Advanced Protein Science, Institute of Microbial Technology, Chandigarh 160036, India

## Abstract

We have developed EpiDBase (www.epidbase.org), an interactive database of small molecule ligands of epigenetic protein families by bringing together experimental, structural and chemoinformatic data in one place. Currently, EpiDBase encompasses 5784 unique ligands (11 422 entries) of various epigenetic markers such as writers, erasers and readers. The EpiDBase includes experimental IC_50_ values, ligand molecular weight, hydrogen bond donor and acceptor count, XlogP, number of rotatable bonds, number of aromatic rings, InChIKey, two-dimensional and three-dimensional (3D) chemical structures. A catalog of all epidbase ligands based on the molecular weight is also provided. A structure editor is provided for 3D visualization of ligands. EpiDBase is integrated with tools like text search, disease-specific search, advanced search, substructure, and similarity analysis. Advanced analysis can be performed using substructure and OpenBabel-based chemical similarity fingerprints. The EpiDBase is curated to identify unique molecular scaffolds. Initially, molecules were selected by removing peptides, macrocycles and other complex structures and then processed for conformational sampling by generating 3D conformers. Subsequent filtering through Zinc Is Not Commercial (ZINC: a free database of commercially available compounds for virtual screening) and Lilly MedChem regular rules retained many distinctive drug-like molecules. These molecules were then analyzed for physicochemical properties using OpenBabel descriptors and clustered using various methods such as hierarchical clustering, binning partition and multidimensional scaling. EpiDBase provides comprehensive resources for further design, development and refinement of small molecule modulators of epigenetic markers.

**Database URL:** www.epidbase.org

## Introduction

Epigenetics is the study of alteration in cellular phenotype that does not affect the DNA sequence, e.g. DNA methylation, histone and covalent modifications of proteins. Epigenetic changes are modulated by different markers such as writers, erasers and readers (1–4). Epigenetic changes are dynamic in nature and active throughout the lifespan of a cell. As a consequence, epigenetics is greatly influenced by environmental factors such as stress, lifestyle, food habits and exposure to environmental elements (5, 6). There are great prospects in modifying the epigenetic states to treat illness, e.g. targeting epigenetic proteins with small molecule ligands. The recent approval of two classes of epigenetic-targeting drugs, DNA methyl transferase inhibitors (DNMTs) and histone deacetylase inhibitors (HDACs), indicates that epigenetic proteins are druggable (6–10). There are issues of non-specificity of these drugs toward respective DNMTs and HDACs. Because epigenetic changes influence the overall gene expression, the properties of the ligands as toxicity and non-specificity of inhibitors should be investigated methodically. There is a necessity to develop effective small molecule tools to understand the underlying biology of epigenetic codes and a considerable research effort is now being directed, both in academia and industry toward achieving this goal.

Despite the importance of epigenetic proteins in therapeutics, there is a lack of interactive and curated databases. Few databases such as NCBI Epigenomics, HEMD, ChEpimod, ChEMBL and ChromoHub have been reported recently to provide access to epigenetic proteins and their phylogenetic associations along with a list of inhibitors (11–16). To the best of our knowledge, there is a paucity of resources that bring together epigenetic modulators and related data to permit comprehensive analysis for drug discovery. An epigenetic database that analyzes and presents unique set of drug like ligands, shows their absorption, distribution, metabolism, excretion and toxicity (ADMET) parameters, allows diverse text or structure search and supports chemoinformatic analysis would be highly beneficial for drug discovery scientists. This motivated us to design EpiDBase as an interactive interface to view, explore, search and analyze the small molecule modulators targeting various epigenetic protein families. EpiDBase includes text search, chemical fingerprint search, two-dimensional (2D) structural editor and three-dimensional (3D) structural viewer, thus providing powerful tools to browse, search and analyze various small molecule modulators of epigenetic protein families. In addition, EpiDBase is curated to identify unique molecular scaffolds. The database provides the toxicological profile of around 5784 compounds, of which 3737 passed the toxicology filter such as ZINC (17) and Lilly MedChem rules (18). These 5784 molecules were also analyzed for their conformation sampling by generating 3D conformers. Cluster analysis was also performed on these molecules. We believe EpiDBase provides important drug discovery resources for the larger scientific community working in cancer, neurodegenerative disorders and other related areas involving epigenetic protein families.

## Materials and methods

### Data collection

The relevant articles (1999–2014) were collected from PubMed, Web of Science and Google Scholar using keyword searches such as ‘small molecule targeting epigenetic’, ‘small molecule inhibitors of epigenetic protein’, ‘small molecule inhibitor AND protein name’, ‘small molecule modulator AND/OR protein name’, and ‘Targeting epigenetic protein family with small molecules’. Total number of hits found using the above search terms were 1276 (PubMed), 3089 (Web of Science) and 305 064 (Google scholar), respectively. Google scholar was searched especially for those protein targets wherever zero or few hits were found in PubMed and Web of Science. A total of 1268 relevant articles (with 588 common to all of the above 3 databases) were further processed to extract information about ligands. The articles obtained were explored and the data pertaining to epigenetic proteins–ligand complexes, such as protein structure, ligand structure, binding constant, IC_50_, were collected. In many cases, the cited reference in each article was checked to find detailed information on proteins and their modulators. ChemBioDraw Ultra (v11.0) was used as the drawing tool to create the structures of these ligands and the structures were saved in Structure Data File (SDF) format in the database. To facilitate computer readability, the chemical structure of ligand molecules illustrated in the articles using scaffold and abbreviated R-group substituents were redrawn as complete chemical structures and incorporated into the database.

### Database description

The database was built using Apache HTTP (web server) and MySQL (database server) as the relational database management system to store information (see online supplementary material for supplementary file S1). The graphical layout of EpiDBase with various searches, browse and analysis tools are shown in supplementary file S2 (see online supplementary material).

### Data visualization

**Figure 1.**
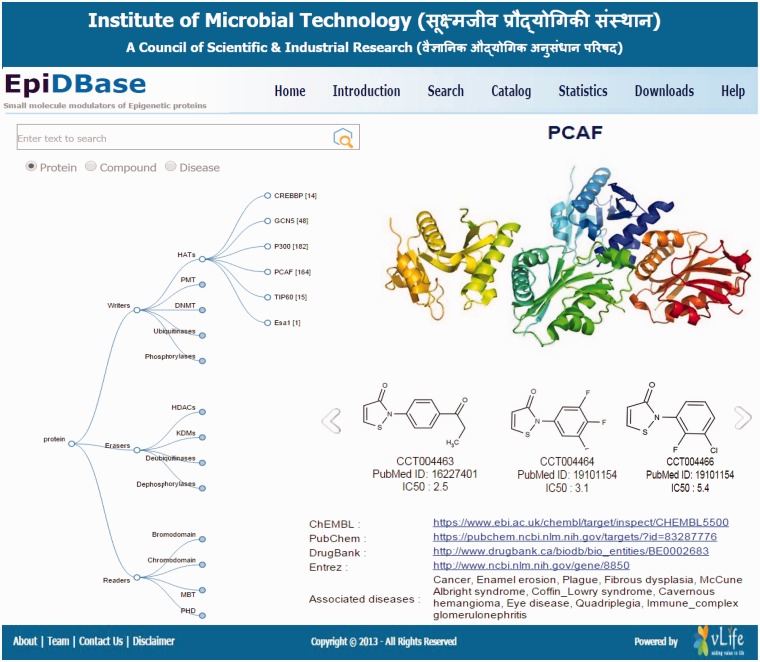
Screenshots of EpiDBase homepage depicting the interactive tree relation between protein class and ligand molecules.

The data visualization was developed using D3.js JavaScript library (http://d3js.org/), allowing for creation of plug-in free, and browser-based interactive design (Figure 1).

### Descriptor calculation

The VLifeMDS command line version (www.vlifesciences.com) was used for calculating 2D compound descriptor such as acceptor count, donor count, rotatable bonds, aromatic rings and XlogP (19). For hydrogen bond donor (HBD) and hydrogen bond acceptor (HBA) count in the molecules, hydrogen atoms were classified as reported by Wang *et al.* (20). For quantitative structure activity relationship studies (QSAR) and to characterize the hydrophilic nature of modulators, logP values were calculated using the program XlogP3 (21, 22). The number of rotatable bonds and aromatic rings were described giving information about the conformation sampling of the molecules, which are important parameters for structure and ligand-based drug discovery. The aromatic ring count descriptor illustrates the propensity of the molecules for π–π interactions with the binding site residues.

### Substructure search

VLifeMDS command line version was integrated into the web application for performing substructure search.

### 2D structure editor for structure search

**Figure 2.**
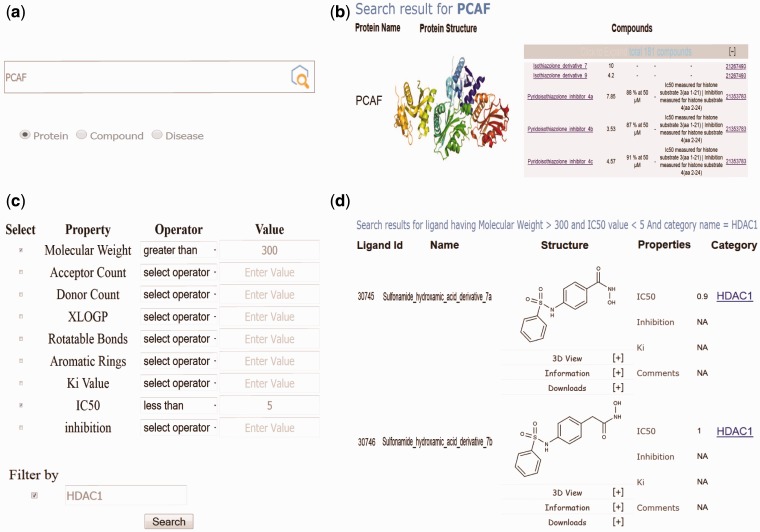
Screenshots of EpiDBase showing (a) protein and ligand-based text search; (b) results of protein-based text search; (c) advanced search dialog box for ligand search based on advanced properties such as MW, IC_50_, hydrogen bond donor, acceptor count, *K_i_* and so on; (d) results of advanced search based on MW cutoff of >300 and IC_50_ <5 and filtered by protein HDAC1.

VLife2Draw (23) was employed as an editor and viewer to search 2D molecular structures in EpiDBase (Figure 2). VLife2Draw is a Java-based applet and was embedded in the webpage browser with a suitable Java plug-in. VLife2Draw provides a powerful function to draw and edit molecular structures. In addition, it is possible to draw stereoisomers using stereo up and down chemical bonds. Users can also import and export chemical structures of modulators in mol and SDF formats. Templates for drawing 3- to 8-membered ring structures were also provided.

**Figure 3.**
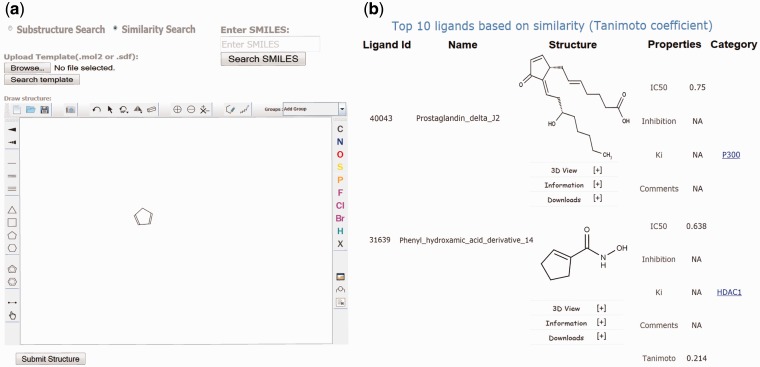
Screenshots of the EpiDBase webpage showing (a) substructure and similarity search and (b) results of similarity search.

### Fingerprint-based similarity analysis

EpiDBase uses OpenBabel (http://openbabel.org/) fingerprint search for similarity analysis (24).

### 3D structure viewing

For 3D visualization, we used JMol molecule viewer [an open-source Java viewer for chemical structures in three dimension (http://www.jmol.org/)]. The structures were represented by standard ball-and-stick model using Corey-Pauling-Koltun (CPK) coloring scheme of atoms (carbon, dark gray; hydrogen, light gray; oxygen, red; nitrogen, green) (25, 26).

### Computational ADMET/toxicology analysis

For ADMET analysis, the library of 5784 compounds was manually curated by removing large structures such as peptides, macrocycles, larger molecular weight compounds and salts. The remaining 5401 compounds were then assigned correct protonation state using Pybel (27). Subsequently, the normalization was performed using ChemAxon Standraizer (JChem 6.0.1, http://www.chemaxon.com). These molecules were further filtered using ZINC (for detailed description, see online supplementary material for supplementary file S3) and Lilly MedChem regular rules with a 100-demerit cutoff. LogP was calculated using XlogP3 program. On the basis of these filters, the compounds were either accepted or rejected. ADMET analysis was performed using FAF-Drugs2 (28), which is python-based command line utility program. FAF-Drugs2 uses OpenBabel toolkit to calculate various physicochemical descriptors and filters toxic, unstable molecules and/or functional groups.

### Conformer generation

Three-dimensional conformations of ligands were generated using opensource free software RDKit (http://www.rdkit.org/). RDKit has been shown to perform better in generating biologically relevant low energy conformes for small molecules among various open source tools (29). It employs distance geometry methods to generate 3D conformations of small molecules. The distance geometry approach involves the creation of a matrix consisting of the lower and upper bound of all pairwise distances of a molecule. In effect, this matrix encompasses the whole structural space covered by the molecule. The multiple conformation generation process involves the creation of a random distance matrix, which satisfies the upper and lower bound of the distance matrix of a molecule. We generated about 50 conformers for each molecule using root-mean square distance (rmsd) cutoff of 0.5. The conformers generated for each molecule were further optimized by using Universal Force Field (UFF) (30).

### Clustering analysis

Clustering analysis of the database molecules was performed using the ChemMine software (http://chemmine.ucr.edu/) (31). ChemMine provides three clustering algorithms: hierarchical clustering, multidimensional scaling (MDS) and binning clustering. Hierarchial clustering provides tree-based visualization of clustering data, while MDS allows scatter plot visuals of clusters. Binning clustering classifies the compounds into similarity groups, based on the user-specified Tanimoto coefficient. We used binning clustering algorithm with a similarity cutoff (Tanimoto coefficient) of 0.6 for the analysis.

## Results and discussion

### Database contents

EpiDBase brings together diverse ligands that target various epigenetic protein families. EpiDBase consists of 11 422 entries comprising of 5784 unique ligands associated with 220 proteins (see online supplementary material for supplementary file S4). The database provides various properties such as its IC_50_ value, Ki value, % inhibition, 2D and 3D structures, molecular weight, acceptor count, donor count, XlogP value, rotatable bonds, aromatic rings, InChIKey, links to other associated proteins and PubMed ID for each ligand. The proteins are also associated with different properties such as protein name, its structure, a list of related ligands, associated diseases and links to ChEMBL, PubChem, DrugBank and Entrez gene. The ligands in EpiDBase are illustrated as images and both their 2D and 3D structures can be downloaded. We have also provided the 3D viewer and each ligand is represented and visualized in various 3D representations such as CPK, ball and stick and wireframe. It is possible to render 3D surface of each molecule as dots, Van der Waals surface, solvent accessible, electrostatic and molecular representations. The download menu option in the database provides access to all database molecules for off-line analysis. Most of the database ligands show class specificity to the enzymes (e.g. PBHA, ACY1215, APHA, apicidin and trichistatin A are specific to histone deacetylases; curcumin, CoASH, AMA1 and AMA2 are specific to histone acetyltransferases). However, few modulators show different roles, e.g. bisaprasin inhibits both DNMT1 and HDACs, and GSK926 targets DNMT1, DNMT3A, DNMT3B, CARM1, DOT1L, EHMT2 and KMT2A. For a compound, apart from the protein mentioned in the category, other associated proteins can also be retrieved under the ‘Information category’ menu in EpiDBase.

### Organization and data retrieval of EpiDBase

**Figure 4.**
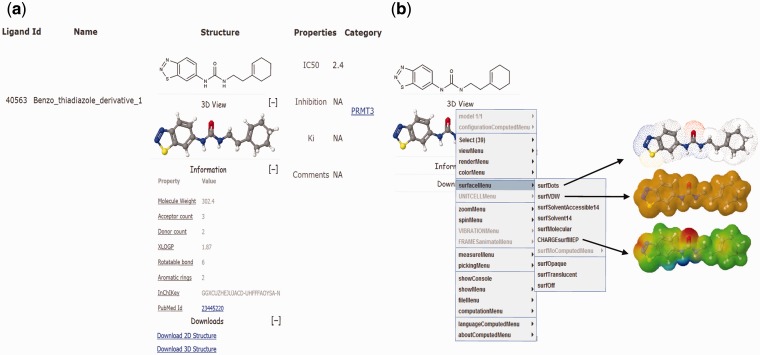
EpiDBase ligand screenshot showing (a) 2D and 3D structural view of the ligand showing advanced property, reference, comments and their download links and (b) 3D surface view depicted as dots, van der Waals and charge.

EpiDBase is accessible via a user-friendly Graphical User Interface (GUI) at the web address www.epidbase.org. We have organized the database in such a way that it would enable the effective illustration of the relation between protein main classes right down to their small molecule modulators. Interactive tree-based visualization (Figure 1) of epigenetic protein families was embedded to support and facilitate straightforward browsing of the database. The database allows name-based search for both protein and ligand (Figure 2), ligand substructure search and ligand-based fingerprint search (Figure 3). In addition, dialog box is also included to facilitate easy and quick location of ligands and proteins. The small molecules can be searched by their common names. We have also provided InChIKey for defining the molecules (32). Figure 4a shows a screenshot of the ligand with its InChIKey. InChIKey offers several advantages over IUPAC names, such as a short and a unique ID which can be easily searched. For a small molecule, this search would find all the epigenetic proteins where the molecule acts as modulator together with related experimental data. Additionally for each compound, 2D and 3D structures along with all other calculated properties pop up in a new window. Both 2D and 3D structures can be downloaded. For 3D representation of EpiDBase molecule, 2D structure is first corrected for errors and appropriate number of hydrogen atoms is then added. Energy minimization of 3D structure was performed using MMFF force field with a gradient convergence of 1.0 kcal/(molÅ). Energy minimized structures were further processed taking into account hydrogen atom optimization, correct geometry and stored as Mol2 files in the database. In cases where X-ray structure of the protein–ligand complex is available, the Protein Data Bank (PDB) code is provided to facilitate structural analysis of the complex. A standard ball-and-stick model is used to display the ligands (Figure 4a). Additionally, other structural displays such as CPK spacefill, sticks and wireframe representation are possible by right clicking (using mouse or touchpad) and then selecting the renderMenu from the JMol dialog box. Molecular surfaces such as Van der Waals, solvent accessible, dots and charge are included (Figure 4b). We have also provided a catalog of all EpiDBase ligands which is accessible through the homepage. The catalog contains structures of all molecules sorted according to their molecular weight and various properties (e.g. HBD and HBA count, XlogP, number of rotatable bonds, number of aromatic rings and InChIKey values).

### Online tools for structure and similarity search

To facilitate structure and similarity search analysis, many interactive web-based tools are implanted in the database. The description of these tools is shown below:

#### Advanced search

EpiDBase offers users the advanced search option to explore the compounds based on various common physicochemical properties such as molecular weight, XlogP and number of rotatable bonds (Figure 2). Advanced search can be carried out either using a specific property or combination of different properties, e.g. a user can filter the search results using MW alone or MW and XlogP. Users can use complex logic to help refine search by using various logical operators such as >, <, =, AND/OR and range that provide further control over the search results. Users can further limit their search by applying filter based on protein families.

#### Substructure search

EpiDBase has powerful structural editor (VLife2Draw) embedded to help in substructure-based search analysis. VLife2Draw supports both partial and complete structural analysis through Vlife2Draw applet. Users can submit a partial or complete structure for analysis. The applet converts and stores the submitted structure into a temporary SDF format on the host server. This SDF is then used to search the EpiDBase for molecular structure similarity by a python script provided in the VLifeMDS scripting platform. The output of search is presented as a list of molecules fulfilling the substructure similarity criteria as specified by the user. In brief, the algorithm followed in substructure search involves the following steps: (i) user-defined input as partial or complete structure; (ii) molecular template generation using atoms, bonds and rings; (iii) atom template match; (iv) establishing molecular connectivity using graphy theory and (v) hitlist generation by matching atom template and graph connectivity of the query molecule with the database molecules. A complete description of the process is detailed in Mehta *et al*. (19).

#### Similarity search

EpiDBase uses ‘babel’ program of OpenBabel software (http://openbabel.org) for similarity and substructure search analysis. The similarity/substructure search algorithm uses molecular fingerprint method (24). A fingerprint is a series of binary (1/0) bits that are arranged in sequence. Molecular fingerprints display molecular structures as computer-coded binary bits where each bit’s position correspond to certain information such as presence or absence of an atom, type of ring, element count and a substructure. In EpiDBase, we have precomputed and stored the binary fingerprint for all database molecules. During similarity search, a binary fingerprint is automatically generated for a ‘user-defined query’ and matched with the fingerprints of the database molecules. The output is generated as a list, displaying structures with similarity score quantified in terms of Tanimoto coefficient. The value of Tanimoto coefficient is between 1.0 and 0, with 1.0 being the most similar structures and 0 being completely different structures. The output of similarity search is arranged in descending order with the top hits representing the most similar structures relative to the query molecule (Figure 3). Ligand similarity and substructure search of EpiDBase are quite versatile as it has the capability to identify as well as rank the structures based on the query.

### Significance of EpiDBase

EpiDBase is a complete repository which provides easy access to the biologically relevant ligands of the epigenetic proteins. The database contains molecules for which the experimental data or the binding to epigenetic proteins has been reported in the literature. The corresponding references have also been included for the user to cross-validate if necessary. The PubChem Bioassay data were not included to avoid duplication and problems related to high-throughput screening validation. Currently, EpiDBase holds 5784 unique ligands and 220 epigenetic proteins. Previously, few other databases related to epigenetics have also been reported such as NCBI Epigenomics, ChEpimod, ChromoHub, HEMD and ChEMBL. NCBI Epigenomics provides the information related to genome-wide maps of the DNA and histone modifications with more emphasis on the whole-genome epigenetic datasets. The focus of ChromoHub is mainly on the genes and proteins involved in chromatin-mediated signaling. ChEpimod is mainly a structural database and provides information about the inhibitors which target reader protein family. To demonstrate the uniqueness of EpiDBase, we compared it with related databases, HEMD (14) and ChEMBL (16) using ChemDiff tool (http://www.ggasoftware.com/opensource/indigo/chemdiff). In ChEMBL, the majority of the epigenetic ligands are extracted from PubChem bioassay. By browsing the epigenetic targets from ChEMBL, we found that for a reader protein ‘Chrombox protein 1’ (ChEMBL1741193) all the molecules shown are from PubChem bioassay. Furthermore, there are only 109 unique molecules out of 55 933 molecules in case of the protein ‘Bromodomain adjacent to zinc finger domain protein 2B’ (ChEMBL1741220). In comparison with ChEMBL (127 740 ligands), we found that EpiDBase (5784 ligands) has ∼88% unique molecules (with 264 molecules common; see online supplementary material for supplementary file S5). Analysis of the HEMD database (4317 ligands) revealed that in addition to small molecules, proteins and inorganic molecules are also part of the database. After omitting these ligands, EpiDBase has ∼81% unique molecules (with 696 molecules common; see online supplementary material for supplementary file S5) in comparison with HEMD (4054 ligands). In EpiDBase, we have manually collected the data from each article and also from the cited references. We were able to cover a large number of small molecule modulators for various epigenetic protein families. Care was taken to avoid data duplication. Molecules illustrated in the articles using scaffold and abbreviated R-group substituents were expanded to complete chemical structures before adding to the EpiDBase. We believe that our manual curation could be the reason why EpiDBase shows a very small overlap with HEMD and ChEMBL. This clearly indicates the novelty of EpiDBase and its potential utility in drug discovery or finding small molecule modulators for targeting various epigenetic proteins.

### Computational ADMET/toxicity prediction

The reason for failure of most of the drugs during clinical trials is due to their toxicity and unfavorable ADMET properties (33, 34). Thus, it is important in the early drug discovery stages to predict and optimize ADMET and toxicological properties of drug molecules. We processed about 5401 small molecules using ZINC and Lilly MedChem regular rules (for ZINC parameter, see online supplementary material for supplementary file S3). Of these 5401 molecules, 1664 molecules were rejected by the filter rules. Distribution of various physicochemical descriptors such as HBA, HBD, polar surface area and logP of accepted 3737 molecules is shown in supplementary file S6 (see online supplementary material). We believe that these 3737 molecules can be further used for virtual screening and docking experiments to find additional ligands for various epigenetic protein families.

### 3D conformation

Conformational models are important for pharmacophore modeling, rigid docking, shape-based screening, 3D-QSAR and virtual screening. The epigenetic drug discovery will be greatly facilitated by the accurate representation of low energy conformers of the various lead molecules. To facilitate this, we generated a multiconformer database of 5784 compounds using UFF. The conformers were generated using distance geometry approach as implemented in open source toolkit RDKit. For each compound, 50 conformers were generated specifying the rmsd cutoff of 0.5. For some compounds, the number of conformers generated were less than the specified as a result of rmsd criteria. We believe that this multi-conformer database with 284 158 structures will be very useful as it provides the structural information about the conformational states of all the compounds, which is vital for *in silico* drug designing.

### Clustering analysis

**Figure 5.**
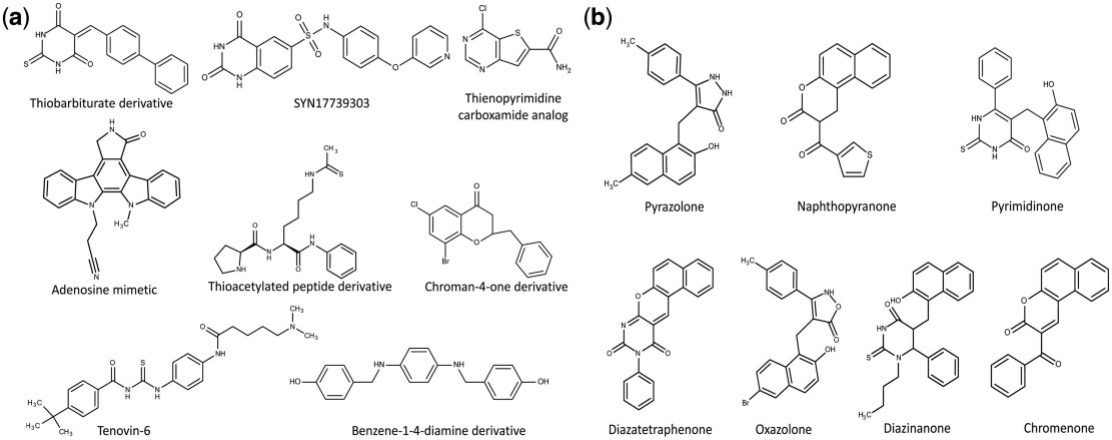
The chemical structures of the EpiDBase molecules with some representative examples of various bin cluster: (a) molecular scaffold representing smallest bin cluster (only 1 structure); and (b) molecular scaffold representing the largest bin cluster size (83 structures).

Clustering is a powerful tool employed in correlating compound features with biological activity. Clustering analysis provides information about diversity of molecules and thus is an important tool for lead optimization and drug discovery (35, 36). Clustering methodology involves the generation of appropriate dataset, selection of correct similarity measurement, clustering of the dataset, analysis of the data and finally visualization of the clustered data (37). We used ChemMine tool to perform clustering analysis. ChemMine provides three important clustering methods such as hierarchical, MDS and binning clustering. To understand the structural diversity and the chemical space occupied by small molecules targeting various epigenetic protein families, we performed binning clustering using a similarity cutoff (Tanimoto coefficient) of 0.6. By using bin-based partition, it is possible to classify compounds into various similarity groups. We used a representative example of SIRT2. Currently, in EpiDBase, there are around 608 molecules which are reported as SIRT2 ligands. We used binning clustering to generate compound similarity groups of SIRT2 ligands based on the Tanimoto coefficient of 0.6. Binning partition of the SIRT2 active molecules resulted in the 108 similarity groups or cluster with varied number of molecules (see online supplementary material for supplementary file S7). The largest bin cluster included 83 small molecules with different chemical scaffolds (Figure 5). The main class of compounds in this largest bin cluster was represented by pyrazolone (21 structures), pyrimidinone (30 structures), naphthpyranone (20 molecules), diazatetaraphenone (4 structures), diazzinaneone (3 structures), oxazolone (3 structures), naphthalene-2-carbonitrile (1 structure) and chromenone (1 structure). Additionally, 65 bin clusters were represented by a single molecule (Figure 5). We believe that clusters which are represented by one or two molecular scaffolds can be populated by using medicinal chemistry and SAR studies. This will lead to the rational design of the small molecules targeting SIRT2.

### Fragment library generation

We used the data in EpiDBase to generate a fragment database of SIRT2 modulators using Retrosynthetic Combinatorial Analysis Procedure (38) as implemented in MedChem Studio^™^ (Simulation Plus, Inc.). In this retrosynthesis analysis, the molecules are cleaved along various bonds using the chemical rules and the resulting fragments are collected. The fragments were analyzed for their occurrence frequency and clustered. The assembled fragments form the basis for designing target motif and fragment libraries using organic synthetic procedures (see online supplementary material for supplementary file S8 for SIRT2-specific fragment library). We believe that these fragment libraries will play an important role in fragment-based design strategy for finding sirtuin modulators. A fragment library can be developed for any epigenetic protein family using the data contained in the EpiDBase.

## Conclusion and future prospects

We have developed EpiDBase (www.epidbase.org), a chemoinformatics platform to facilitate interactive exploring of epigenetic proteins and their modulators. The database comprises diverse epigenetic protein families, their reported ligands, experimental IC_50_ values, structural data, toxicological and chemoinformatic information. The database can be browsed using protein family names, disease area, ligand names, ligand structures, substructures and fingerprint-based chemical similarity search. In its current version, EpiDBase would facilitate SAR studies, statistical analysis and fragment-based drug design. We aim to introduce additional tools, which can allow virtual screening (3D shape) and molecular docking studies in near future, to assist epigenetic drug discovery. In general, this publicly available database would be beneficial for the design and discovery of modulators to influence epigenetic states of various diseases such as cancer, diabetes, neurodegenerative disorders and cardiovascular disorders. The main aim of the database is to provide computational and medicinal chemistry tools for research community who are interested in medicinal chemistry, biology and biochemistry of epigenetic protein families. We believe that the set of tools provided here will invite scientists from diverse background to initiate drug design and development in the field of epigenetics. We plan to update and further improve EpiDBase annually. In future versions, we would include blast tool, protein alignment tool, fragment generation capability, principal component analysis, R-group decomposition and 3D structure comparison and automatic docking protocol.

## Supplementary Data

Supplementary data are available at *Database* Online.

## Funding

This work was supported by the Institute of Microbial Technology (grant Infra 62 to R.P.), Institute of Microbial Technology Research Internship grant to I.B. and CSIR JRF to S.L. Funding for open access charge: Intramural research funds of Institute of Microbial Technology, Chandigarh.

*Conflict of interest*. None declared.

## Supplementary Material

Supplementary Data
