# Supplementary material for: EpiDBase: a manually curated database for small molecule modulators of epigenetic landscape
Source: Database (Oxford). 2015 Mar 16;2015:bav013. doi: 10.1093/database/bav013 (PMC4360624; doi:10.1093/database/bav013)
Supplement: Supplementary Data [file supp_2015_bav013_index.html]

EpiDBase: a manually curated database for small molecule modulators of epigenetic landscape — Supplementary Data 

# EpiDBase: a manually curated database for small molecule modulators of epigenetic landscape

## Supplementary Data

files

**Files in this Data Supplement:**

- Supplementary Data - zip file
