# Supplementary material for: EpiDBase: a manually curated database for small molecule modulators of epigenetic landscape
Source: Database (Oxford). 2015 Mar 16;2015:bav013. doi: 10.1093/database/bav013 (PMC4360624; doi:10.1093/database/bav013)
Supplement: Supplementary Data [file supp_bav013_suppl_data.zip › File_S4.docx]

**File S4: EpiDBase Protein and Ligand properties**

| **Feature** | **Protein** | **Ligand** |
| --- | --- | --- |
| Total number | 222 | 5784 |
| Properties | Protein name | Ligand name |
|  | Protein structure | Ligand structure: 2D and 3D |
|  | List of related compounds with  IC50 value, % inhibition and PubMed ID | Download option for 2D and 3D structure |
|  | Link to ChEMBL | IC50 value |
|  | Link to PubChem | Ki value |
|  | Link to DrugBank | % inhibition |
|  | Link to Entrez gene | Molecular weight |
|  | Associated diseases | Acceptor count |
|  |  | Donor count |
|  |  | XLOGP |
|  |  | Rotatable bonds |
|  |  | Aromatic rings |
|  |  | InChIkey |
|  |  | Associated protein |
|  |  | Link to other associated proteins |
|  |  | Link to PubMed ID |
