# Supplementary material for: EpiDBase: a manually curated database for small molecule modulators of epigenetic landscape
Source: Database (Oxford). 2015 Mar 16;2015:bav013. doi: 10.1093/database/bav013 (PMC4360624; doi:10.1093/database/bav013)
Supplement: Supplementary Data [file supp_bav013_suppl_data.zip › File_S7.pdf]

Bin cluster size of 1

1	2	3	4	5
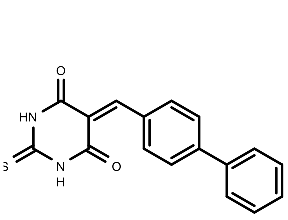	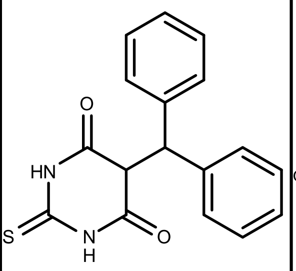	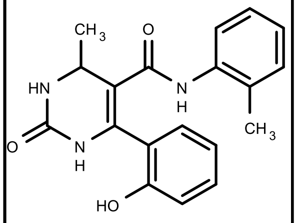	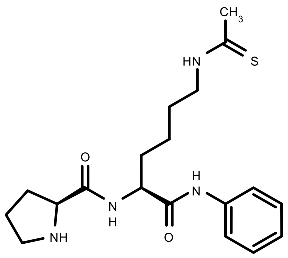	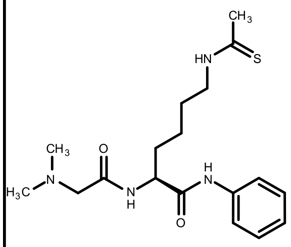
Thiobarbiturates_derivative1_25.mol	Thiobarbiturates_derivative1_11.mol	Thiobarbiturates_derivative1 10.mol	Thioacetylated_peptide_derivative 22.mol	Thioacetylated_peptide_derivative 20.mol
**6**	**7**	**8**	**9**	**10**
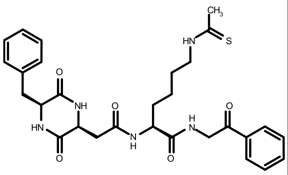	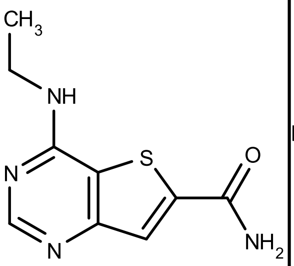	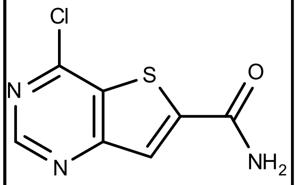	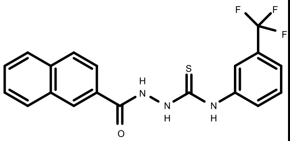	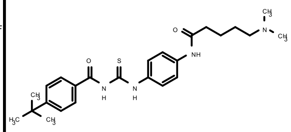
Thioacetylated_peptide_derivative 18.mol	71654838 Thieno_pyrimidine_carboxamide analog	71654770 Thieno_pyrimidine_carboxamide ana	Tenovin_derivative1_15.mol	Tenovin_6.mol
**11**	**12**	**13**	**14**	**15**
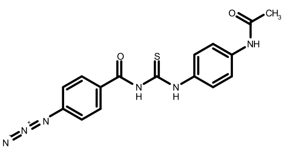	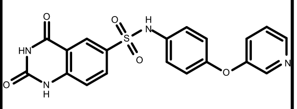	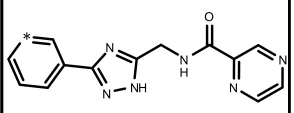	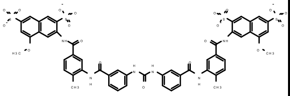	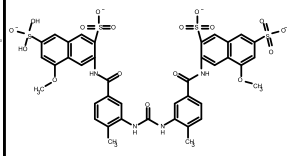
Tenovin_4.mol	Untitled Document-5 SYN17739303.mol	Untitled Document-16 SYN10366754.mol	Suramin_analogue_NF770.mol	Suramin_analogue_NF769.mol
**16**	**17**	**18**	**19**	**20**
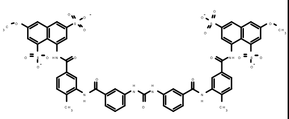	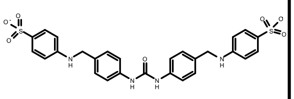	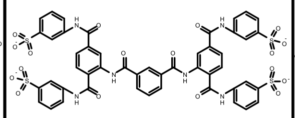	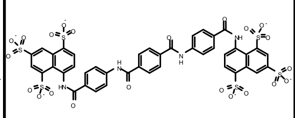	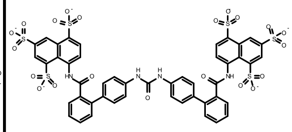
Suramin_analogue_NF763.mol	Suramin_analogue_NF669.mol	Suramin_analogue_NF451.mol	Suramin_analogue_NF343.mol	Suramin_analogue_NF136.mol

Bin cluster size of 1

21	22	23	24	25
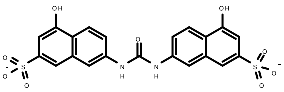	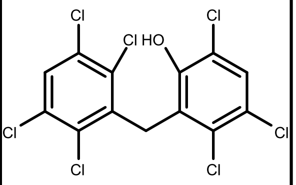	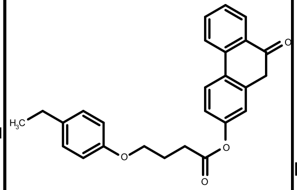	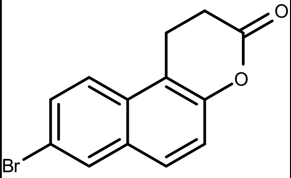	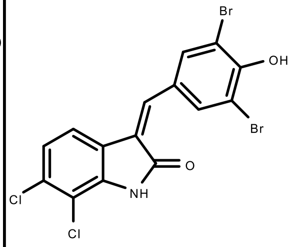
Suramin_analogue_AMI_1.mol	Splitomicin_related_21.mol	Splitomicin_related_20.mol	splitomicin_derv_16.mol	Oxindole.mol
**26**	**27**	**28**	**29**	**30**
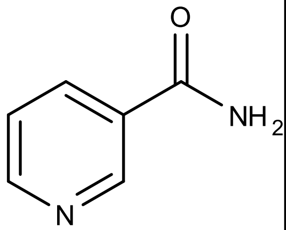	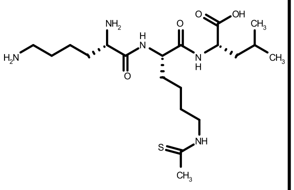	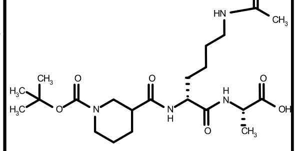	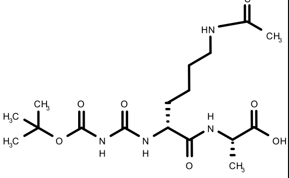	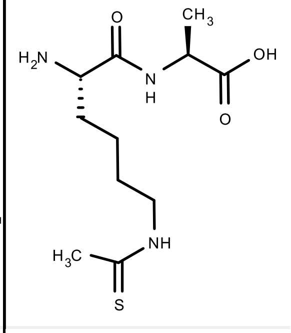
Nicotinamide.mol	HKK(thioaceyl)LM.mol KK_thioaceyl L.mol	K_thioaceyl_A_derv_6.mol K_thioaceyl A der	Ac_PK(thioaceyl)A.mol K_thioaceyl A derv 1.mo	AK(thioaceyl)A.mol K_thioaceyl A.mol
**31**	**32**	**33**	**34**	**35**
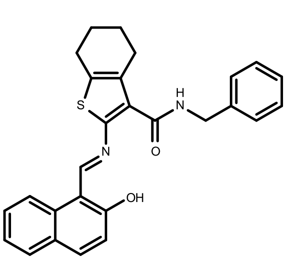	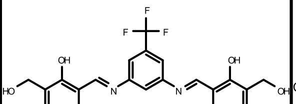	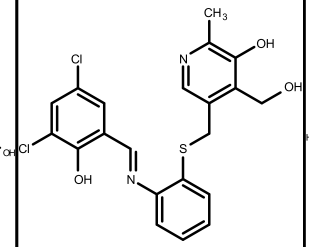	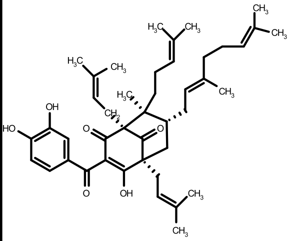	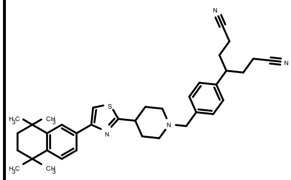
JGB1741.mol	2_aminosuberic_acid_derv_2_rac.mol hydroxymet	hydroxybenzylideneamino_derv.mol	Guttiferone G.mol	hydroxymethyl_phenol_derv_1.mol etrahydron
**36**	**37**	**38**	**39**	**40**
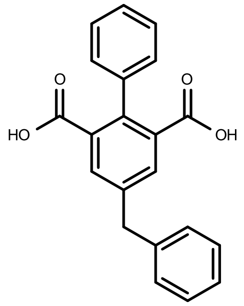	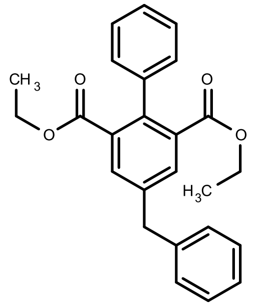	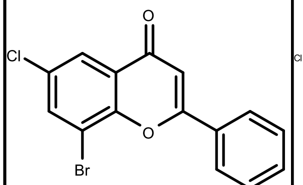	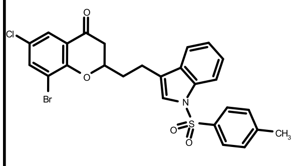	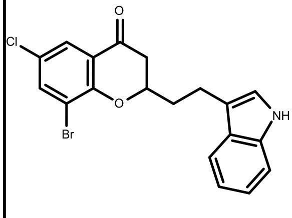
Dihydropyridines_derivative1_35c.mol	Dihydropyridines_derivative1_34c.mol	Chroman_4_one_derivative_3a.mol Chromone d	Chroman_4_one_derivative_1p.mol	Chroman_4_one_derivative_1o.mol

Bin cluster size of 1

41	42	43	44	45
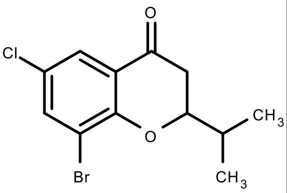	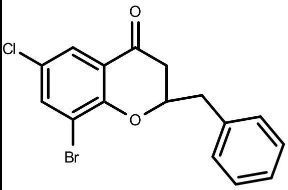	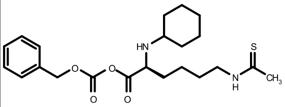	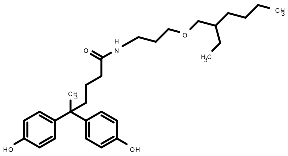	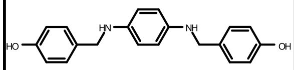
Chroman_4_one_derivative_1m.mol Chroman 4 one	Chroman_4_one_derivative_1l.mol Chroman 4 one	Cbz_K_thioacetyl_derv_1.mol Cbz K thioaceyl	piperidine_carboxamide.mol bis hydroxyphenyl	benzene_1_4_diamine_derv_12.mol
**46**	**47**	**48**	**49**	**50**
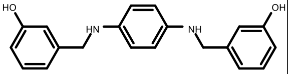	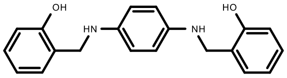	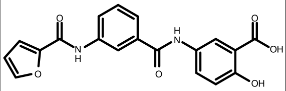	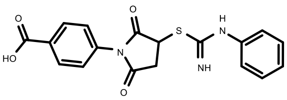	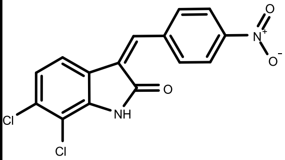
benzene_1_4_diamine_derv_11.mol	benzene_1_4_diamine_derv_10.mol	Untitled Document-9 BAS13555470.mol	Untitled Document-17 BAS00417531.mol	Arylideneindolinone_based_Inhibitor 9.mol
**51**	**52**	**53**	**54**	**55**
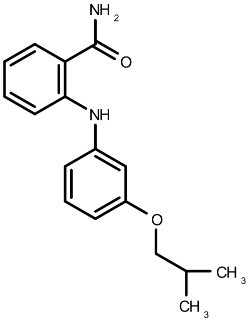	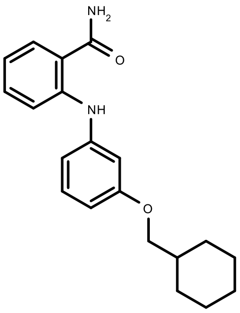	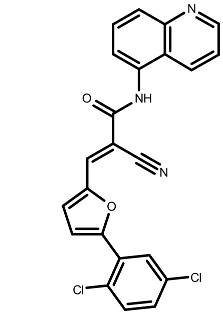	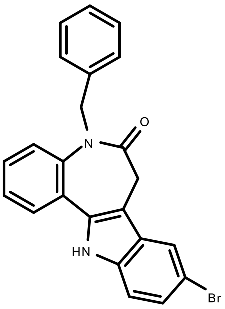	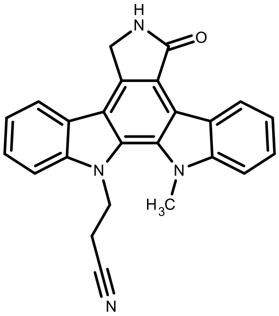
Anilinobenzamide_derivative1_31.mol	Anilinobenzamide_derivative1_30.mol	2130404 AGK2.mol	Adenosine_mimetics_15b.mol	Adenosine_mimetics_14b.mol
**56**	**57**	**58**	**59**	**60**
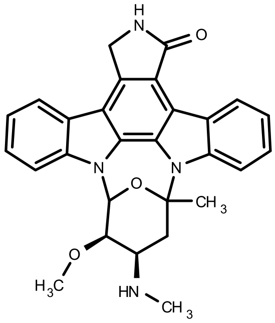	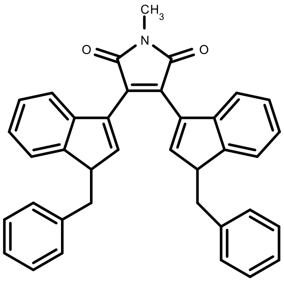	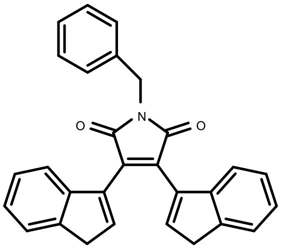	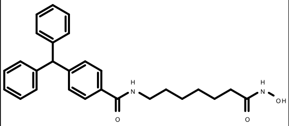	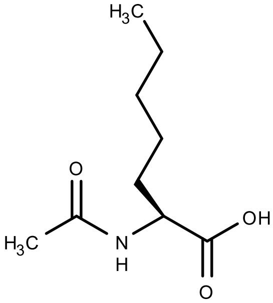
Adenosine_mimetics_14a.mol	Adenosine_mimetics_12d.mol	Adenosine_mimetics_12c.mol	ACY1215.mol	Acetyl_N_thioacetyl_lysine.mol

Bin cluster size of 1

61	62	63	64	65
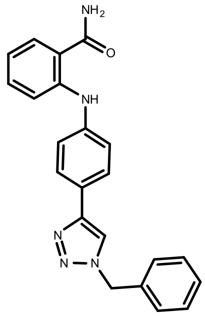	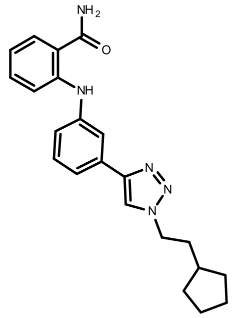	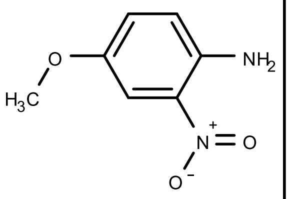	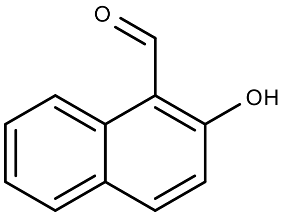	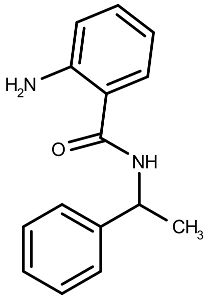
A1B11.mol A2B57 .mol	Untitled Document-1 A1B11.mol	2_amino_phenyl ethyl_benzamid e.mol 4 methox	iBET.mol 2_hydr oxy_1_napthalde hyde.mol	2_hydroxy_1_na pthaldehyde.mo l 2 amino phen

Bin cluster size of 2

1	2	3	4
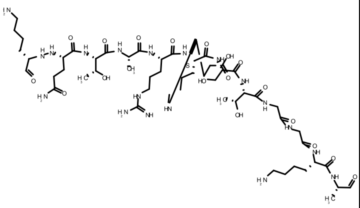	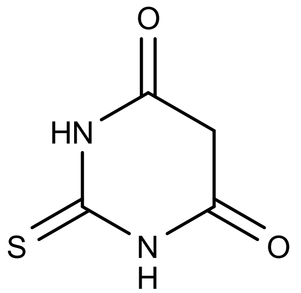	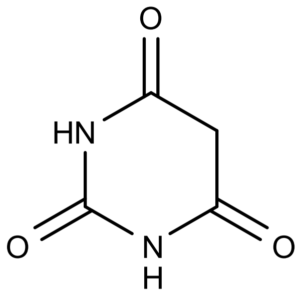	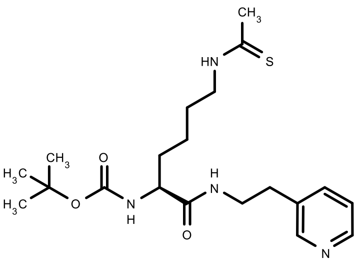
Thiosuccinyl peptide 1_H3K9TSu.mol	Thiobarbiturates_derivative1_31.mol	Thiobarbiturates_derivative1_30.mol	Thioacetylated_peptide_derivative_6.mol
**5**	**6**	**7**	**8**
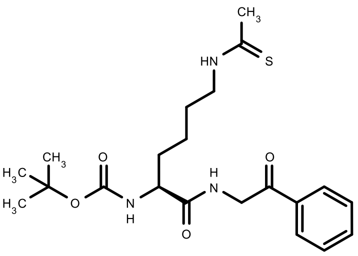	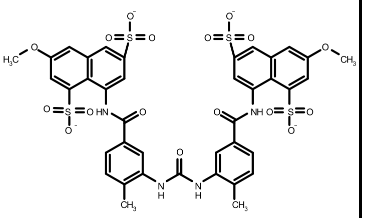	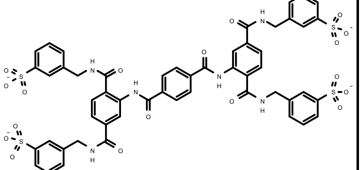	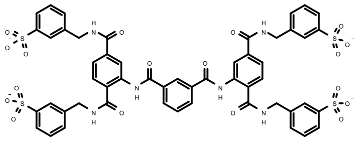
Thioacetylated_peptide_derivative_2.mol	Suramin_analogue_NF762.mol	Suramin_analogue_NF444.mol	Suramin_analogue_NF443.mol
**9**	**10**	**11**	**12**
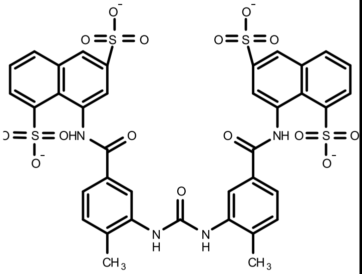	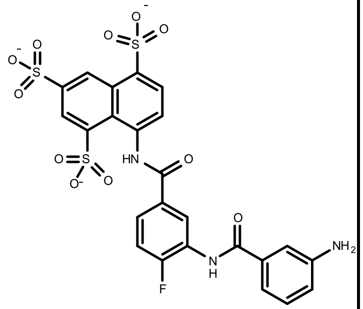	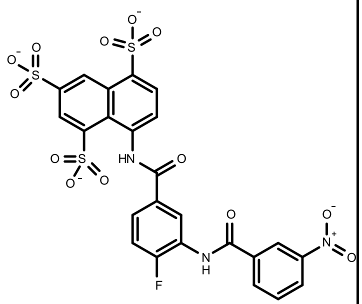	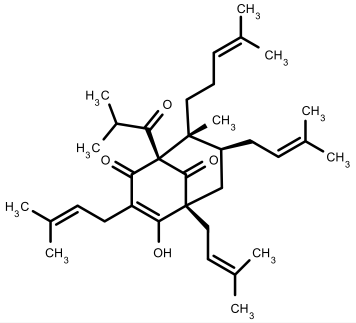
Suramin_analogue_NF290.mol	Suramin_analogue_NF155.mol	Suramin_analogue_NF154.mol	Hyperforin.mol
**13**	**14**	**15**	**16**
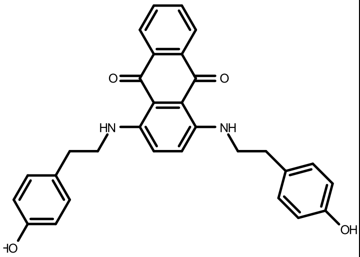	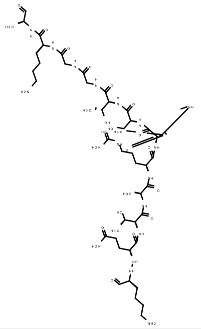	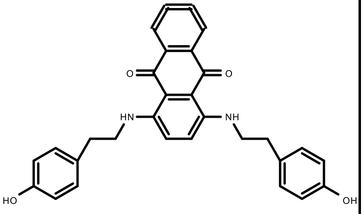	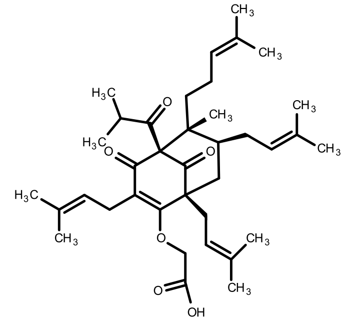
Hydroxyphenethylaminoanthracene.mol	H3K9TAc.mol	dihydroanthracene_dione.mol	Aristoforin.mol

Bin cluster size of 2

17	18	19	20
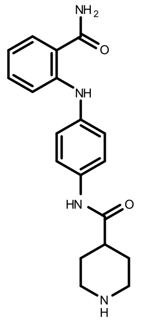	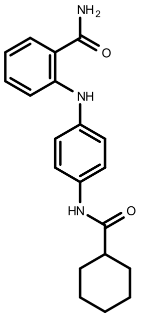	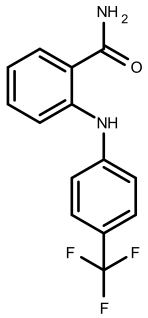	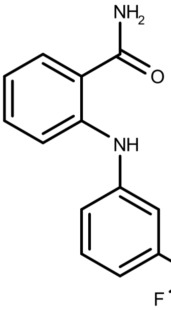
Anilinobenzamide_derivative1_18.mol	Anilinobenzamide_derivative1_17.mol	Anilinobenzamide_derivative1_7p.mol	Anilinobenzamide_derivative1_7d.mol
**21**	**22**	**23**	**24**
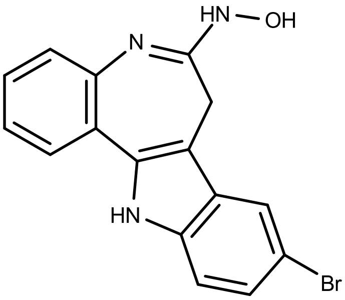	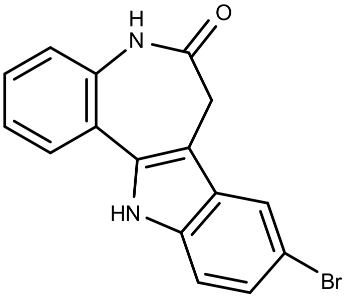	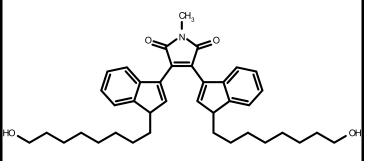	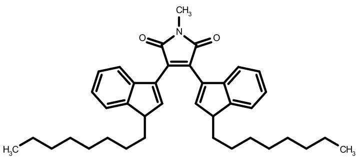
Adenosine_mimetics_15c.mol	Adenosine_mimetics_15a.mol	Adenosine_mimetics_12m.mol	Adenosine_mimetics_12k.mol
**25**	**26**	**27**	**28**
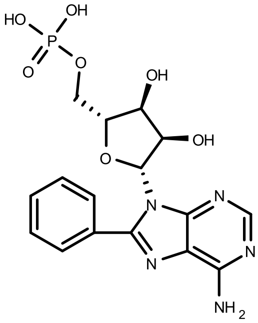	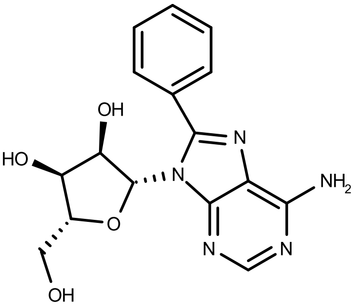	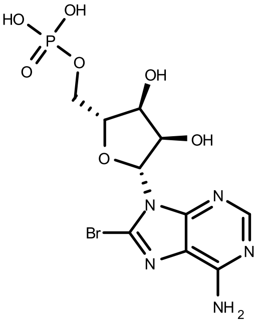	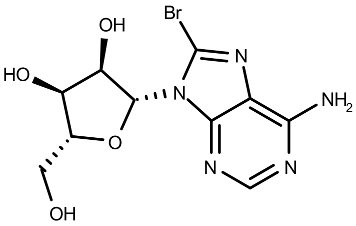
8_phenyl_AMP.mol	8_phenyl_adenosine.mol	8_bromo_AMP.mol	8_bromo_adenosine.mol

1

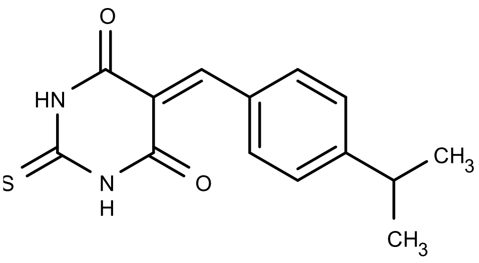

Thiobarbiturates_derivative1_29.mol

2

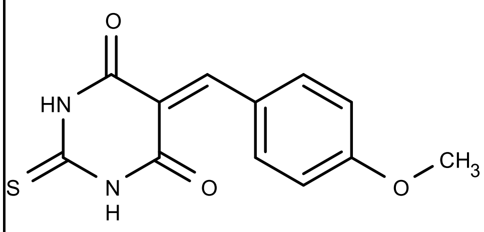

Thiobarbiturates_derivative1_28.mol

3

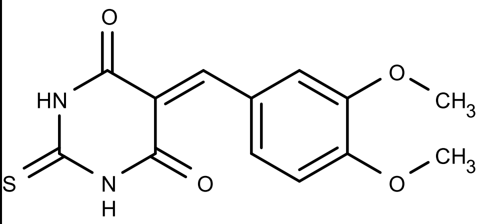

Thiobarbiturates_derivative1_27.mol

4

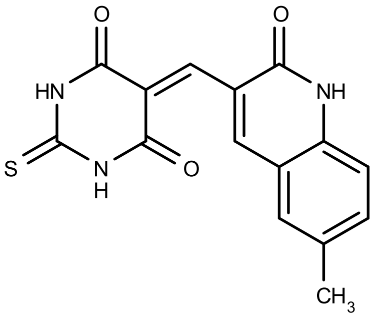

Thiobarbiturates_derivative1_8.mol

5

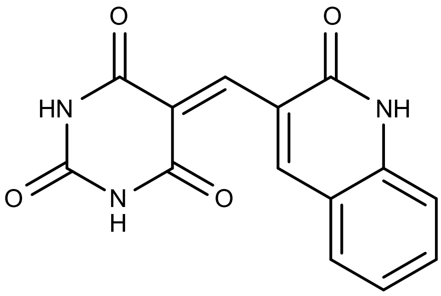

Thiobarbiturates_derivative1_7.mol

6

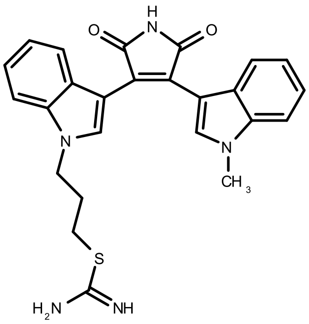

Thiobarbiturates_derivative1_5.mol

7

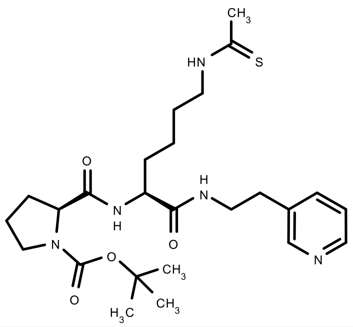

Thioacetylated_peptide_derivative_40.mol

8

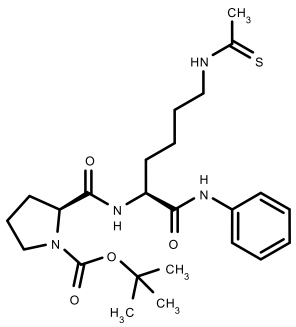

Thioacetylated_peptide_derivative_21.mol

9

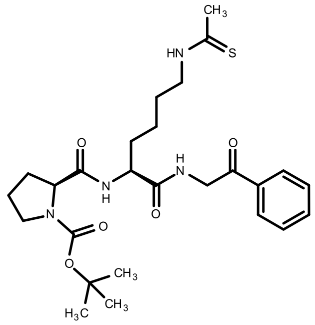

Thioacetylated_peptide_derivative_17.mol

10

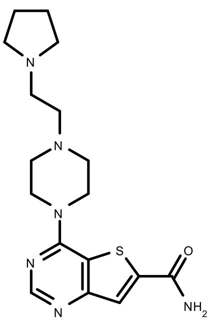

71654914 Thieno_pyrimidine_carboxamide_analog_35.mol

11

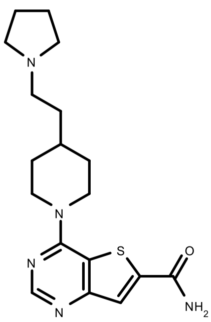

71654913 Thieno_pyrimidine_carboxamide_analog_34.mol

12

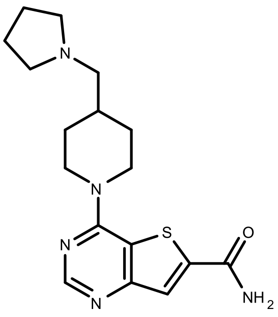

71654912 Thieno_pyrimidine_carboxamide_analog_33.mol

Bin cluster size of 3

13

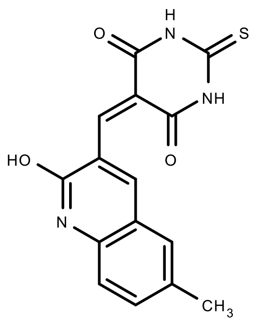

Tenovin_derivative1_16.mol

14

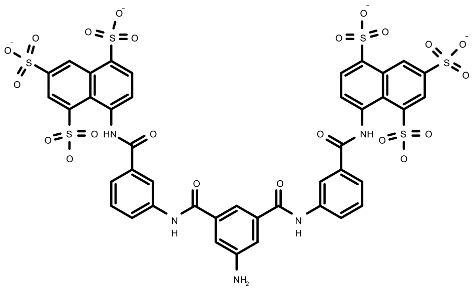

Suramin_analogue_NF675.mol

15

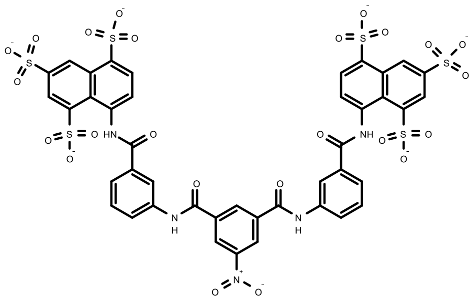

Suramin_analogue_NF674.mol

16

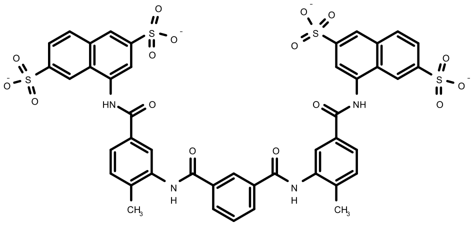

Suramin_analogue_NF342.mol

17

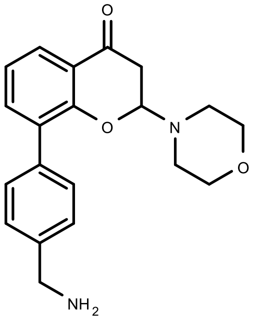

chromone_derv_2.mol chromone_derv_3.mol

18

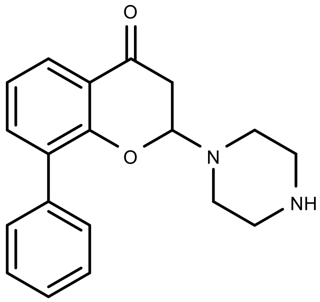

chromone_derv_1.mol chromone_derv_2.mol

19

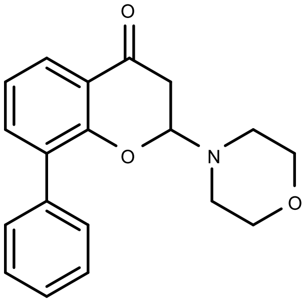

dimethoxy_quinaoline_derv_25.mol chromone_derv_1.mol

20

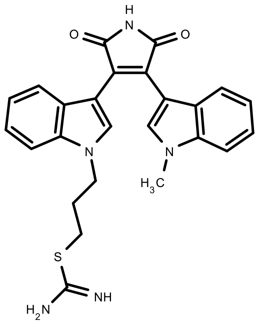

BIM-9 BIM_9.mol

21

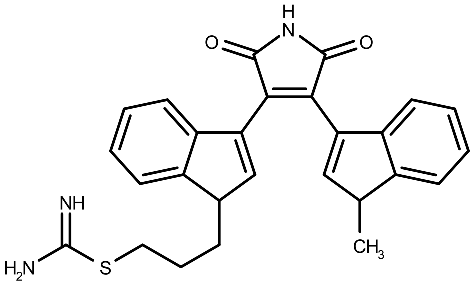

Adenosine_mimetics_12j.mol

1	2	3	4
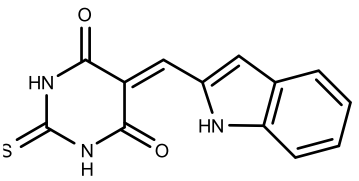	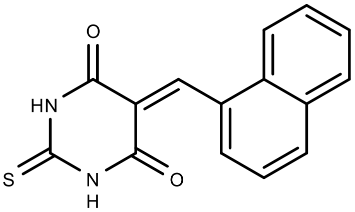	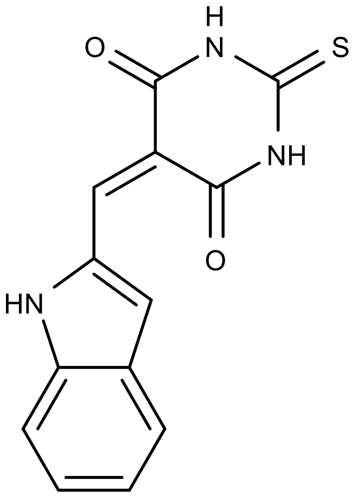	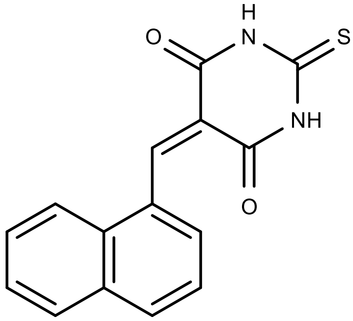
Thiobarbiturates_derivative1 26.mol	Thiobarbiturates_derivative1 9.mol	Tenovin_derivative1 18.mol	Tenovin_derivative1 17.mol
5	6	7	8
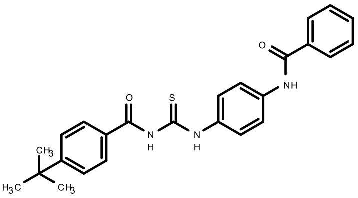	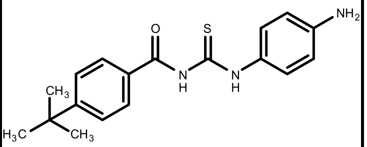	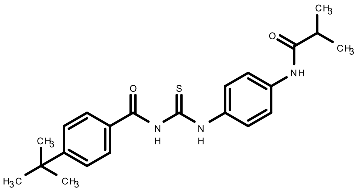	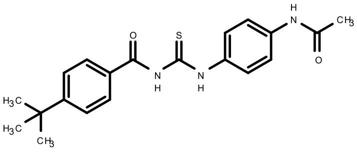
Tenovin_5.mol	Tenovin_3.mol	Tenovin_2.mol	Tenovin_1.mol
9	10	11	12
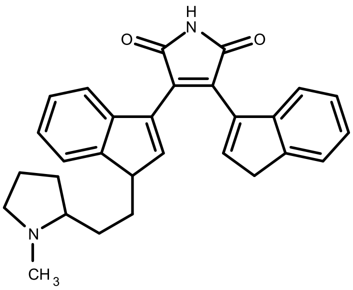	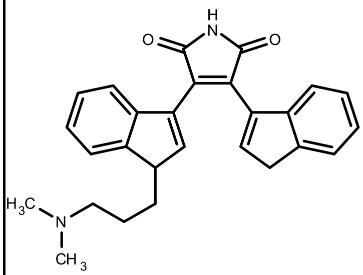	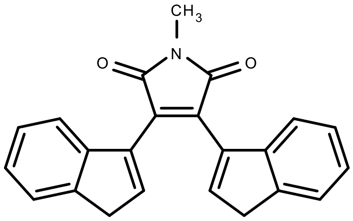	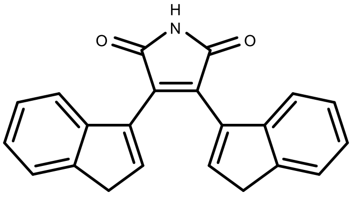
Adenosine_mimetics 12i.mol	Adenosine_mimetics 12h.mol	Adenosine_mimetics 12b.mol	Adenosine_mimetics 12a.mol

Bin cluster size of 5

1

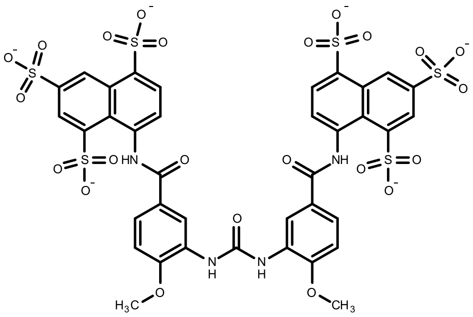

Suramin_analogue_NF259.mol

2

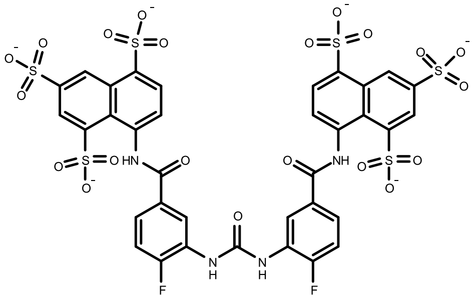

Suramin_analogue_NF156.mol

3

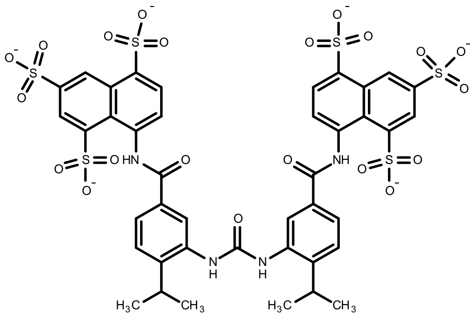

Suramin_analogue_NF150.mol

4

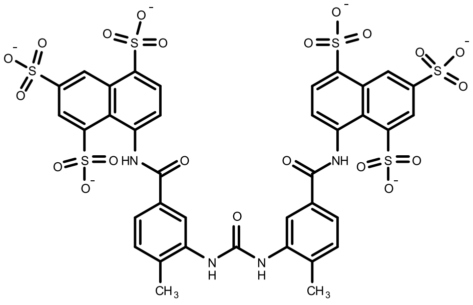

Suramin_analogue_NF058.mol

5

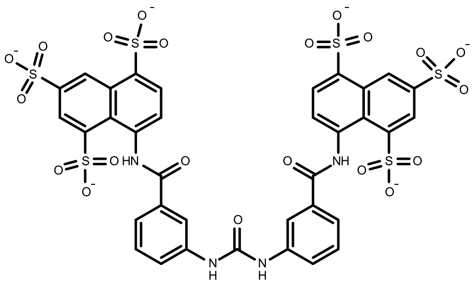

Suramin_analogue_NF023.mol

Bin cluster size of 6

1

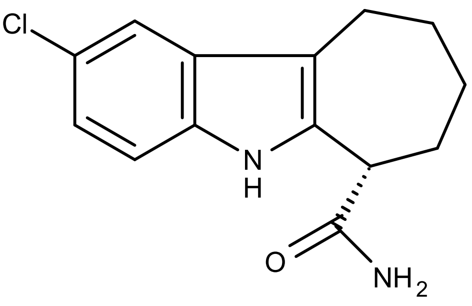

Thiobarbiturates_derivative1_4.mol

2

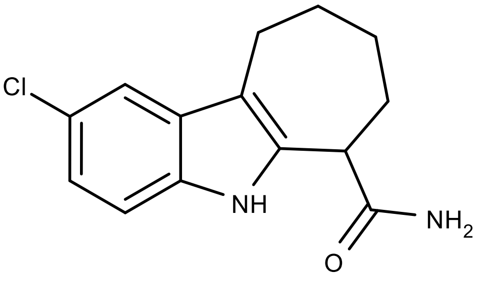

Tetrahydrocarbazole_derivative_35.mol

3

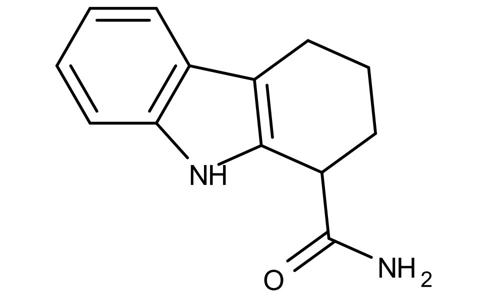

Tetrahydrocarbazole_derivative_3.mol

4

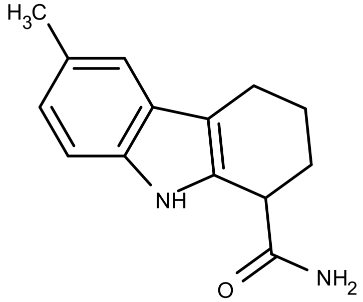

Tetrahydrocarbazole_derivative_2.mol

5

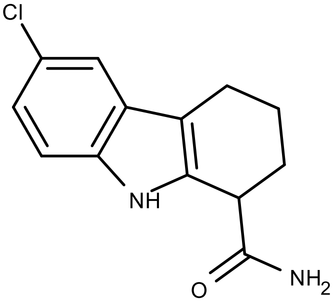

Tetrahydrocarbazole_derivative_1.mol

6

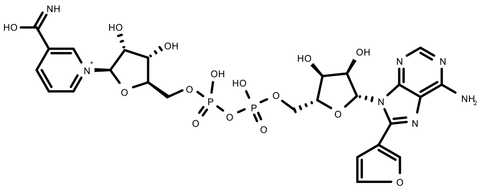

NAD_analog_6.mol

7

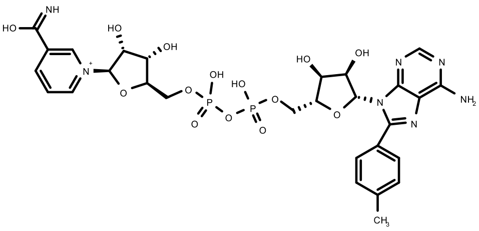

NAD_analog_5.mol

8

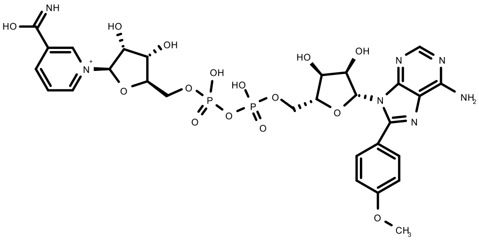

NAD_analog_4.mol

9

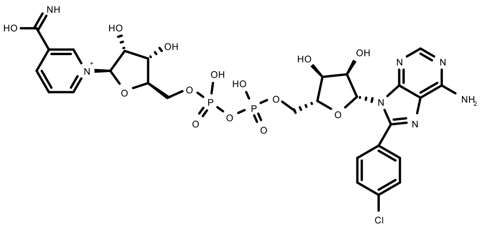

NAD_analog_3.mol

10

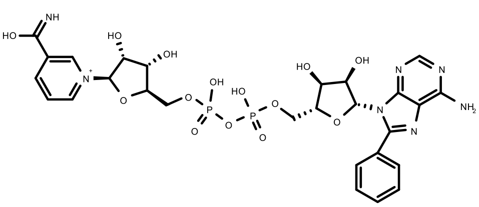

NAD_analog_2.mol

11

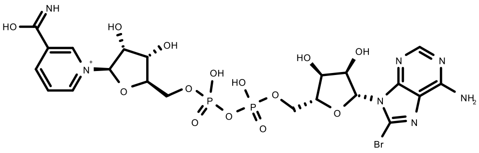

NAD_analog_1.mol

12

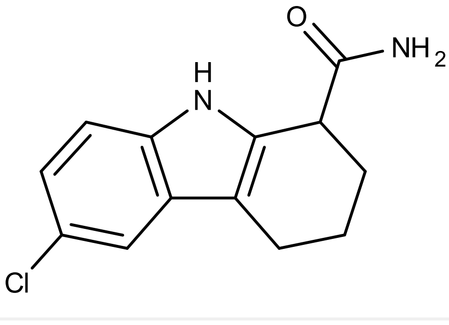

5113032 EX527.mol

13	14	15
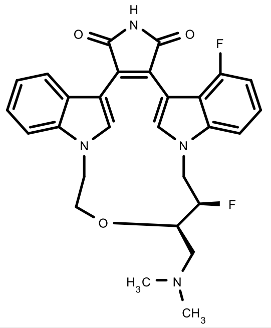	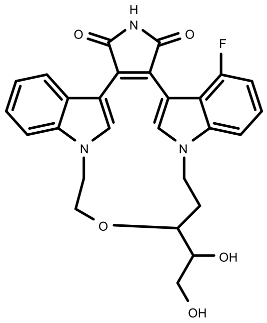	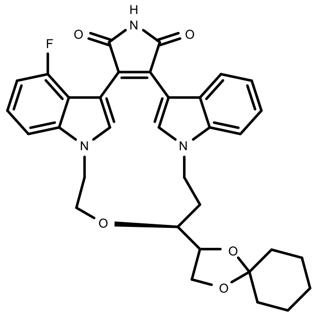
Adenosine_mimetics_13c.mol	Adenosine_mimetics_13b.mol	Adenosine_mimetics_13a.mol
**16**	**17**	**18**
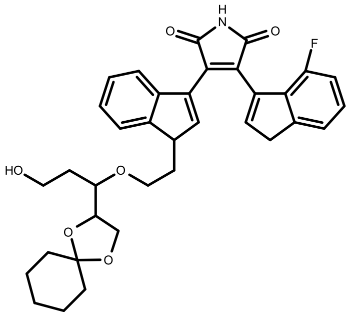	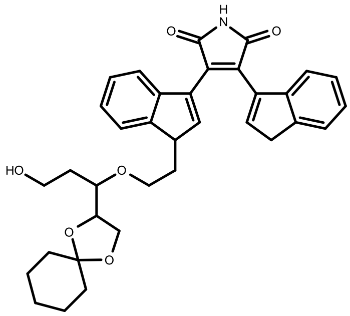	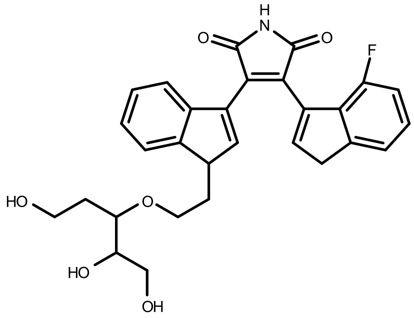
Adenosine_mimetics_12g.mol	Adenosine_mimetics_12f.mol	Adenosine_mimetics_12e.mol

Bin cluster size of 8

1	2	3	4
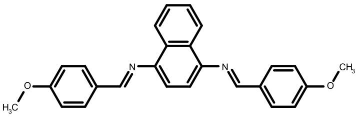	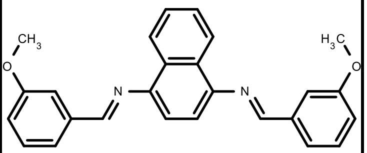	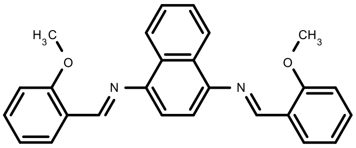	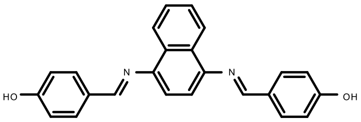
benzene_1_4_diamine_derv_19.mol	benzene_1_4_diamine_derv_18.mol	benzene_1_4_diamine_derv_17.mol	benzene_1_4_diamine_derv_16.mol
**5**	**6**	**7**	**8**
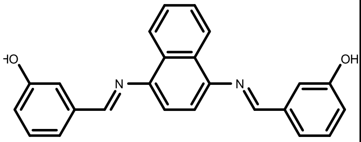	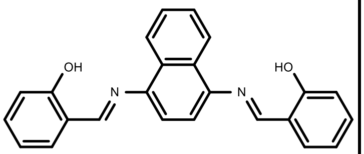	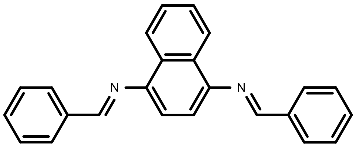	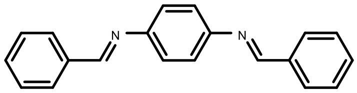
benzene_1_4_diamine_derv_15.mol	benzene_1_4_diamine_derv_14.mol	benzene_1_4_diamine_derv_13.mol	4_methoxy_nitroaniline.mol benzene_1_4_diamine_derv_1.mol

Bin cluster size of 9

1

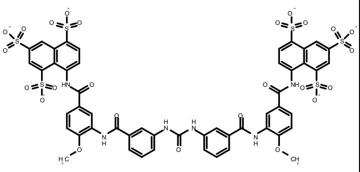

Suramin_analogue_NF260.mol

2

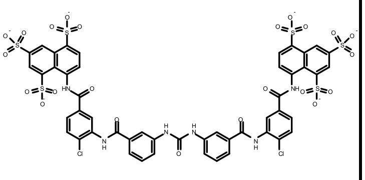

Suramin_analogue_NF258.mol

3

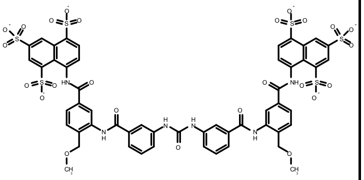

Suramin_analogue_NF222.mol

4

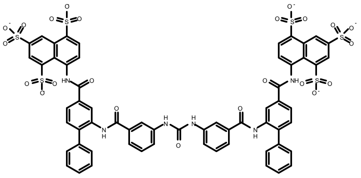

Suramin_analogue_NF198.mol

5

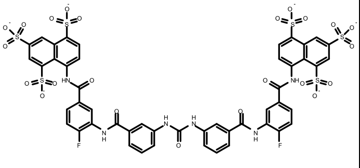

Suramin_analogue_NF157.mol

6

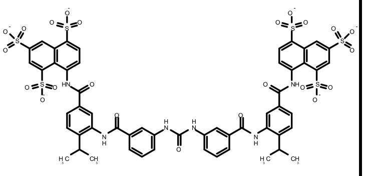

Suramin_analogue_NF151.mol

7

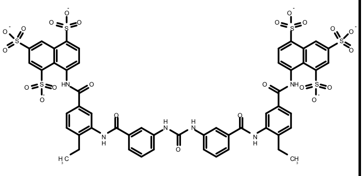

Suramin_analogue_NF127.mol

8

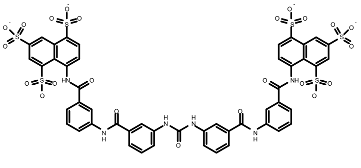

Suramin_analogue_NF037.mol

9

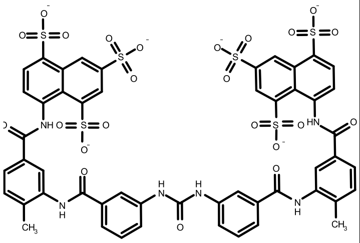

Suramin.mol

10

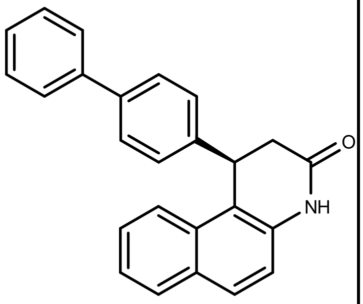

splitomicin_derv_51.mol

11

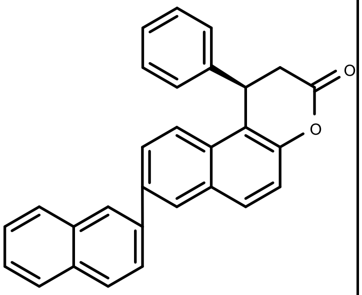

splitomicin_derv_43.mol

12

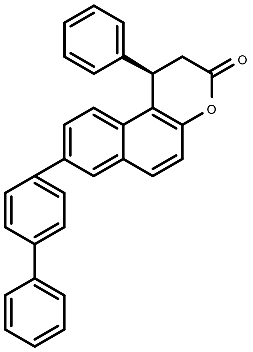

splitomicin_derv_42.mol

13

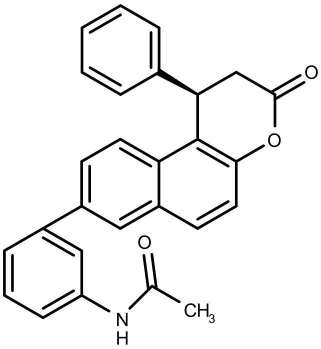

splitomicin_derv_41.mol

14

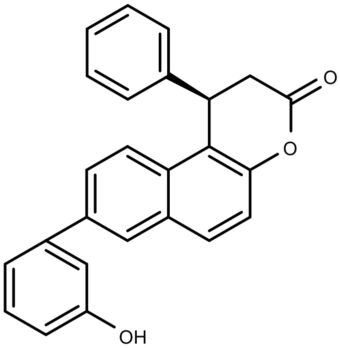

splitomicin_derv_40.mol

15

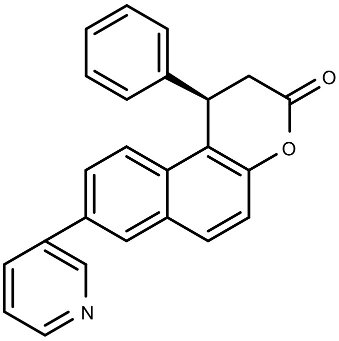

splitomicin_derv_39.mol

16

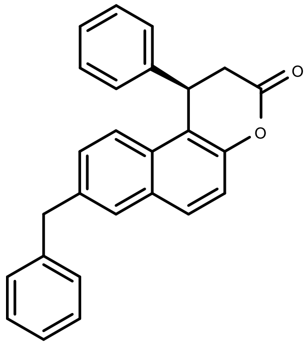

splitomicin_derv_37.mol

17	18		
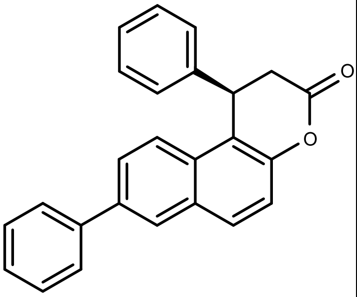	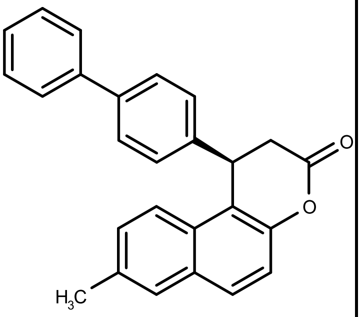 		
splitomicin_derv_36.mol	splitomicin_derv_30.mol		

Bin cluster size of 10

1

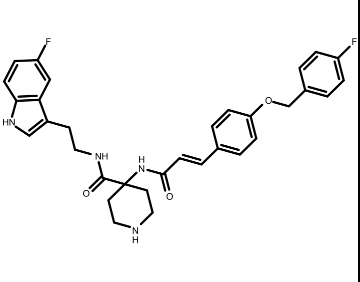

etrahydronaphthalen_derv.mol piperidine_carboxamide.mol

2

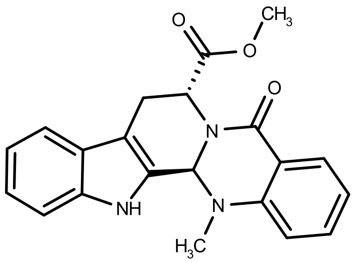

Evodiamine_derivative_ent_5.mol

3

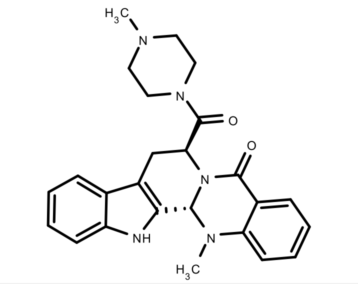

Evodiamine_derivative_12.mol

4

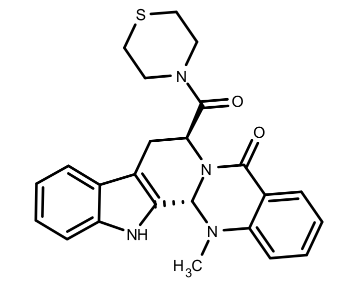

Evodiamine_derivative_11.mol

5

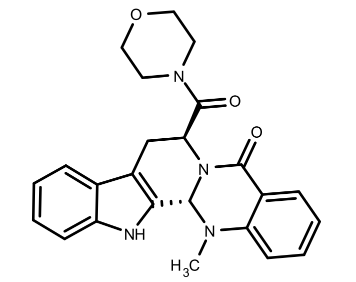

Evodiamine_derivative_10.mol

6

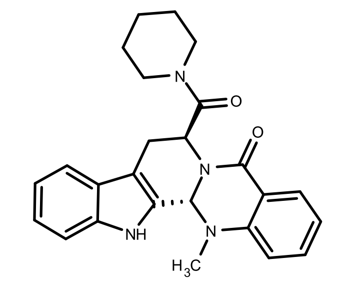

Evodiamine_derivative_9.mol

7

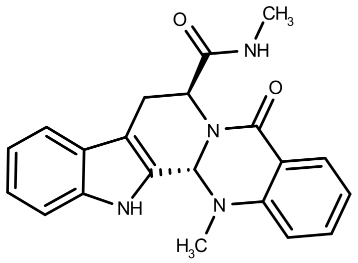

Evodiamine_derivative_8.mol

8

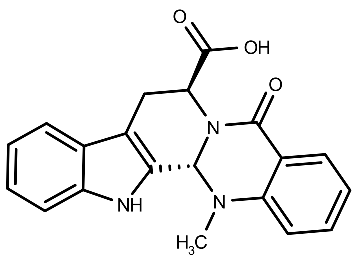

Evodiamine_derivative_7.mol

9

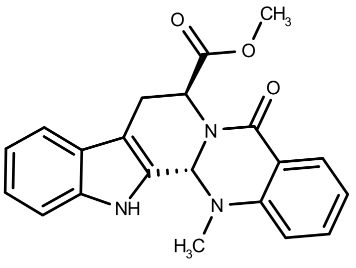

Evodiamine_derivative_5.mol

10

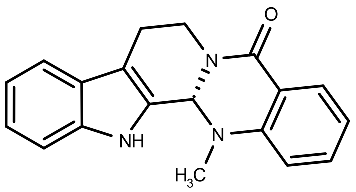

Evodiamine_derivative_1a.mol

11

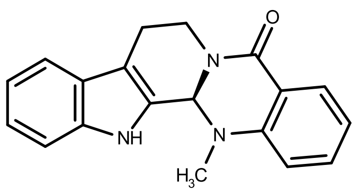

Evodiamine.mol

12

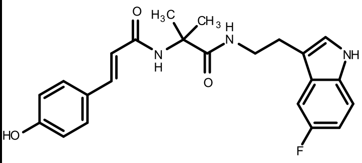

Amino_acid_tryptamide_derivative_7g.mol

13

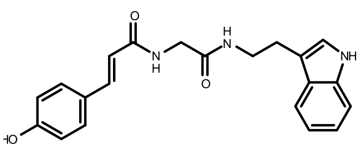

Amino_acid_tryptamide_derivative_7f.mol

14

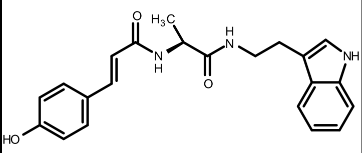

Amino_acid_tryptamide_derivative_7e_S.mol

15

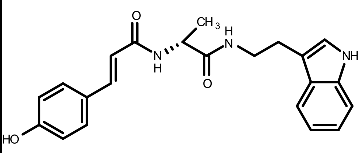

Amino_acid_tryptamide_derivative_7e_R.mol

16

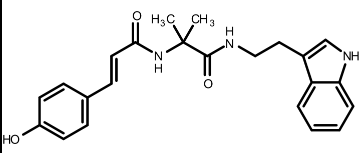

Amino_acid_tryptamide_derivative_7d.mol

17	18	19	20
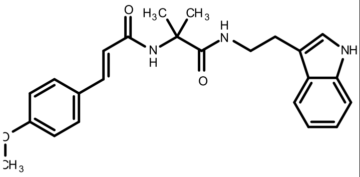	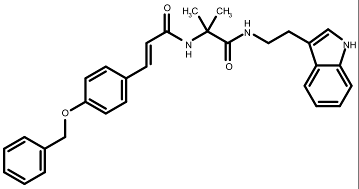	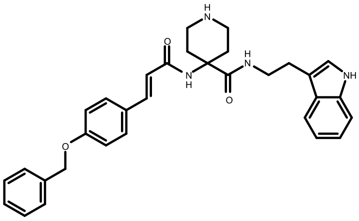	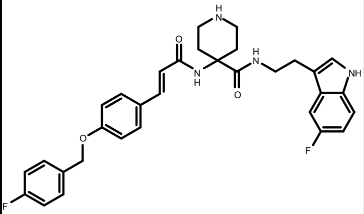
Amino_acid_tryptamide_derivative_7c.mol	Amino_acid_tryptamide_derivative_7b.mol	Amino_acid_tryptamide_derivative_7a.mol	Amino_acid_tryptamide_derivative_1.mol

Bin cluster size of 12

1	2	3	4
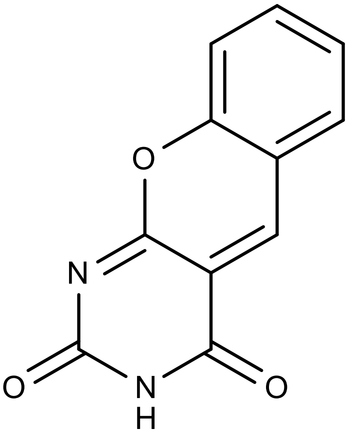	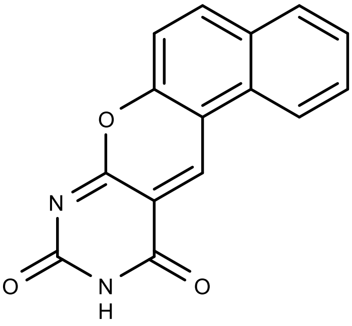	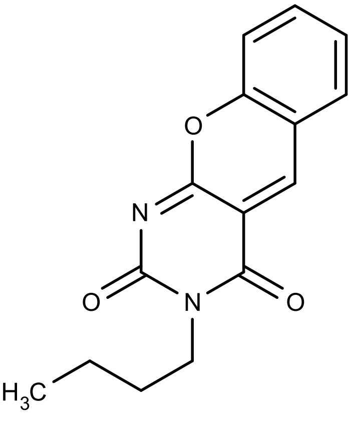	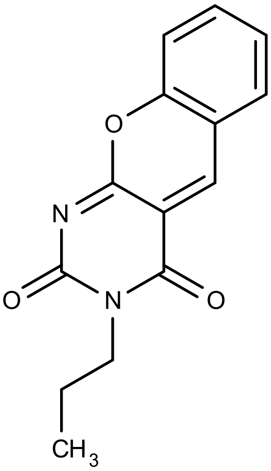
Pyrimidinediones_derivative1_9.mol	Pyrimidinediones_derivative1_7.mol	Benzodeazaoxaflavins_derivative1_3d.mol	Benzodeazaoxaflavins_derivative1_3c.mol
**5**	**6**	**7**	**8**
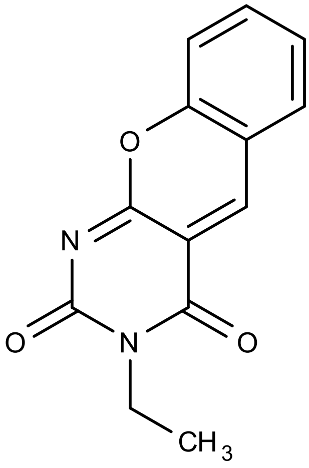	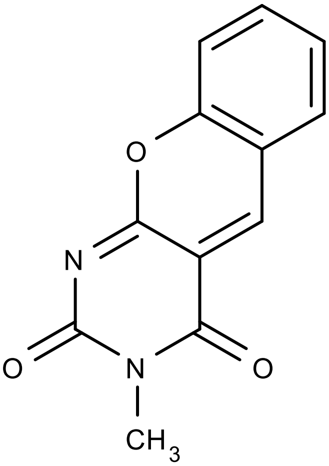	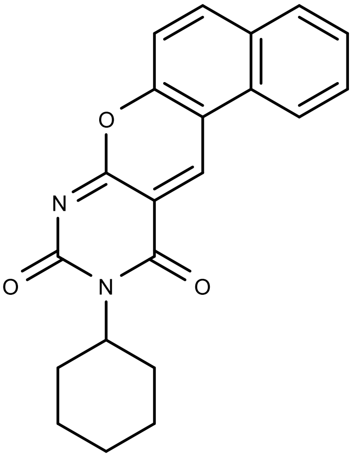	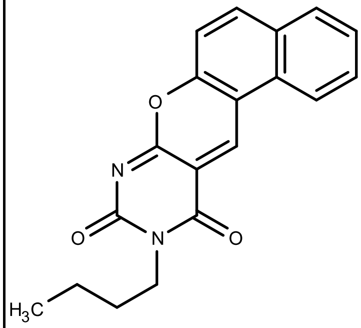
Benzodeazaoxaflavins_derivative1_3b.mol	Benzodeazaoxaflavins_derivative1_3a.mol	Benzodeazaoxaflavins_derivative1_2f.mol	Benzodeazaoxaflavins_derivative1_2e.mol
**9**	**10**	**11**	**12**
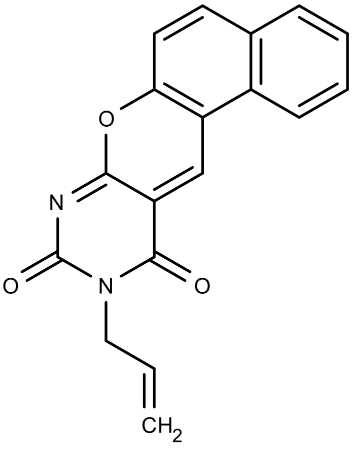	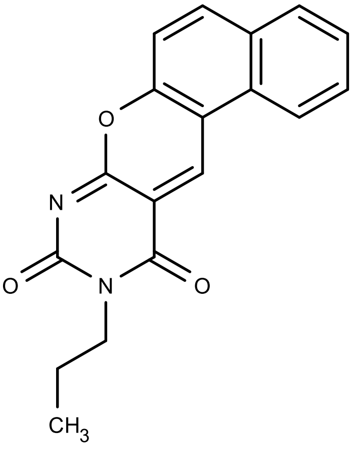	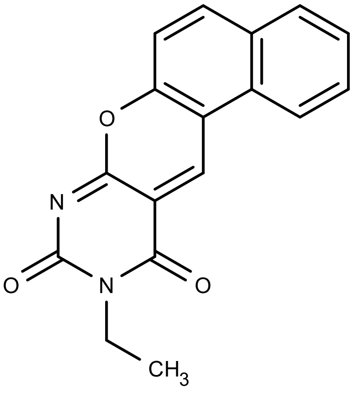	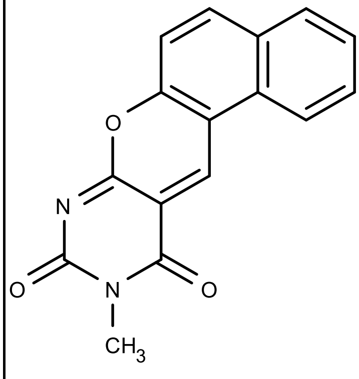
Benzodeazaoxaflavins_derivative1_2d.mol	Benzodeazaoxaflavins_derivative1_2c.mol	Benzodeazaoxaflavins_derivative1_2b.mol	Benzodeazaoxaflavins_derivative1_2a.mol

Bin cluster size of 13

1

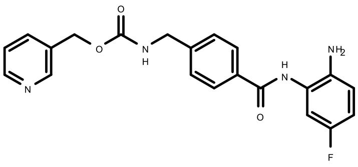

Benzamide_derivati
ve1 6h.mol

2

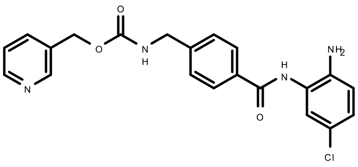

Benzamide_derivati
ve1 6g.mol

3

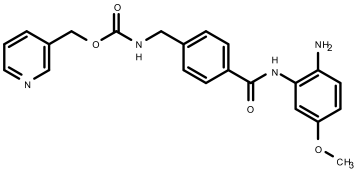

Benzamide_derivati
ve1 6f.mol

4

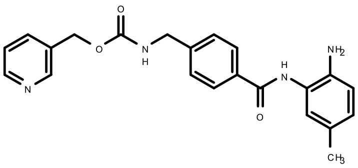

Benzamide_derivati
ve1 6e.mol

5

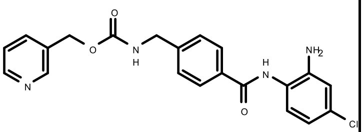

Benzamide_derivati
ve1 6d.mol

6

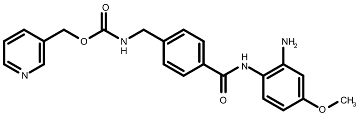

Benzamide_derivati
ve1 6c.mol

7

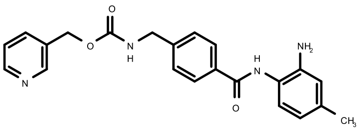

Benzamide_derivati
ve1 6b.mol

8

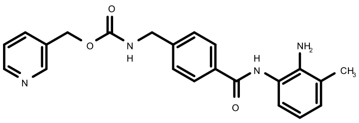

Benzamide_derivati
ve1 6a.mol

9

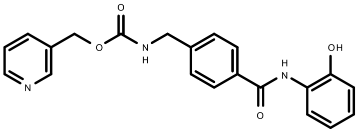

Benzamide_derivati
ve1 4e.mol

10

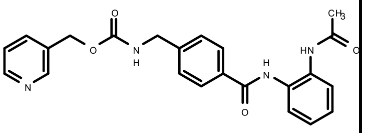

Benzamide_derivati
ve1 4d.mol

11

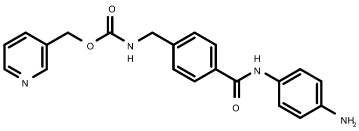

Benzamide_derivati
ve1 4c.mol

12

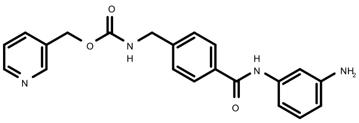

Benzamide_derivati
ve1 4b.mol

13

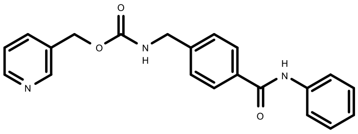

Benzamide_derivati
ve1 4a.mol

1

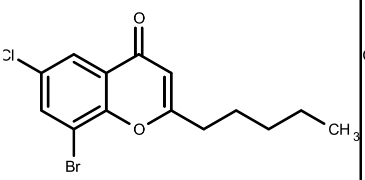

Chroman_4_one_derivative_1n.mol Chromone_derivative_3a.mol

2

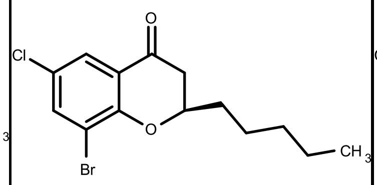

Untitled Document-1 Chroman_4_one_derivative_stereoS_1a.mol

3

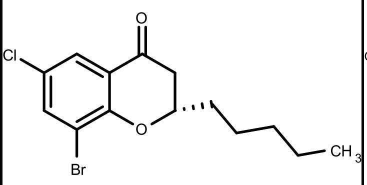

Chroman_4_one_derivative_stereo+_1a.mol Chroman_4_one_derivative_stereoR_1a.mol

4

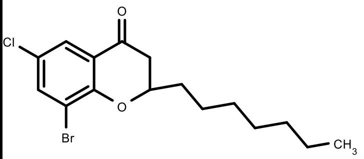

Chroman_4_one_derivative_1k.mol Chroman_4_one_derivative_1l.mol

5

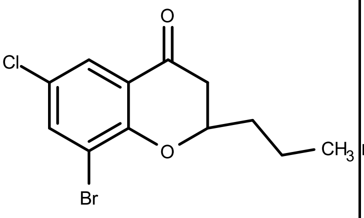

Chroman_4_one_derivative_1j.mol Chroman_4_one_derivative_1k.mol

6

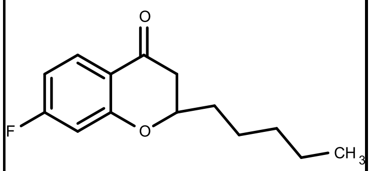

Chroman_4_one_derivative_1i.mol Chroman_4_one_derivative_1j.mol

7

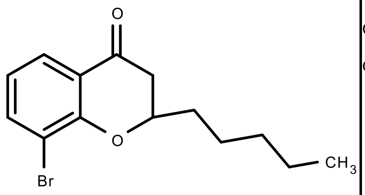

Chroman_4_one_derivative_1h.mol Chroman_4_one_derivative_1i.mol

8

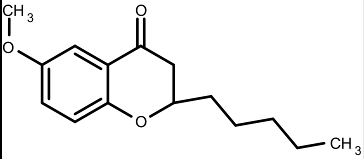

Chroman_4_one_derivative_1g.mol Chroman_4_one_derivative_1h.mol

9

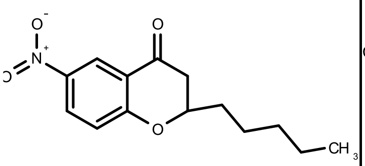

Chroman_4_one_derivative_1f.mol Chroman_4_one_derivative_1g.mol

10

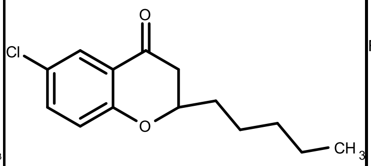

Chroman_4_one_derivative_1f.mol Chroman_4_one_derivative_1f.mol

11

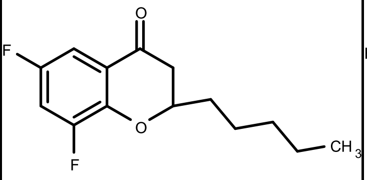

Chroman_4_one_derivative_1d.mol Chroman_4_one_derivative_1e.mol

12

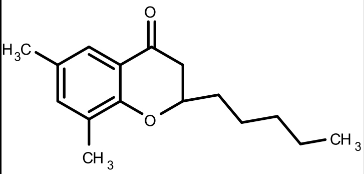

Chroman_4_one_derivative_1c.mol Chroman_4_one_derivative_1d.mol

13

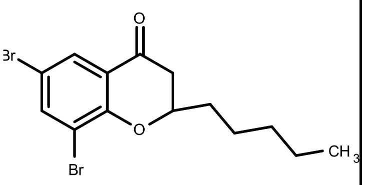

Chroman_4_one_derivative_1b.mol Chroman_4_one_derivative_1c.mol

14

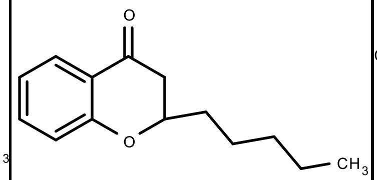

Chroman_4_one_derivative_1a.mol Chroman_4_one_derivative_1b.mol

15

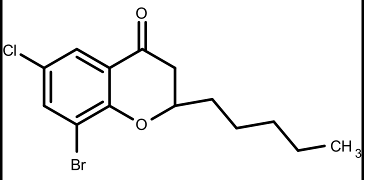

Chroman_4_one_derivative_stereo-_1a.mol Chroman_4_one_derivative_1a.mol

Bin cluster size of 17

1

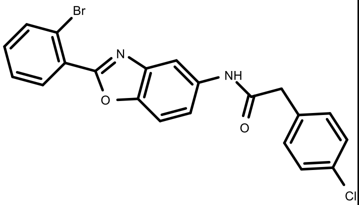

Phenyl_benzoxazole_derivative_16.mol Phenyl_benzoxazole_derivative_17.mol

2

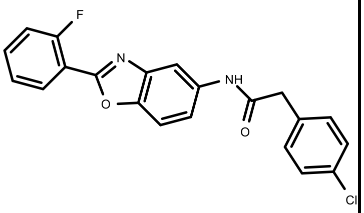

Phenyl_benzoxazole_derivative_15.mol Phenyl_benzoxazole_derivative_16.mol

3

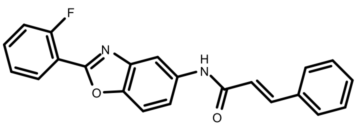

Phenyl_benzoxazole_derivative_14.mol Phenyl_benzoxazole_derivative_15.mol

4

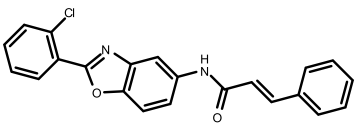

Phenyl_benzoxazole_derivative_13.mol Phenyl_benzoxazole_derivative_14.mol

5

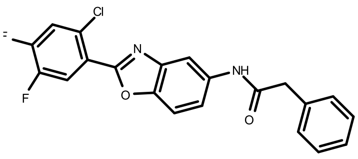

Phenyl_benzoxazole_derivative_12.mol Phenyl_benzoxazole_derivative_13.mol

6

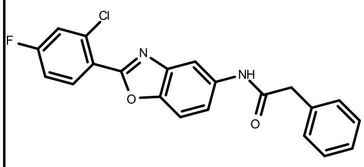

Phenyl_benzoxazole_derivative_11.mol Phenyl_benzoxazole_derivative_12.mol

7

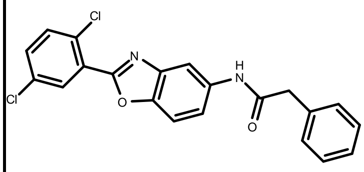

Phenyl_benzoxazole_derivative_10.mol Phenyl_benzoxazole_derivative_11.mol

8

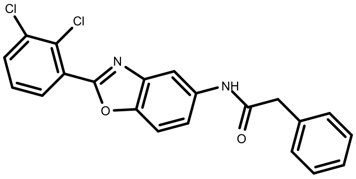

Phenyl_benzoxazole_derivative_9.mol Phenyl_benzoxazole_derivative_10.mol

9

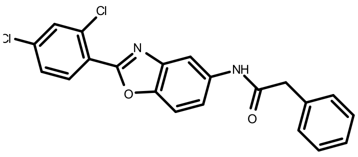

Phenyl_benzoxazole_derivative_8.mol Phenyl_benzoxazole_derivative_9.mol

10

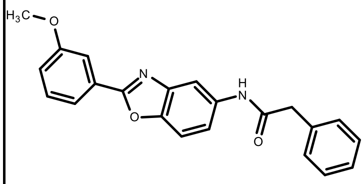

Phenyl_benzoxazole_derivative_7.mol Phenyl_benzoxazole_derivative_8.mol

11

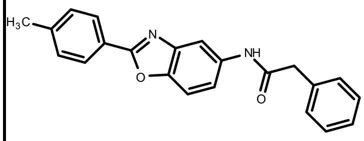

Phenyl_benzoxazole_derivative_6.mol Phenyl_benzoxazole_derivative_7.mol

12

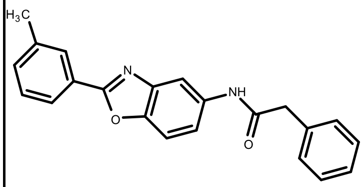

Phenyl_benzoxazole_derivative_5.mol Phenyl_benzoxazole_derivative_6.mol

13

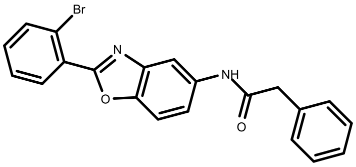

Phenyl_benzoxazole_derivative_4.mol Phenyl_benzoxazole_derivative_5.mol

14

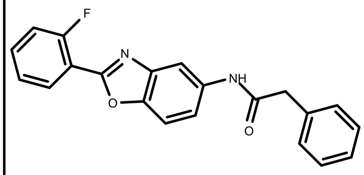

Phenyl_benzoxazole_derivative_3.mol Phenyl_benzoxazole_derivative_4.mol

15

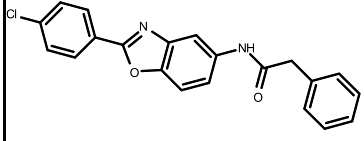

Phenyl_benzoxazole_derivative_2.mol Phenyl_benzoxazole_derivative_3.mol

16

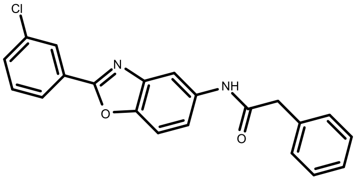

Phenyl_benzoxazole_derivative_1.mol Phenyl_benzoxazole_derivative_2.mol

17			
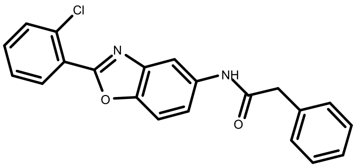			
Untitled Document-1 Phenyl_benzoxazole_derivative_1.mol			

1

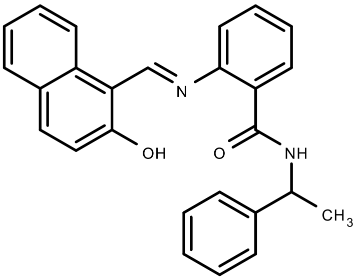

Thiobarbiturates_derivative1_1.mol

2

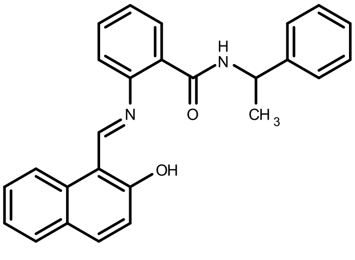

Sirtinol.mol

3

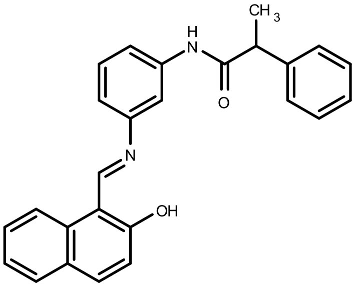

Salermide.mol

4

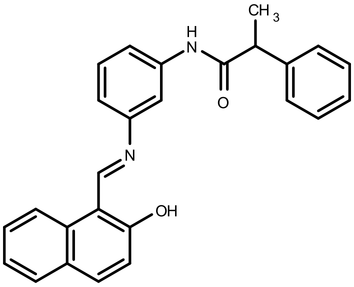

Pyrimidinediones_derivative1_salermide.mol

5

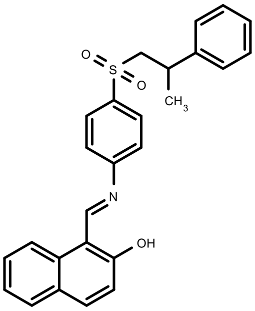

hydroxy_napthylmethylene_aminophenyl derv 15.mol

6

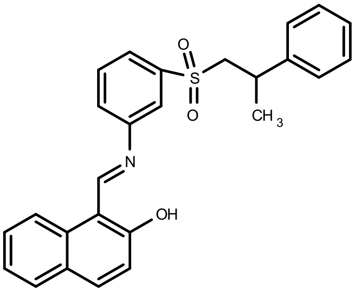

hydroxy_napthylmethylene_aminophenyl derv 14.mol

7

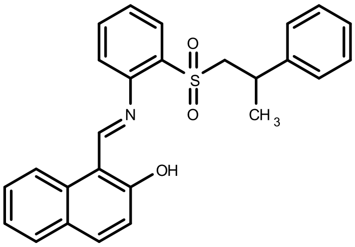

hydroxy_napthylmethylene_aminophenyl derv 13.mol

8

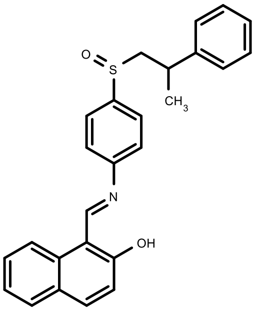

hydroxy_napthylmethylene_aminophenyl derv 12.mol

9

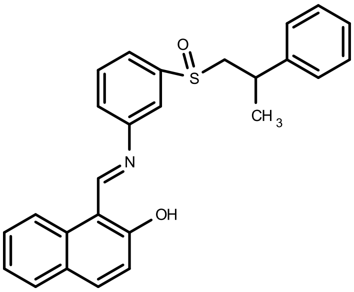

hydroxy_napthylmethylene_aminophenyl derv 11.mol

10

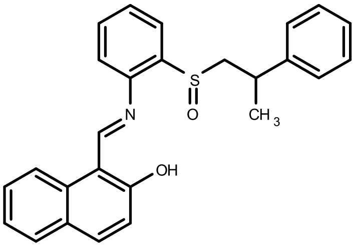

hydroxy_napthylmethylene_aminophenyl derv 10.mol

11

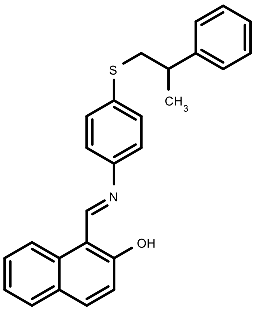

hydroxy_napthylmethylene_aminophenyl derv 9.mol

12

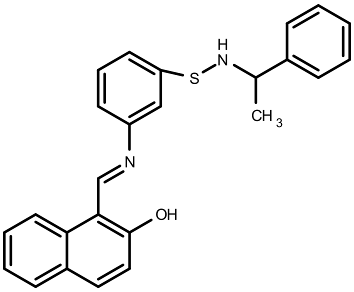

hydroxy_napthylmethylene_aminophenyl derv 8.mol

13

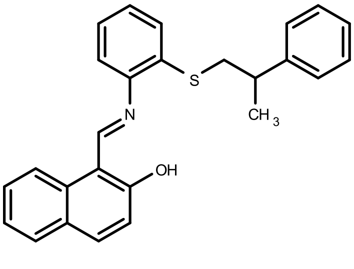

hydroxy_napthylmethylene_aminophenyl derv 7.mol

14

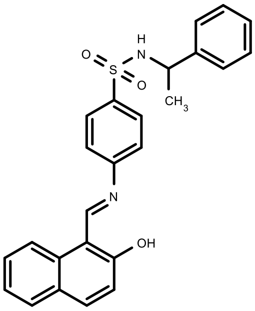

hydroxy_napthylmethylene_aminophenyl derv 6.mol

15

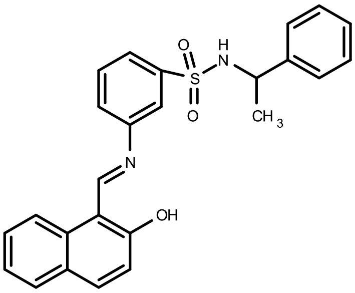

hydroxy_napthylmethylene_aminophenyl derv 5.mol

16

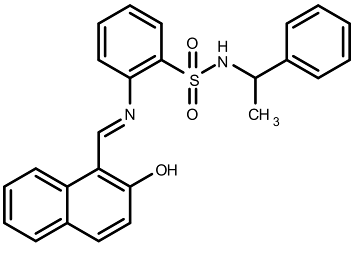

hydroxy_napthylmethylene_aminophenyl derv 4.mol

Bin cluster size of 19

17	18	19	
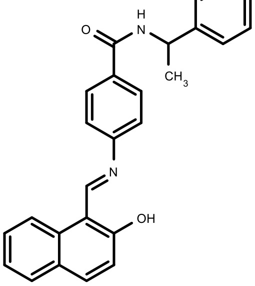	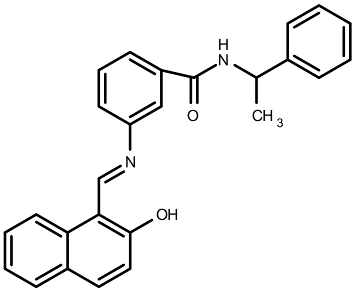	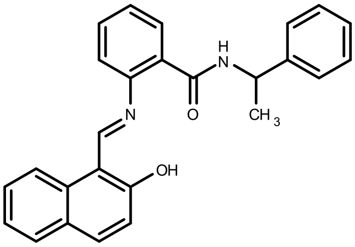	
hydroxy_napthylmethylene_aminophenyl derv 2.mol hydrox	hydroxy_napthylmethylene_aminophenyl derv 1.mol hydrox	Tripartin.mol hydroxy_napthylmethylene aminophenyl der	

Bin cluster size of 23

1

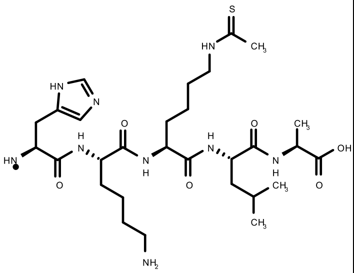

Thioacetyl_contain
ig peptide 23.mol

2

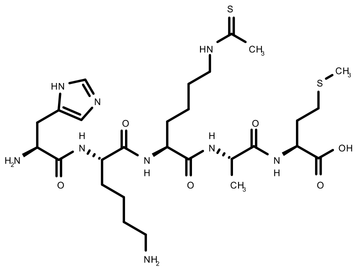

Thioacetyl_contain
ig peptide 22.mol

3

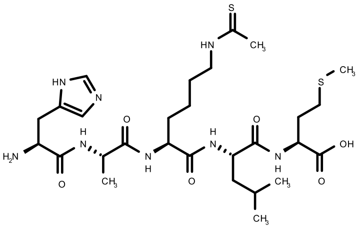

Thioacetyl_contain
ig peptide 21.mol

4

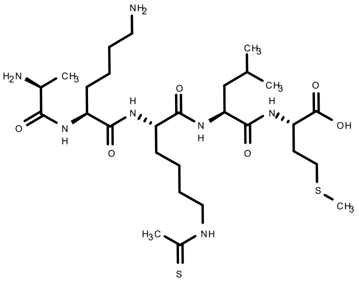

Thioacetyl_contain
ig peptide 20.mol

5

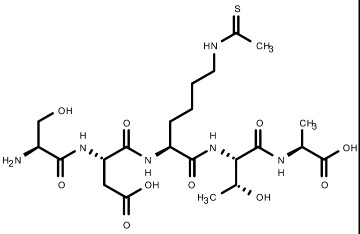

Thioacetyl_contain
ig peptide 19.mol

6

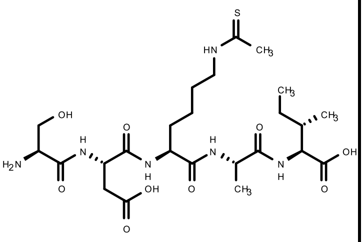

Thioacetyl_contain
ig peptide 18.mol

7

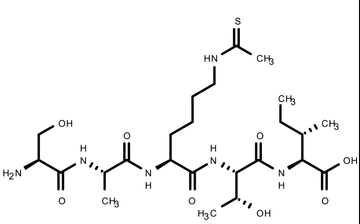

Thioacetyl_contain
ig peptide 17.mol

8

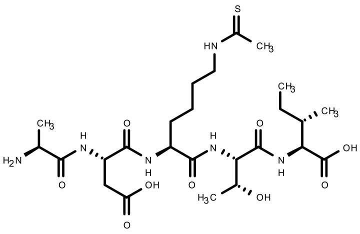

Thioacetyl_contain
ig peptide 16.mol

9

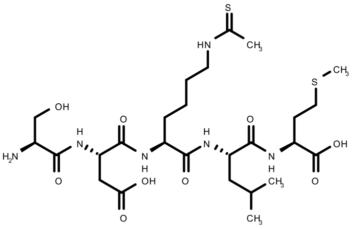

Thioacetyl_contain
ig peptide 15.mol

10

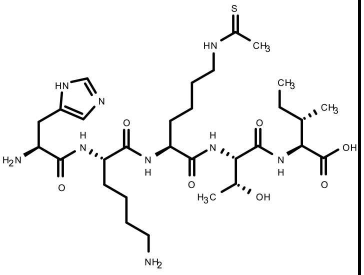

Thioacetyl_contain
ig peptide 14.mol

11

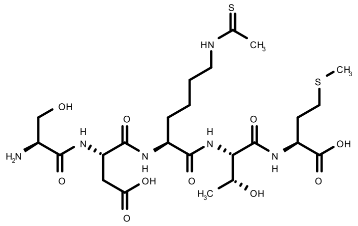

Thioacetyl_contain
ig peptide 13.mol

12

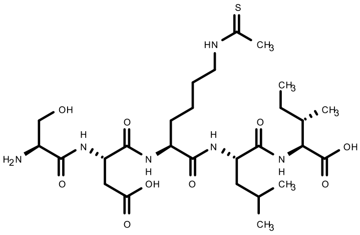

Thioacetyl_contain
ig peptide 12.mol

13

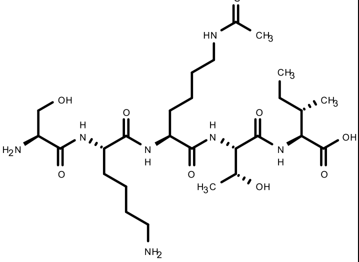

Thioacetyl_contain
ig peptide 11.mol

14

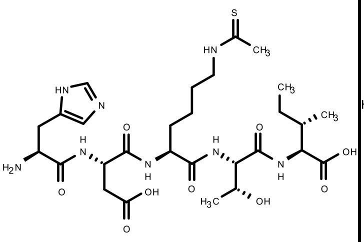

Thioacetyl_contain
ig peptide 10.mol

15

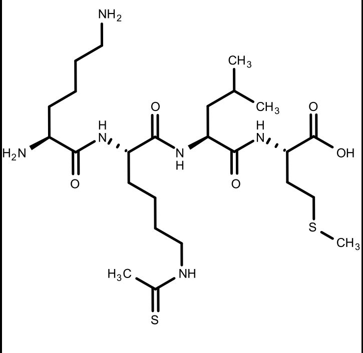

Thioacetyl_contain
ig peptide 8.mol

16

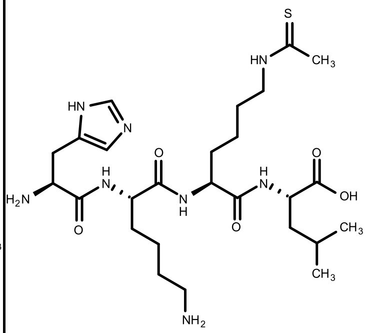

Thioacetyl_contain
ig peptide 7.mol

17

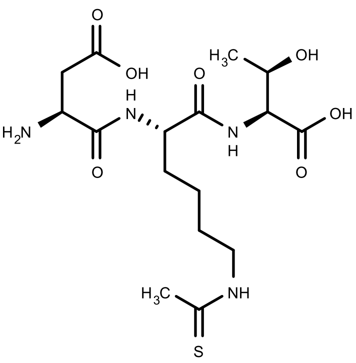

Thioacetyl_contain
ig peptide 4.mol

18

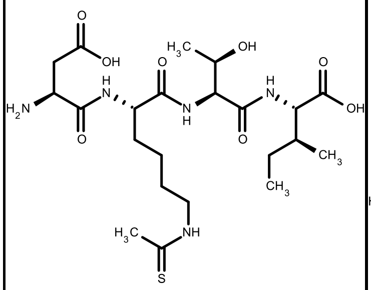

Thioacetyl_contain
ig peptide 3.mol

19

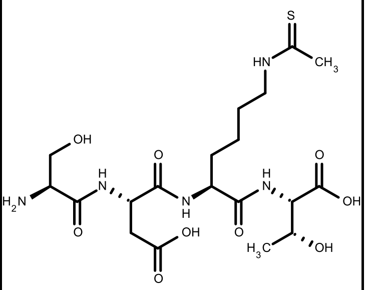

Thioacetyl_contain
ig peptide 2.mol

20

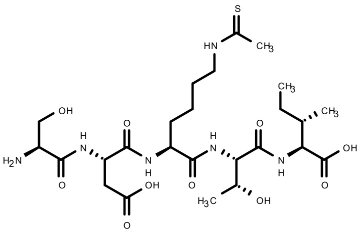

Thioacetyl_contain
ig peptide 1.mol

21

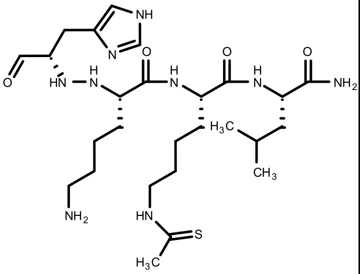

N_epsilon_Modified
lysine derivative

22

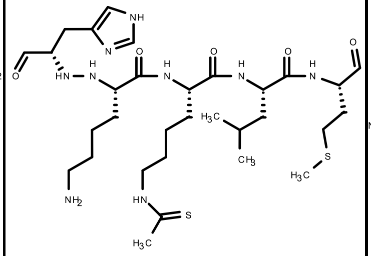

N_epsilon_Modified
lysine derivative

23

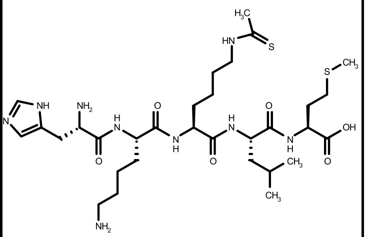

Untitled Document-
1 HKK thioaceyl LM

Bin cluster size of 28

1

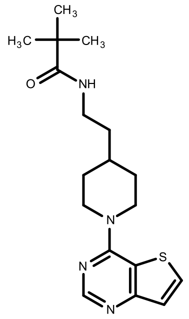

71654993 Thieno_pyrimidine_carboxamide analog 42.mol

2

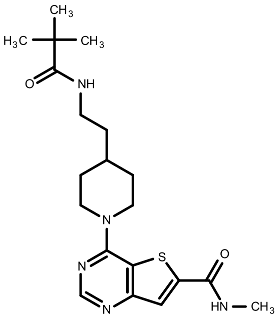

71654992 Thieno_pyrimidine_carboxamide analog 41.mol

3

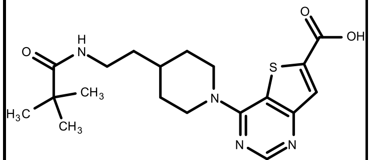

Thieno_pyrimidine_carboxamide_analog 40.mol

4

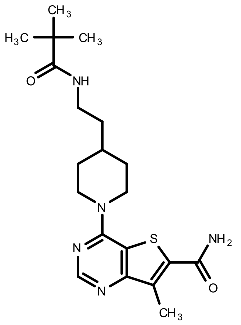

71654990 Thieno_pyrimidine_carboxamide analog 39.mol

5

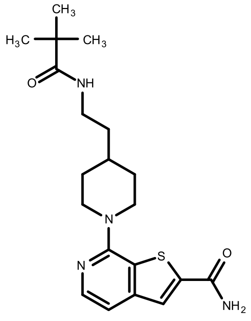

71654989 Thieno_pyrimidine_carboxamide analog 38.mol

6

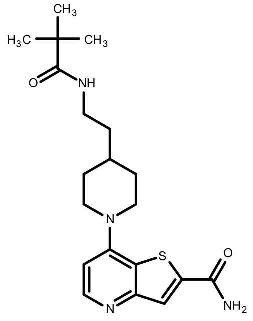

71654988 Thieno_pyrimidine_carboxamide analog 37.mol

7

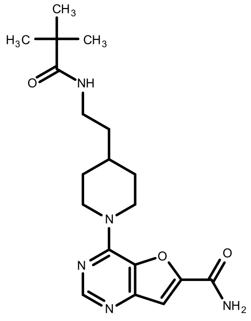

71654987 Thieno_pyrimidine_carboxamide analog 36.mol

8

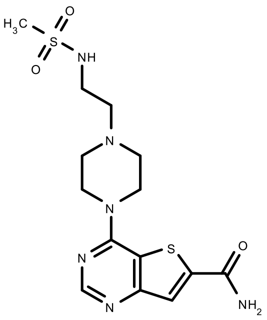

71654911 Thieno_pyrimidine_carboxamide analog 32.mol

9

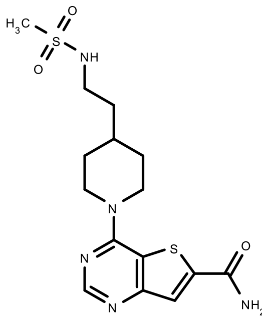

71304815 Thieno_pyrimidine_carboxamide analog 31.mol

10

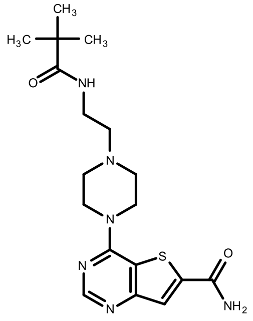

71654910 Thieno_pyrimidine_carboxamide analog 30.mol

11

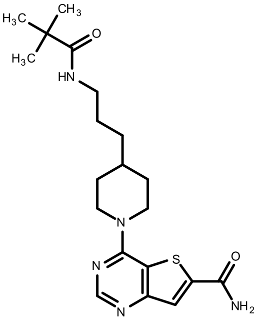

71654909 Thieno_pyrimidine_carboxamide analog 29.mol

12

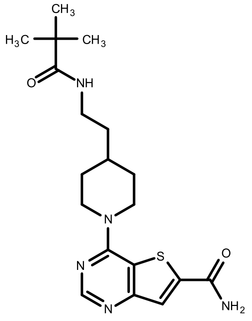

71304814 Thieno_pyrimidine_carboxamide analog 28.mol

13

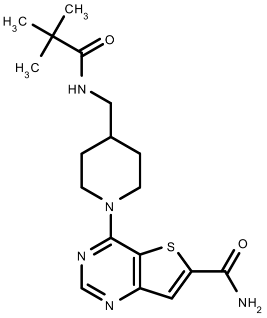

71654843 Thieno_pyrimidine_carboxamide analog 27.mol

14

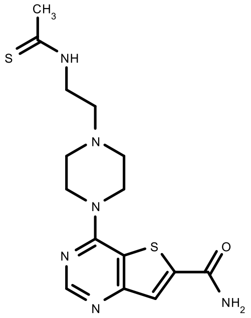

71654842 Thieno_pyrimidine_carboxamide analog 26.mol

15

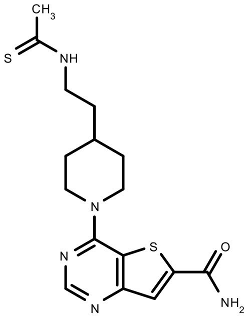

71654841 Thieno_pyrimidine_carboxamide analog 25.mol

16

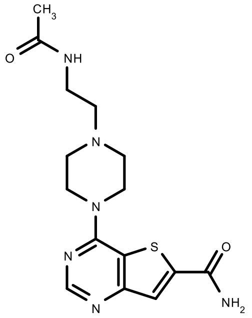

71654840 Thieno_pyrimidine_carboxamide analog 24.mol

Bin cluster size of 28

17

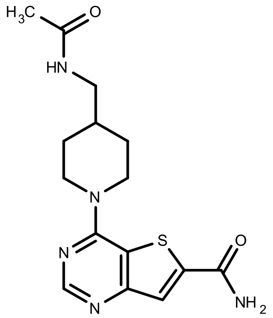

71654839 Thieno_pyrimidine_carboxamide analog 23.mol

18

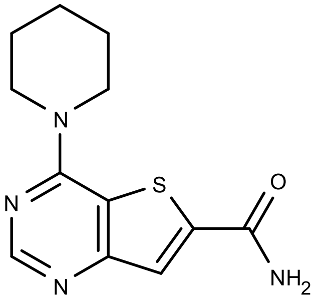

71654775 Thieno_pyrimidine_carboxamide analog 21.mol

19

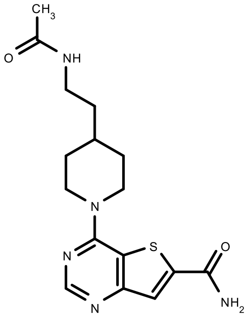

71654774 Thieno_pyrimidine_carboxamide analog 20.mol

20

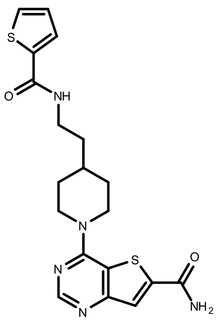

71654773 Thieno_pyrimidine_carboxamide analog 19.mol

21

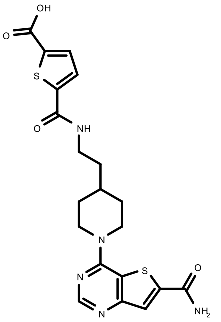

71654772 Thieno_pyrimidine_carboxamide analog 18.mol

22

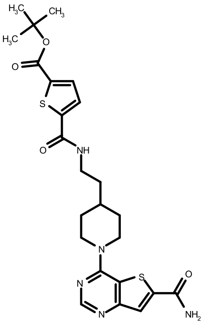

71654771 Thieno_pyrimidine_carboxamide analog 17.mol

23

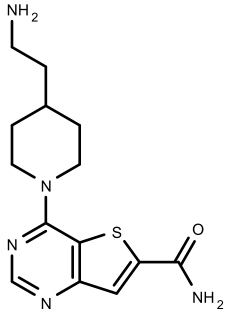

71655141 Thieno_pyrimidine_carboxamide analog 16a.mol

24

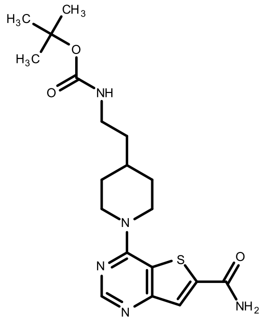

71655063 Thieno_pyrimidine_carboxamide analog 15a.mol

25

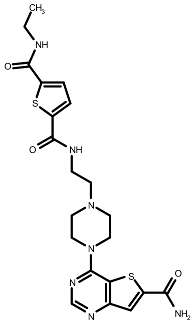

71655062 Thieno_pyrimidine_carboxamide analog 11d.mol

26

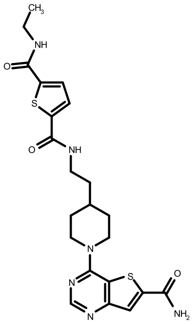

71304813 Thieno_pyrimidine_carboxamide analog 11c.mol

27

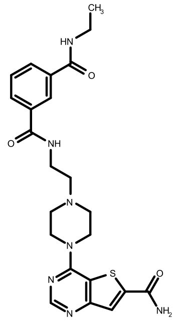

71655061 Thieno_pyrimidine_carboxamide analog 11b.mol

28

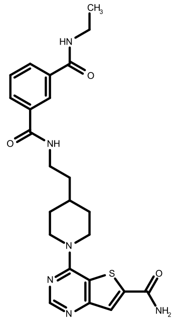

71655060 Thieno_pyrimidine_carboxamide analog 11a.mol

1

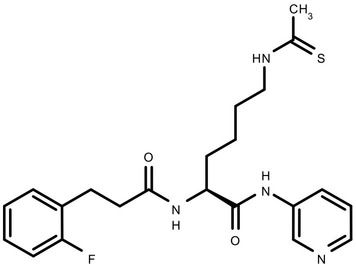

Thioacetylated_peptide_derivative_39.mol

2

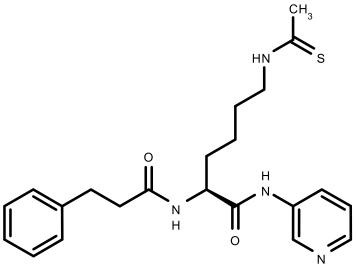

Thioacetylated_peptide_derivative_38.mol

3

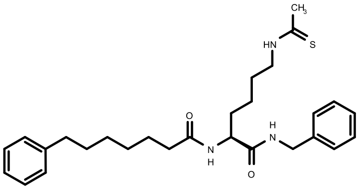

Thioacetylated_peptide_derivative_37.mol

4

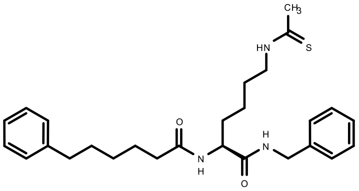

Thioacetylated_peptide_derivative_36.mol

5

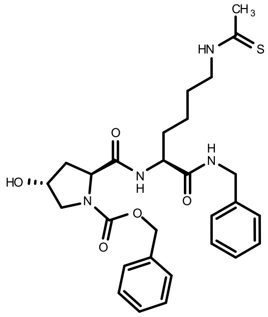

Thioacetylated_peptide_derivative_35.mol

6

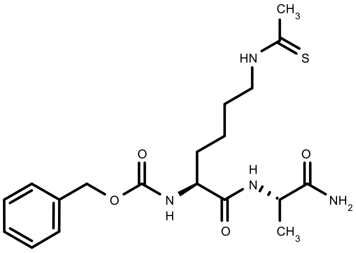

Thioacetylated_peptide_derivative_34.mol

7

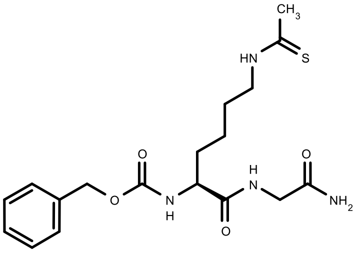

Thioacetylated_peptide_derivative_33.mol

8

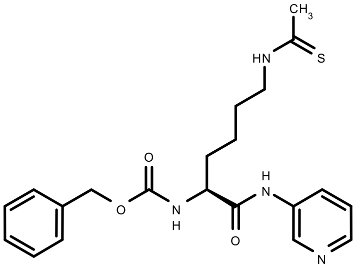

Thioacetylated_peptide_derivative_32.mol

9

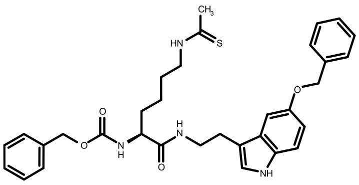

Thioacetylated_peptide_derivative_31.mol

10

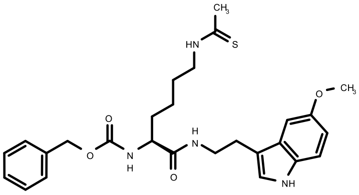

Thioacetylated_peptide_derivative_30.mol

11

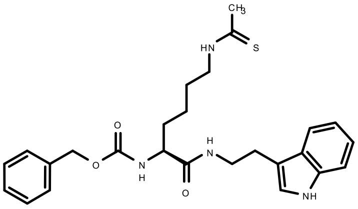

Thioacetylated_peptide_derivative_29.mol

12

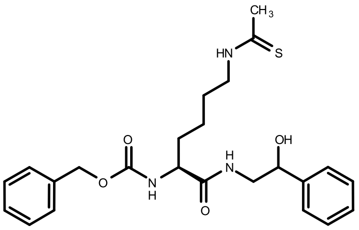

Thioacetylated_peptide_derivative_28.mol

13

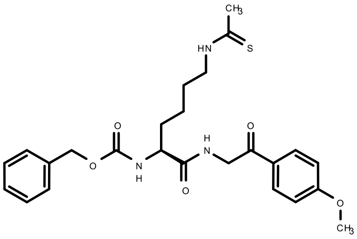

Thioacetylated_peptide_derivative_27.mol

14

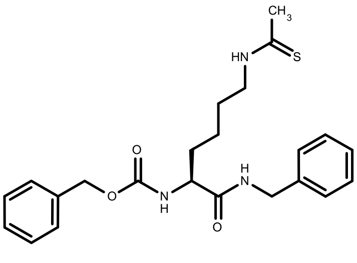

Thioacetylated_peptide_derivative_26.mol

15

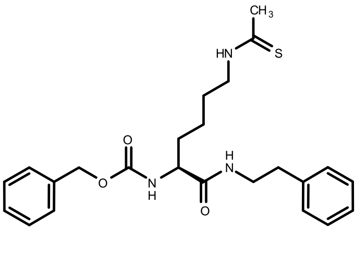

Thioacetylated_peptide_derivative_25.mol

16

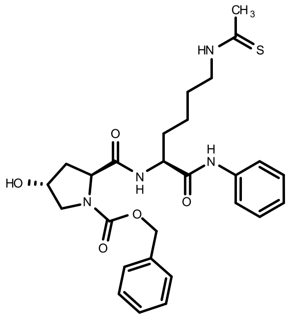

Thioacetylated_peptide_derivative_23.mol

Bin cluster size of 37

17

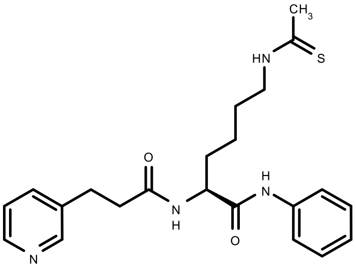

Thioacetylated_peptide_derivative_19.mol

18

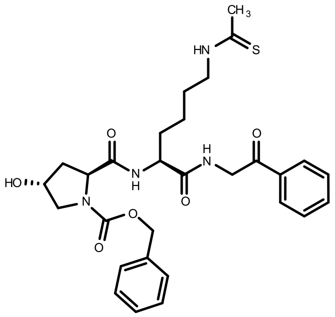

Thioacetylated_peptide_derivative_16.mol

19

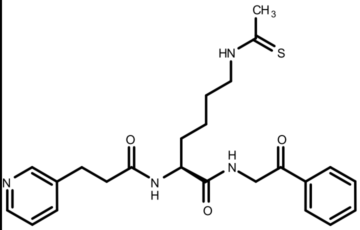

Thioacetylated_peptide_derivative_15.mol

20

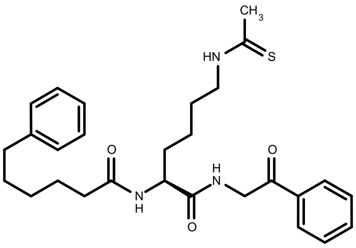

Thioacetylated_peptide_derivative_14.mol

21

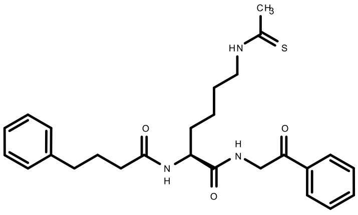

Thioacetylated_peptide_derivative_13.mol

22

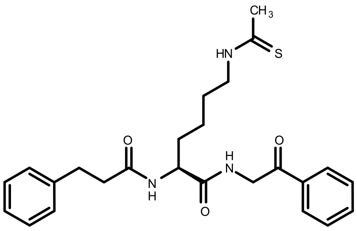

Thioacetylated_peptide_derivative_12.mol

23

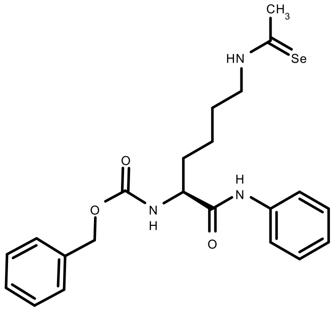

N_epsilon_Modified lysine_derivative 89.mol

24

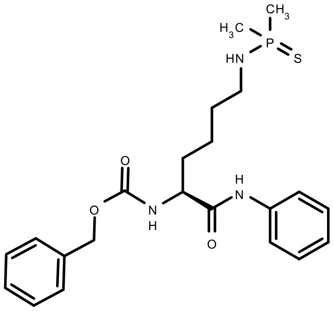

N_epsilon_Modified lysine_derivative 86.mol

25

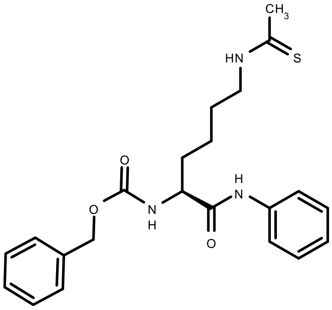

N_epsilon_Modified lysine_derivative 84.mol

26

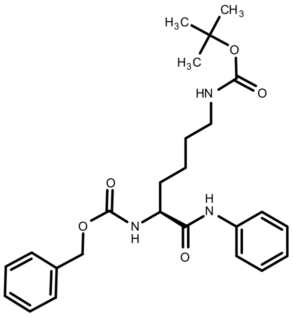

N_epsilon_Modified lysine_derivative 83.mol

27

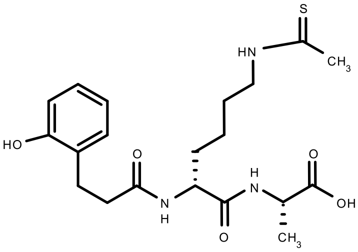

K_thioaceyl_A_derv_5.mol K_thioaceyl_A_derv_6.mol

28

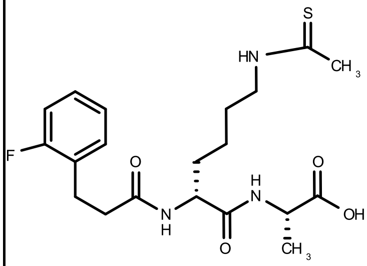

K_thioaceyl_A_derv_4.mol K_thioaceyl_A_derv_5.mol

29

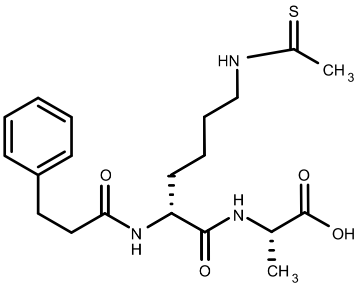

K_thioaceyl_A_derv_3.mol K_thioaceyl_A_derv_4.mol

30

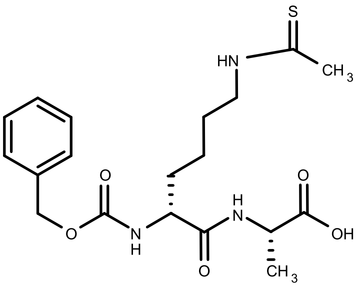

K_thioaceyl_A_derv_2.mol K_thioaceyl_A_derv_3.mol

31

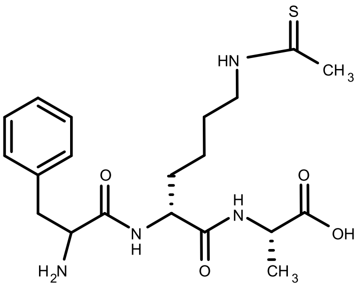

K_thioaceyl_A_derv_1.mol K_thioaceyl_A_derv_2.mol

32

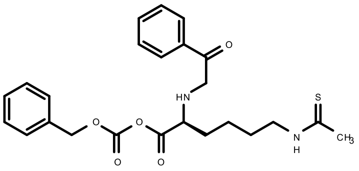

Cbz_K_thioaceyl_derv_4.mol Cbz_K_thioaceyl_derv_5.mol

Bin cluster size of 37

33

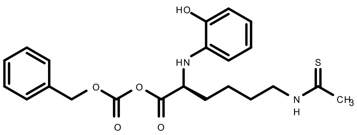

Cbz_K_thioaceyl_derv_3.mol Cbz_K_thioaceyl_derv_4.mol

34

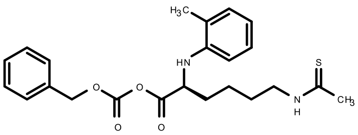

Cbz_K_thioaceyl_derv_2.mol Cbz_K_thioaceyl_derv_3.mol

35

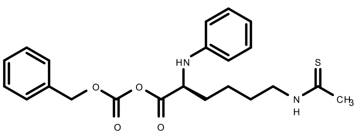

K_thioaceyl_A_derv_7.mol Cbz_K_thioaceyl_derv_1.mol

36

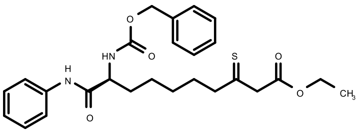

Acetyl_lysine_mimics1_5.mol

37

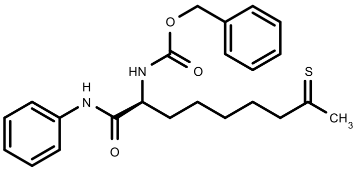

Acetyl_lysine_mimics1_4.mol

Bin cluster size of 39

1

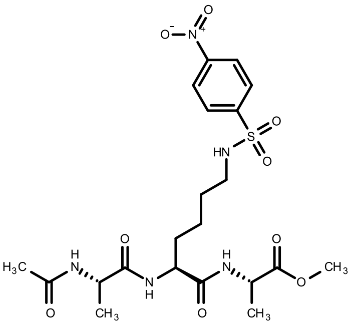

N_epsilon_Modified lysine_derivative 82.mol

2

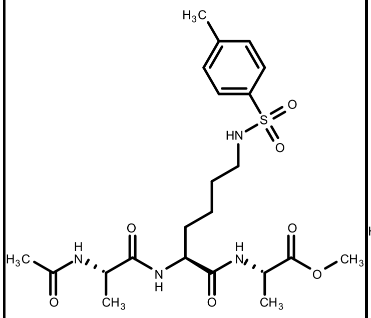

N_epsilon_Modified lysine_derivative 81.mol

3

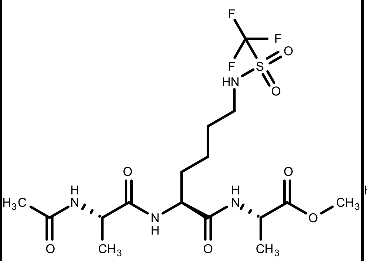

N_epsilon_Modified lysine_derivative 80.mol

4

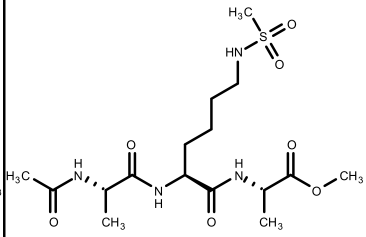

N_epsilon_Modified lysine_derivative 79.mol

5

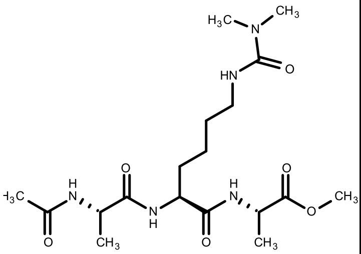

N_epsilon_Modified lysine_derivative 78.mol

6

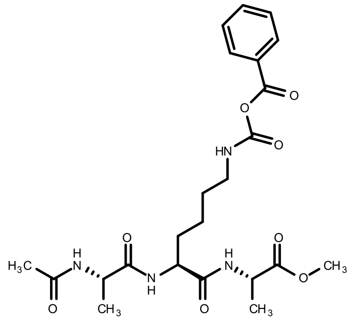

N_epsilon_Modified lysine_derivative 76.mol

7

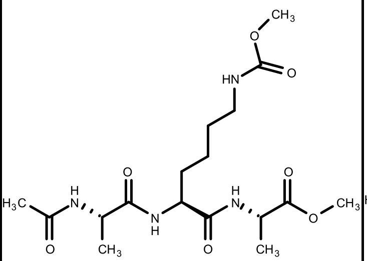

N_epsilon_Modified lysine_derivative 75.mol

8

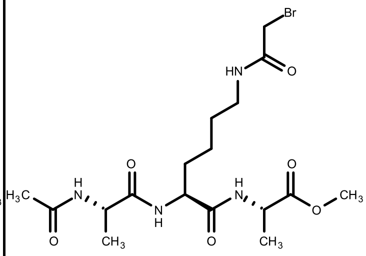

N_epsilon_Modified lysine_derivative 74.mol

9

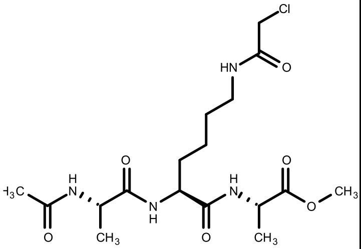

N_epsilon_Modified lysine_derivative 73.mol

10

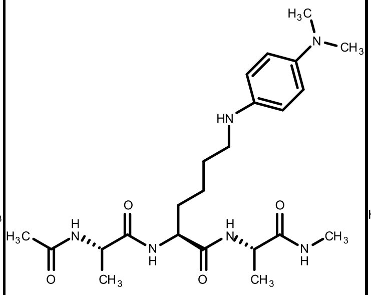

N_epsilon_Modified lysine_derivative 72.mol

11

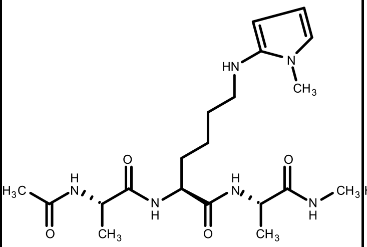

N_epsilon_Modified lysine_derivative 71.mol

12

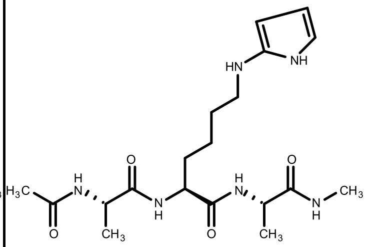

N_epsilon_Modified lysine_derivative 70.mol

13

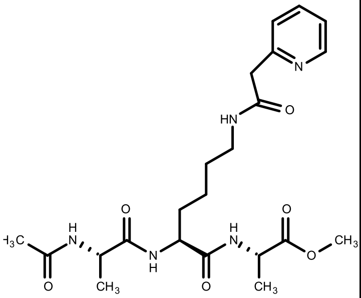

N_epsilon_Modified lysine_derivative 69.mol

14

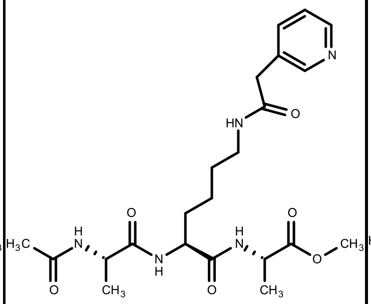

N_epsilon_Modified lysine_derivative 68.mol

15

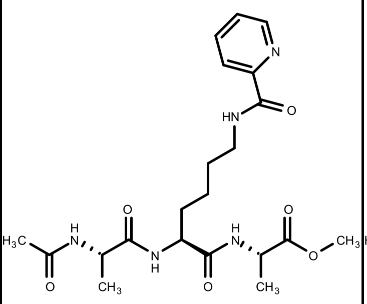

N_epsilon_Modified lysine_derivative 67.mol

16

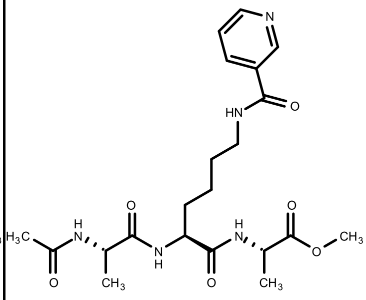

N_epsilon_Modified lysine_derivative 66.mol

Bin cluster size of 39

17

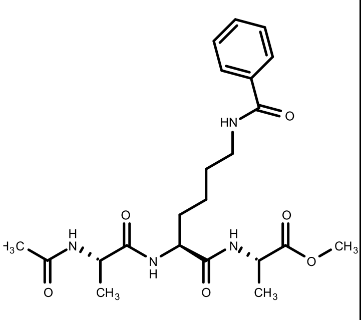

N_epsilon_Modified lysine_derivative 65.mol

18

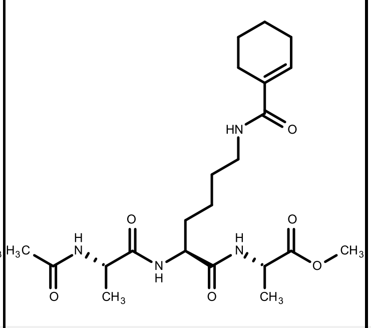

N_epsilon_Modified lysine_derivative 64.mol

19

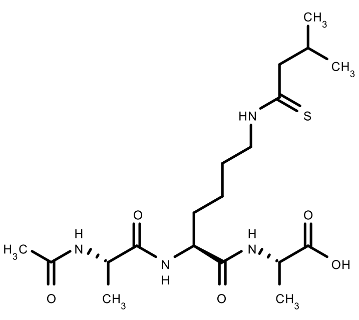

N_epsilon_Modified lysine_derivative 62.mol

20

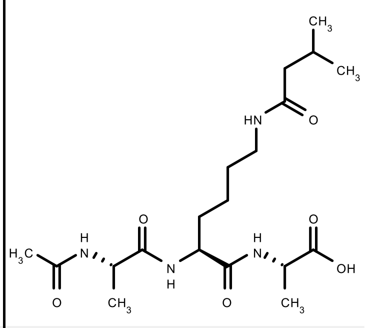

N_epsilon_Modified lysine_derivative 61.mol

21

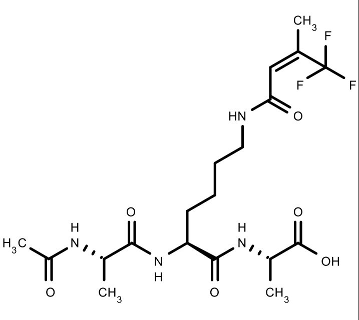

N_epsilon_Modified lysine_derivative 60.mol

22

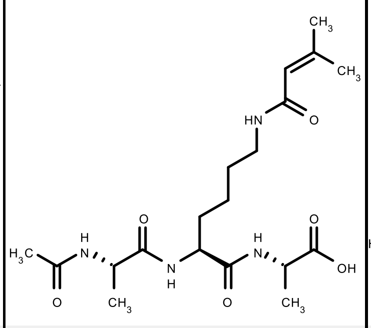

N_epsilon_Modified lysine_derivative 59.mol

23

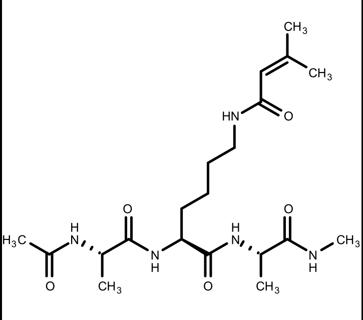

N_epsilon_Modified lysine_derivative 58.mol

24

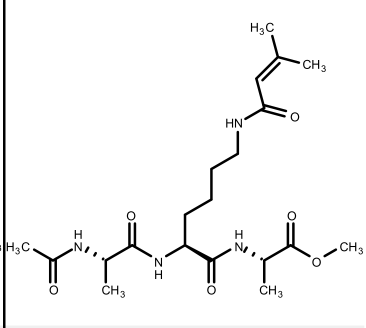

N_epsilon_Modified lysine_derivative 57.mol

25

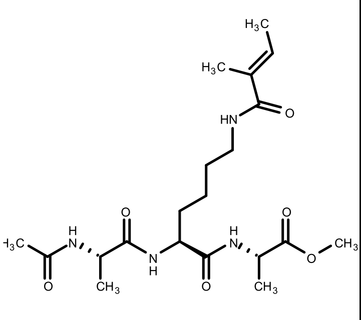

N_epsilon_Modified lysine_derivative 56.mol

26

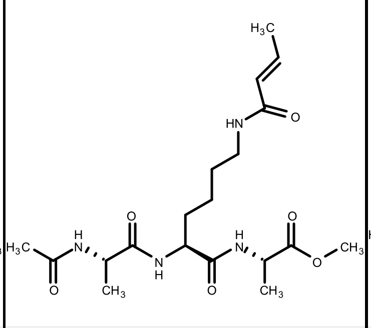

N_epsilon_Modified lysine_derivative 55.mol

27

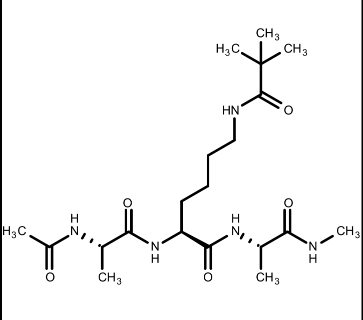

N_epsilon_Modified lysine_derivative 54.mol

28

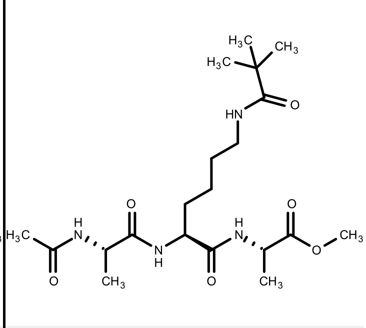

N_epsilon_Modified lysine_derivative 53.mol

29

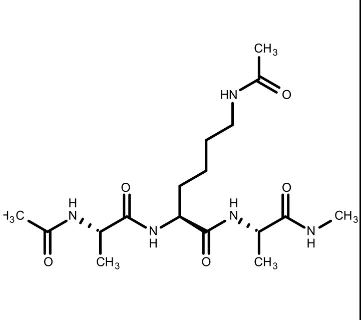

N_epsilon_Modified lysine_derivative 52.mol

30

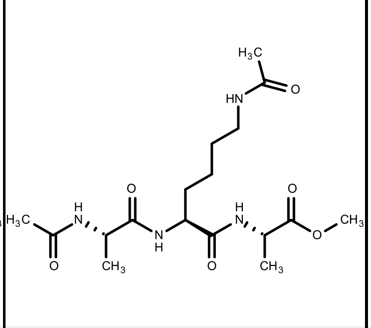

N_epsilon_Modified lysine_derivative 51.mol

31

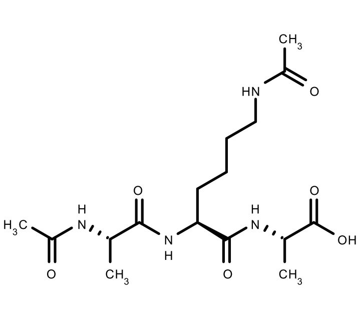

N_epsilon_Modified lysine_derivative 50.mol

32

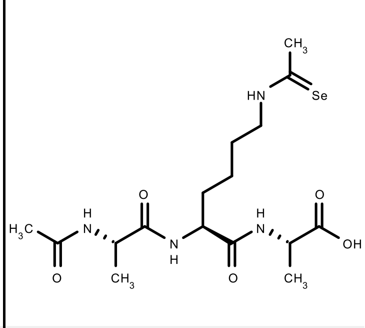

N_epsilon_Modified lysine_derivative 49.mol

Bin cluster size of 39

33	34	35	36
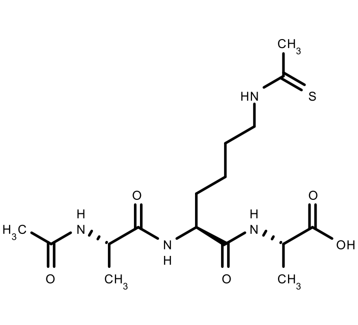	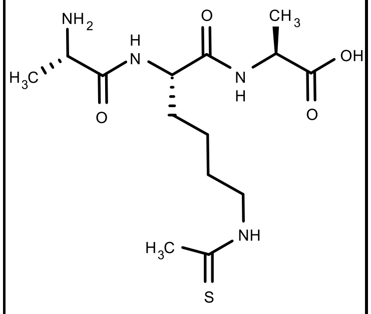	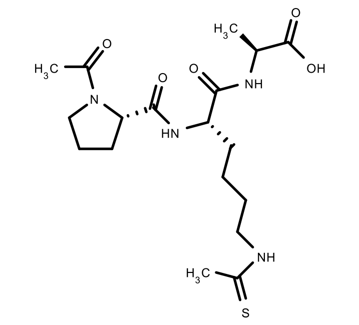	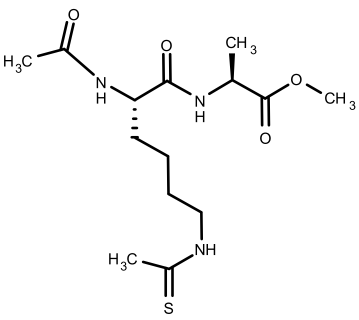
N_epsilon_Modified lysine_derivative 48.mol	AK_thioaceyl_A.mol	Ac_K(thioaceyl)A_OMe.mol Ac_PK_thioaceyl A.mol	K(thioaceyl)A.mol Ac_K_thioaceyl_A_OMe.mol
37	38	39	
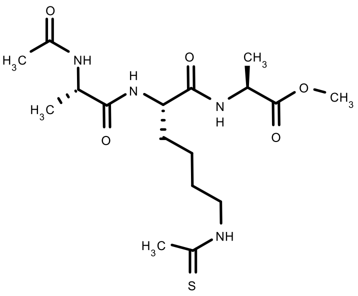	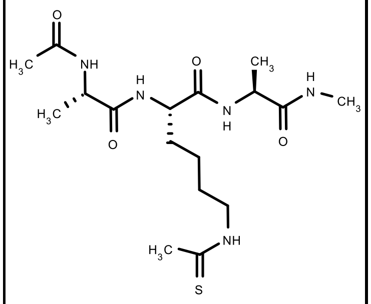	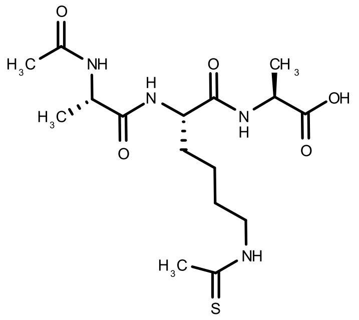	
Ac-AK(thioaceyl)A.mol Ac_AK_thioaceyl A OMe.mol	Ac-AK(thioaceyl)A.mol Ac_AK_thioaceyl A NHMe.mol	KK(thioaceyl)L.mol Ac_AK_thioaceyl_A.mol	

Bin cluster size of 59

1

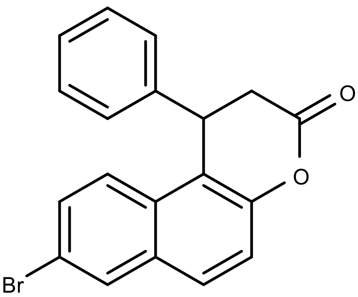

Thiobarbiturates_derivative1 24.mol

2

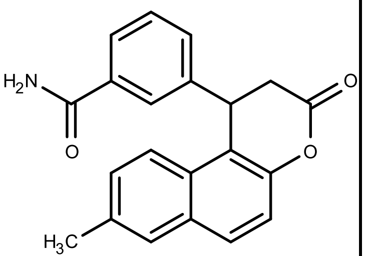

Thiobarbiturates_derivative1 23.mol

3

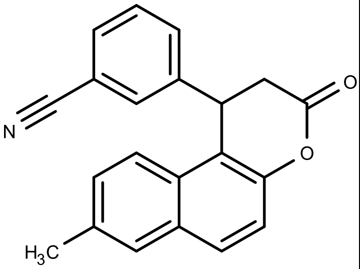

Thiobarbiturates_derivative1 22.mol

4

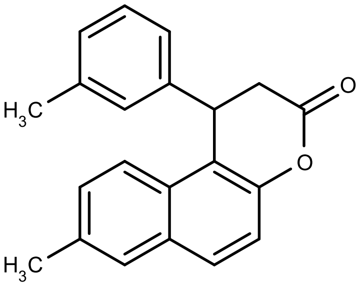

Thiobarbiturates_derivative1 21.mol

5

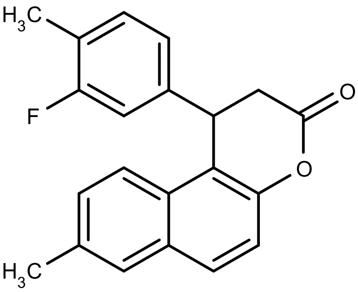

Thiobarbiturates_derivative1 20.mol

6

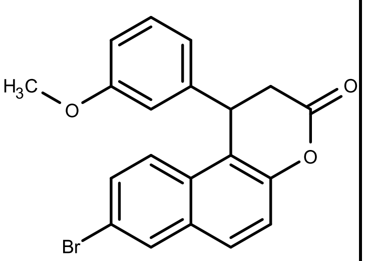

Thiobarbiturates_derivative1 19.mol

7

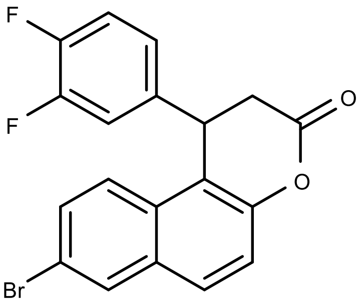

Thiobarbiturates_derivative1 18.mol

8

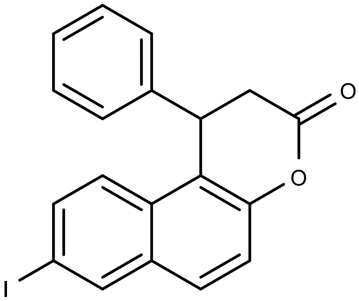

Thiobarbiturates_derivative1 17.mol

9

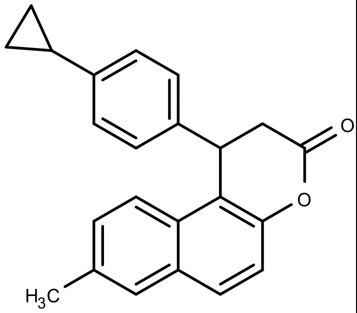

Thiobarbiturates_derivative1 16.mol

10

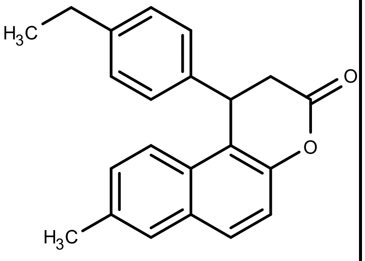

Thiobarbiturates_derivative1 15.mol

11

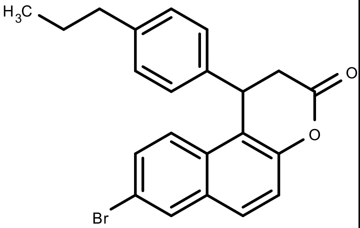

Thiobarbiturates_derivative1 14.mol

12

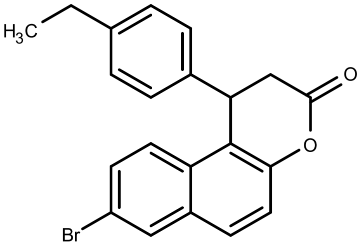

Thiobarbiturates_derivative1 13.mol

13

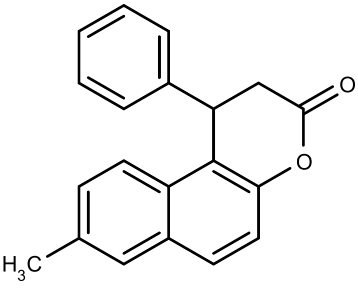

Thiobarbiturates_derivative1 12.mol

14

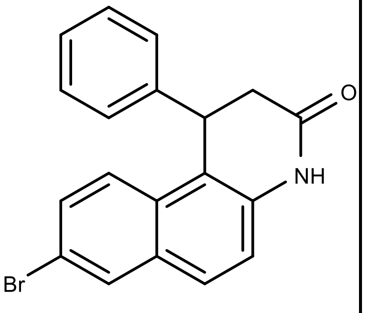

Thiobarbiturates_derivative1 6.mol

15

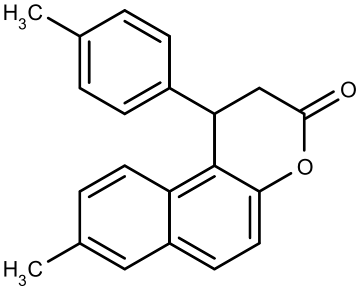

Thiobarbiturates_derivative1 2.mol

16

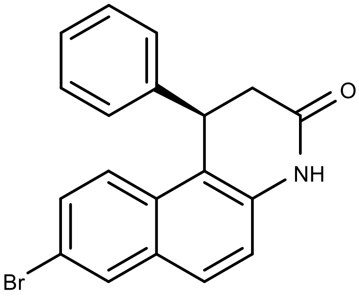

splitomicin_derv_52.mol

Bin cluster size of 59

17

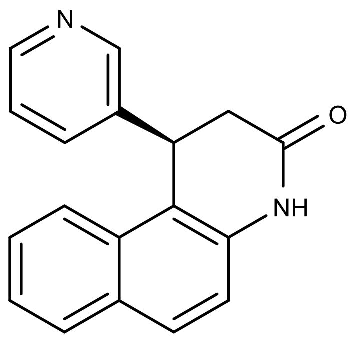

splitomicin_derv_50.mol

18

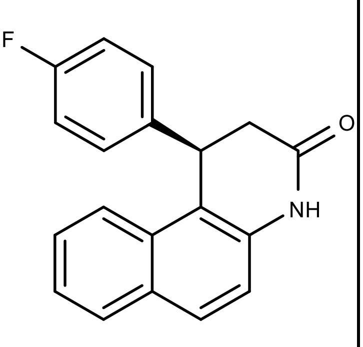

splitomicin_derv_49.mol

19

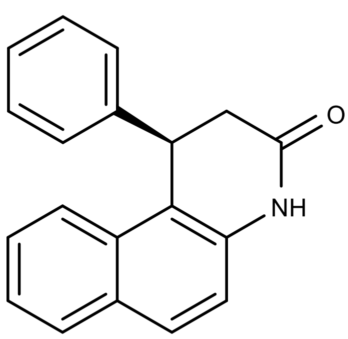

splitomicin_derv_48.mol

20

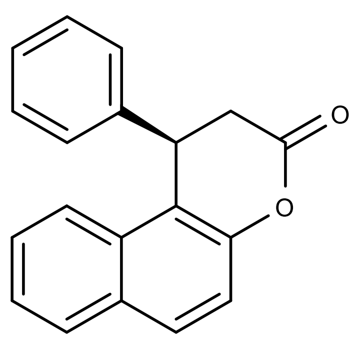

splitomicin_derv_47.mol

21

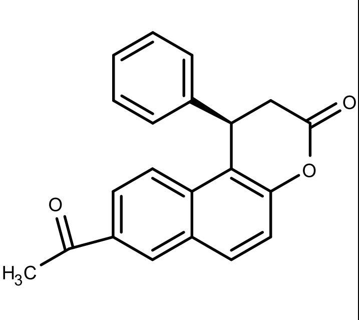

splitomicin_derv_46.mol

22

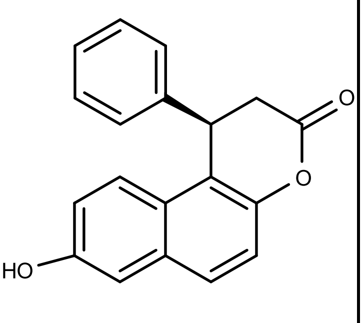

splitomicin_derv_45.mol

23

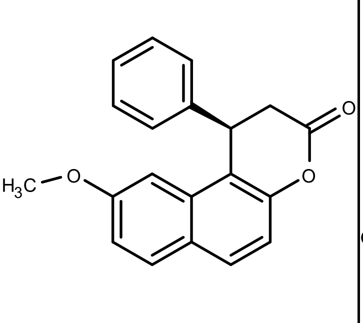

splitomicin_derv_44.mol

24

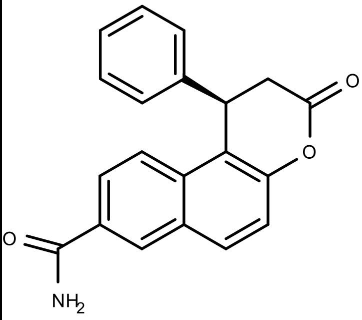

splitomicin_derv_38.mol

25

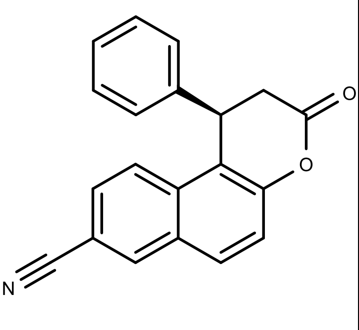

splitomicin_derv_35.mol

26

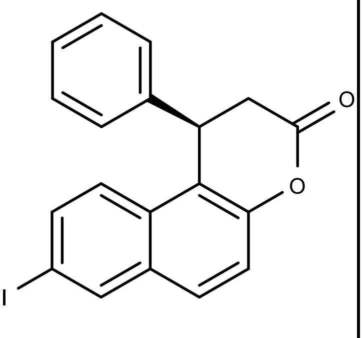

splitomicin_derv_34.mol

27

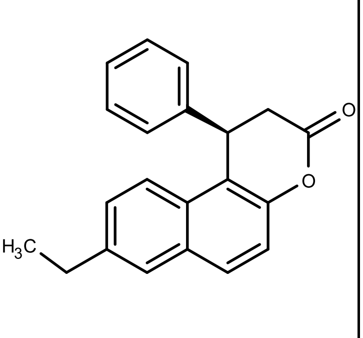

splitomicin_derv_33.mol

28

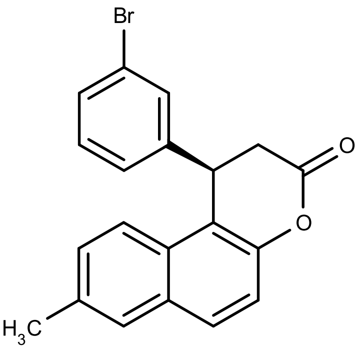

splitomicin_derv_32.mol

29

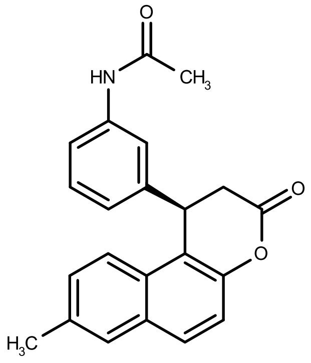

splitomicin_derv_31.mol

30

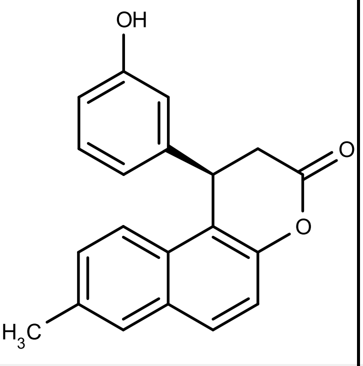

splitomicin_derv_29.mol

31

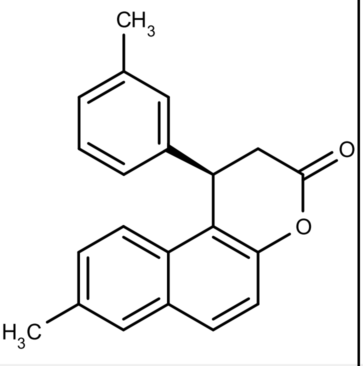

splitomicin_derv_28.mol

32

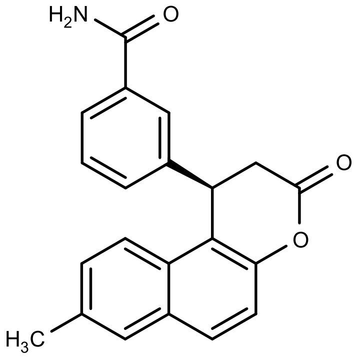

splitomicin_derv_27.mol

33

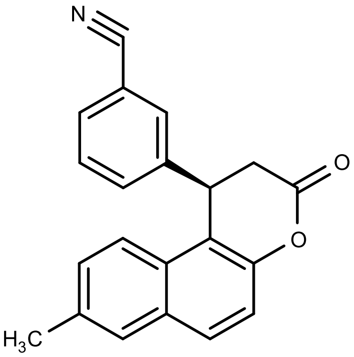

splitomicin_derv_26.mol

34

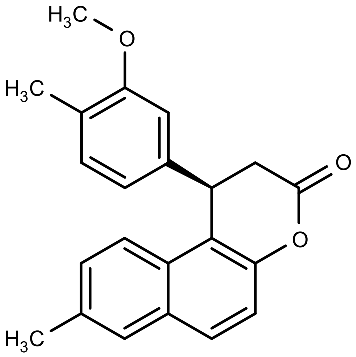

splitomicin_derv_25.mol

35

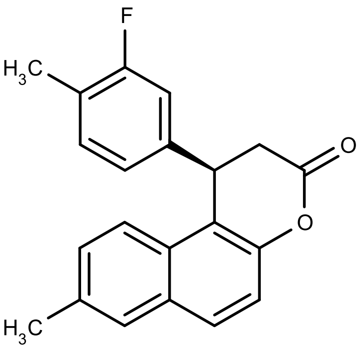

splitomicin_derv_24.mol

36

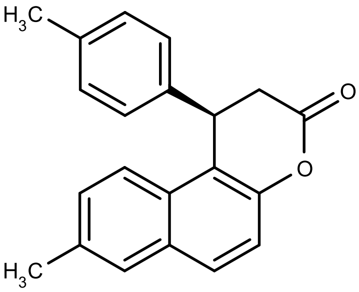

splitomicin_derv_23 S isomer.mol

37

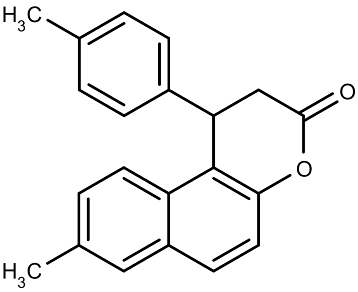

splitomicin_derv_23 R isomer.mol

38

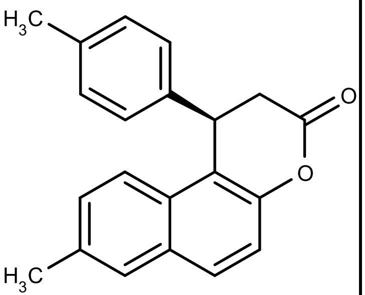

splitomicin_derv_22.mol

39

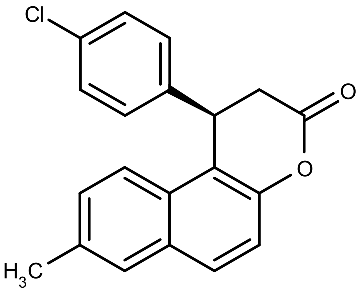

splitomicin_derv_21.mol

40

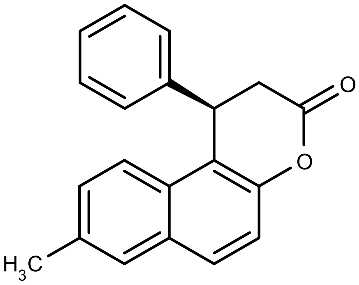

splitomicin_derv_20 S isomer.mol

41

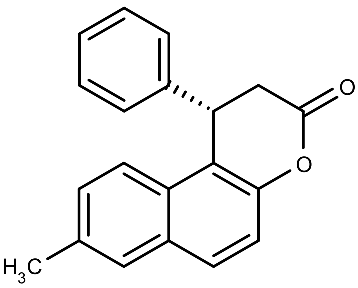

splitomicin_derv_20 R isomer.mol

42

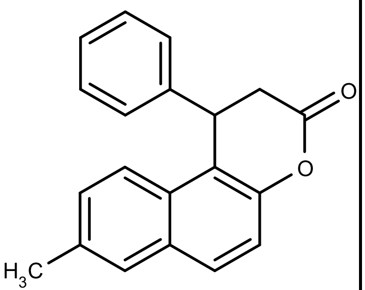

splitomicin_derv_19.mol

43

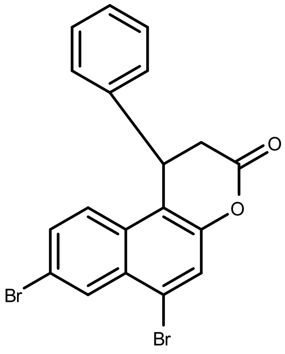

splitomicin_derv_18.mol

44

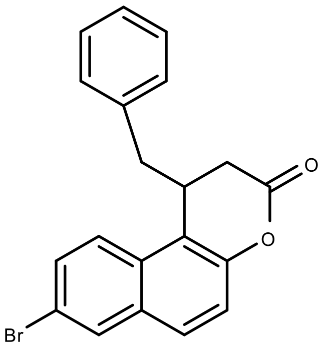

splitomicin_derv_17.mol

45

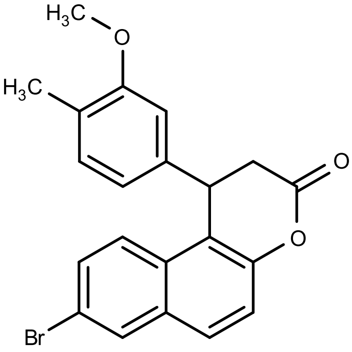

splitomicin_derv_15.mol

46

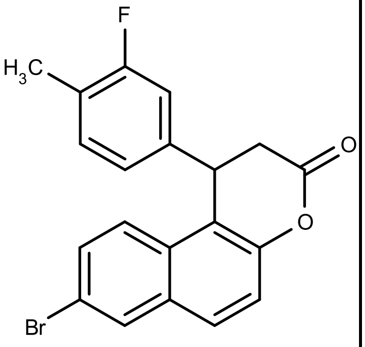

splitomicin_derv_14.mol

47

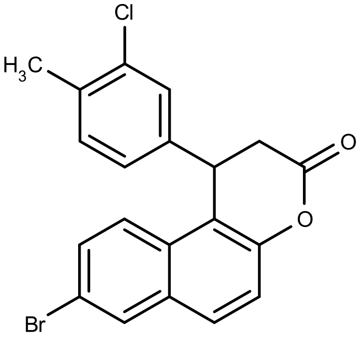

splitomicin_derv_13.mol

48

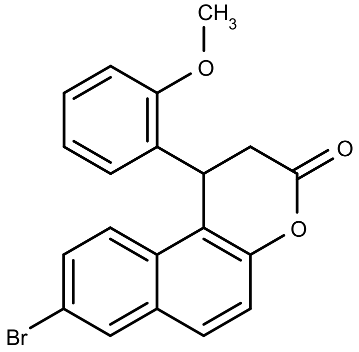

splitomicin_derv_12.mol

49

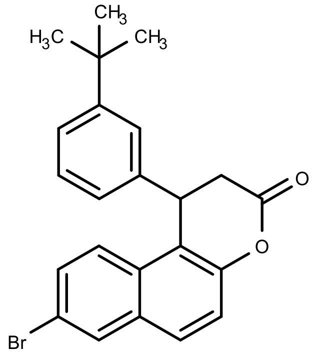

splitomicin_derv_1 1.mol

50

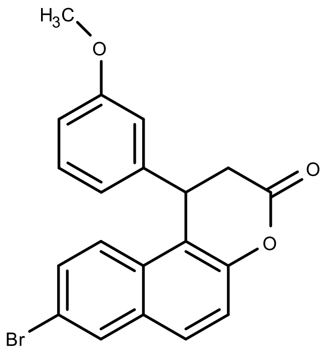

splitomicin_derv_1 0.mol

51

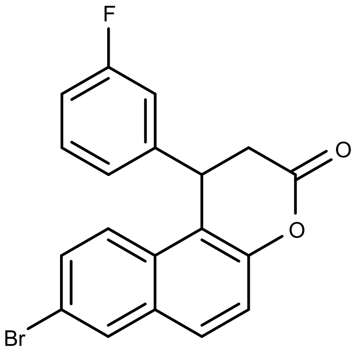

splitomicin_derv_8 .mol splitomicin d

52

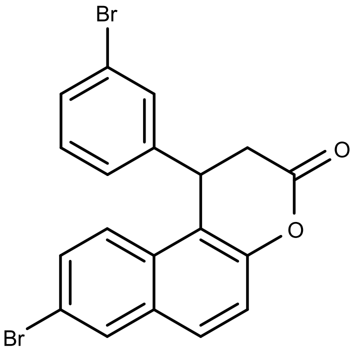

splitomicin_derv_8 .mol

53

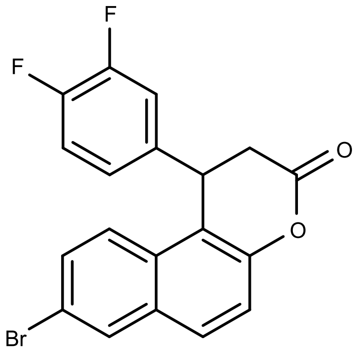

splitomicin_derv_7 .mol

54

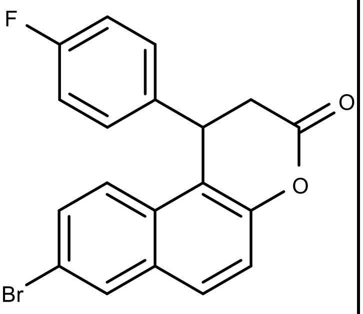

splitomicin_derv_6 .mol

55

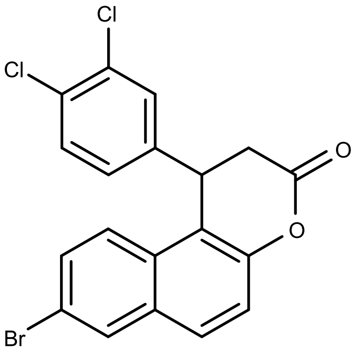

splitomicin_derv_4 .mol splitomicin d

56

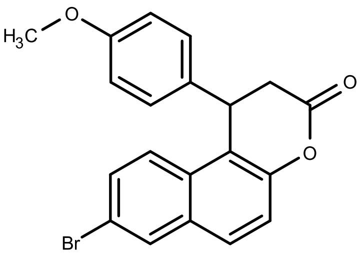

splitomicin_derv_3 .mol splitomicin d

57

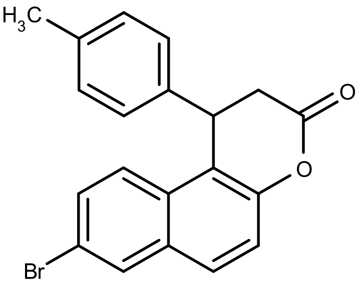

splitomicin_derv_2 .mol splitomicin d

58

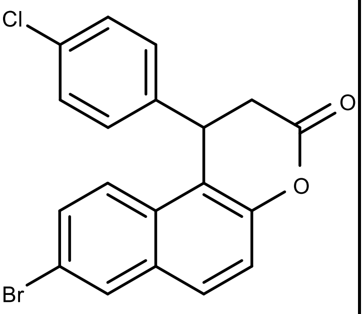

splitomicin_derv_1 .mol splitomicin d

59

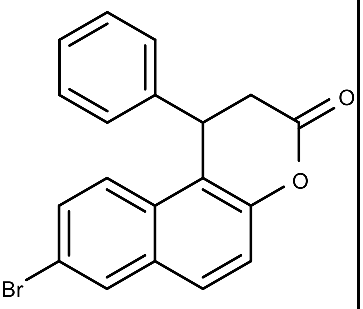

Untitled Document- 1 splitomicin derv

Bin cluster size of 82

1	2	3	4
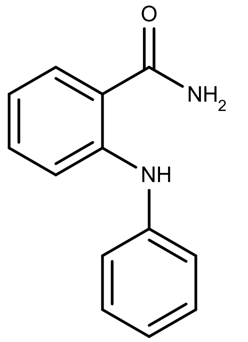	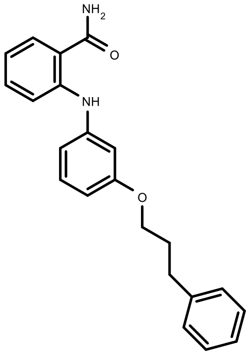	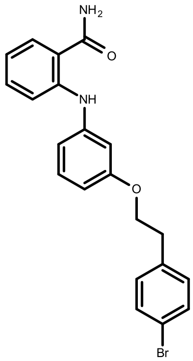	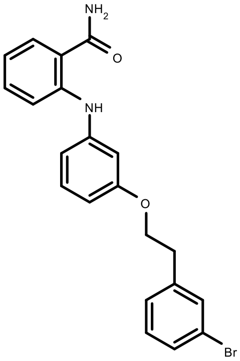
Anilinobenzamides_derivative 7a.mol	Anilinobenzamide_derivative1 34.mol	Anilinobenzamide_derivative1 33p.mol	Anilinobenzamide_derivative1 33o.mol
**5**	**6**	**7**	**8**
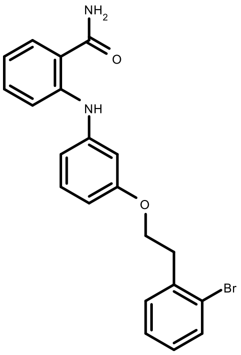	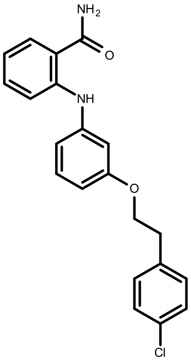	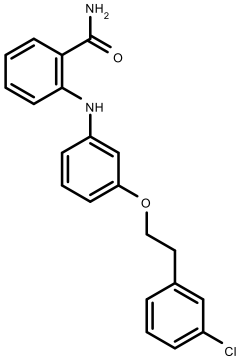	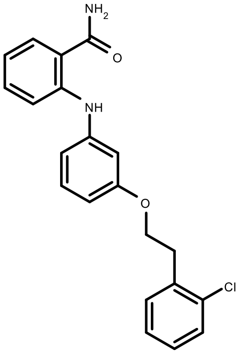
Anilinobenzamide_derivative1 33n.mol	Anilinobenzamide_derivative1 33m.mol	Anilinobenzamide_derivative1 33l.mol	Anilinobenzamide_derivative1 33k.mol
**9**	**10**	**11**	**12**
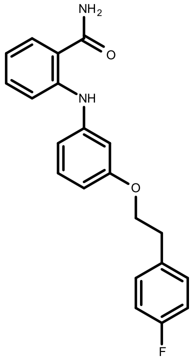	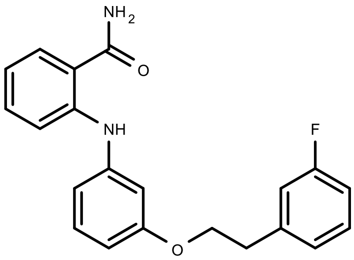	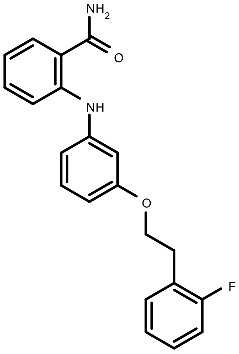	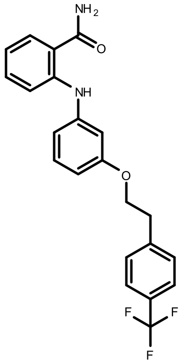
Anilinobenzamide_derivative1 33j.mol	Anilinobenzamide_derivative1 33i.mol	Anilinobenzamide_derivative1 33h.mol	Anilinobenzamide_derivative1 33g.mol
**13**	**14**	**15**	**16**
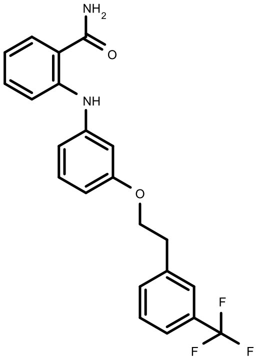	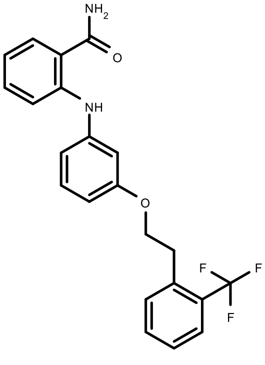	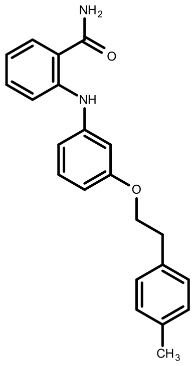	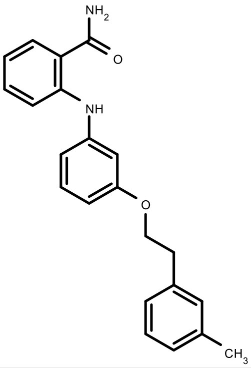
Anilinobenzamide_derivative1 33f.mol	Anilinobenzamide_derivative1 33e.mol	Anilinobenzamide_derivative1 33d.mol	Anilinobenzamide_derivative1 33c.mol

Bin cluster size of 82

17

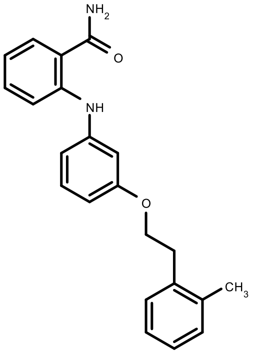

Anilinobenzamide_derivative1 33b.mol

18

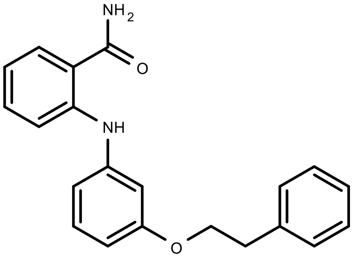

Anilinobenzamide_derivative1 33a.mol

19

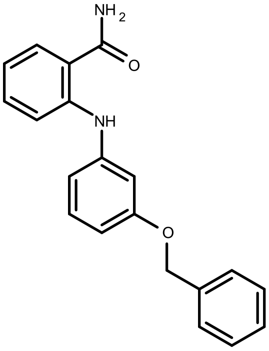

Anilinobenzamide_derivative1 32.mol

20

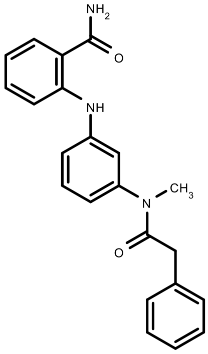

Anilinobenzamide_derivative1 29.mol

21

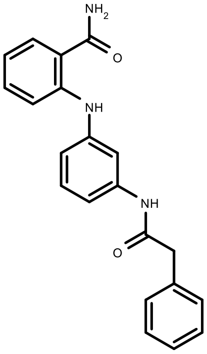

Anilinobenzamide_derivative1 28.mol

22

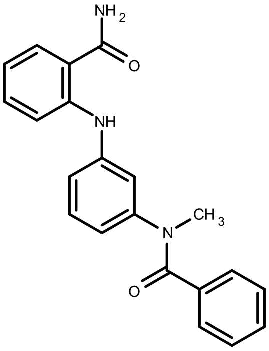

Anilinobenzamide_derivative1 27.mol

23

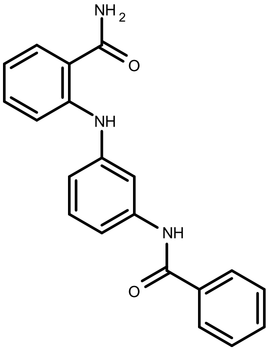

Anilinobenzamide_derivative1 26.mol

24

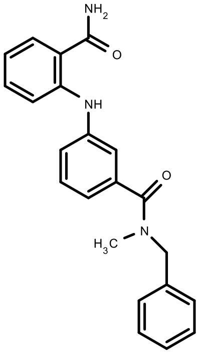

Anilinobenzamide_derivative1 25.mol

25

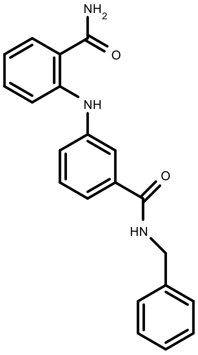

Anilinobenzamide_derivative1 24.mol

26

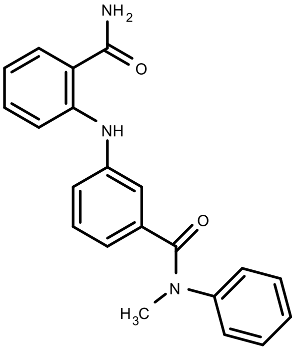

Anilinobenzamide_derivative1 23.mol

27

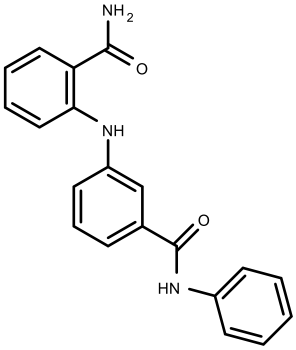

Anilinobenzamide_derivative1 22.mol

28

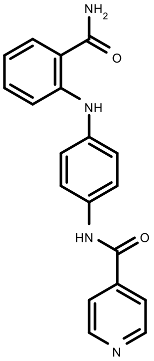

Anilinobenzamide_derivative1 21.mol

29

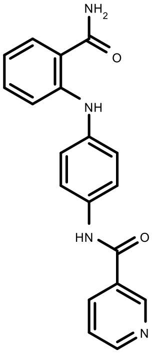

Anilinobenzamide_derivative1 20.mol

30

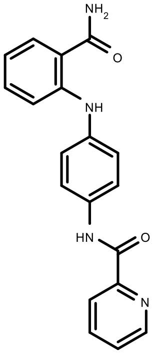

Anilinobenzamide_derivative1 19.mol

31

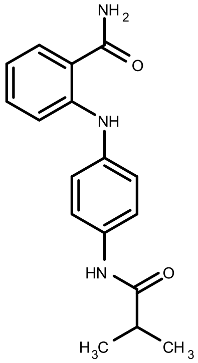

Anilinobenzamide_derivative1 16.mol

32

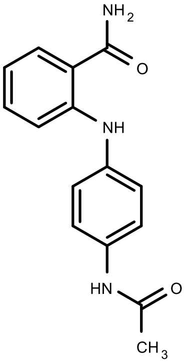

Anilinobenzamide_derivative1 15.mol

Bin cluster size of 82

33	34	35	36
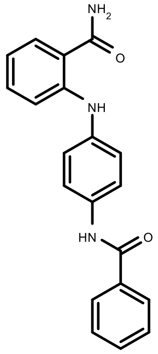	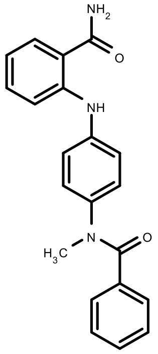	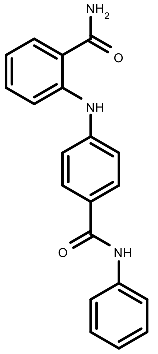	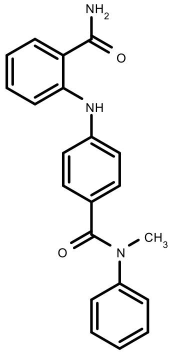
Anilinobenzamide_derivative1 14.mol	Anilinobenzamide_derivative1 13.mol	Anilinobenzamide_derivative1 12.mol	Anilinobenzamide_derivative1 11.mol
**37**	**38**	**39**	**40**
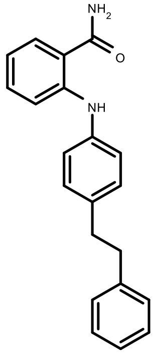	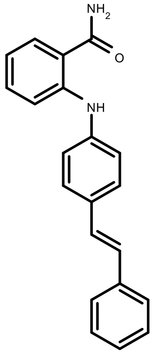	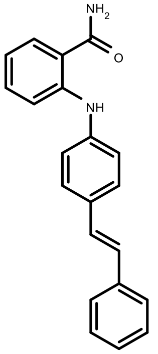	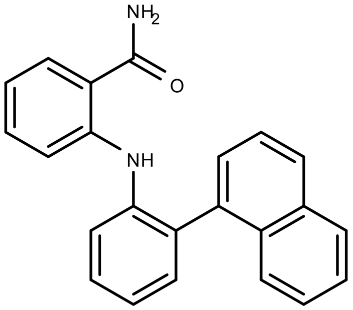
Anilinobenzamide_derivative1 10.mol	Anilinobenzamide_derivative1 9.mol	Anilinobenzamide_derivative1 8.mol	Anilinobenzamide_derivative1 7z.mol
**41**	**42**	**43**	**44**
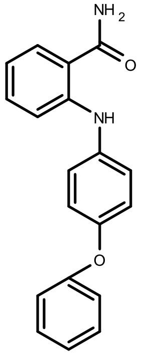	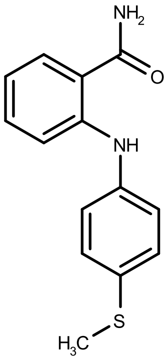	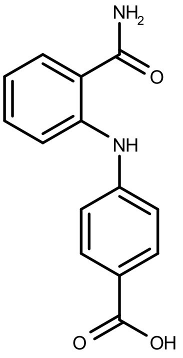	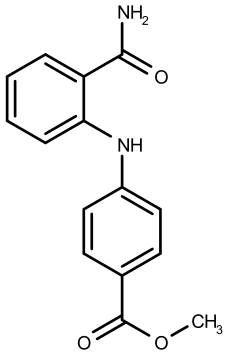
Anilinobenzamide_derivative1 7y.mol	Anilinobenzamide_derivative1 7x.mol	Anilinobenzamide_derivative1 7w.mol	Anilinobenzamide_derivative1 7v.mol
**45**	**46**	**47**	**48**
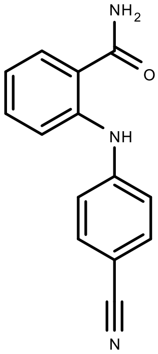	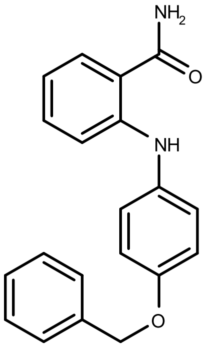	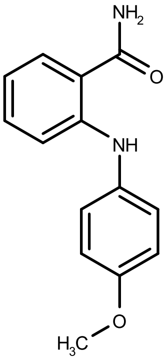	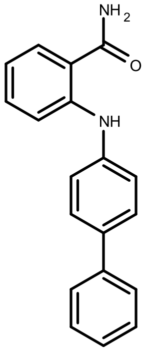
Anilinobenzamide_derivative1 7u.mol	Anilinobenzamide_derivative1 7t.mol	Anilinobenzamide_derivative1 7s.mol	Anilinobenzamide_derivative1 7r.mol

Bin cluster size of 82

49	50	51	52
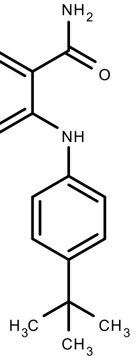 Anilinobenzamide_derivative1 7q.mol	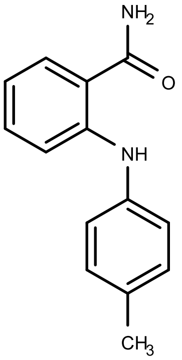 Anilinobenzamide_derivative1 7o.mol	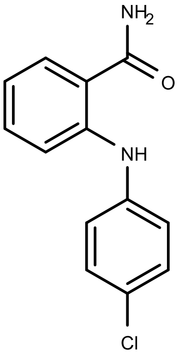 Anilinobenzamide_derivative1 7n.mol	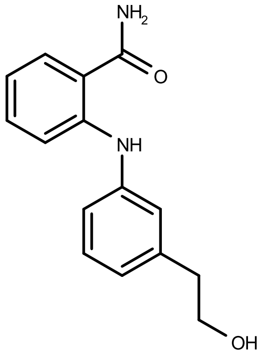 Anilinobenzamide_derivative1 7m.mol
**53**	**54**	**55**	**56**
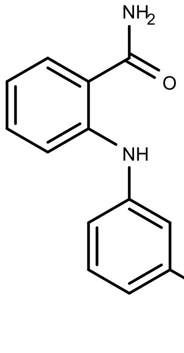 Anilinobenzamide_derivative1 7l.mol	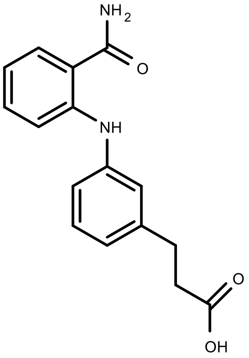 Anilinobenzamide_derivative1 7k.mol	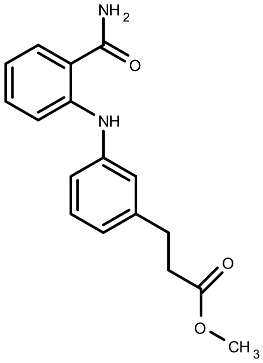 Anilinobenzamide_derivative1 7j.mol	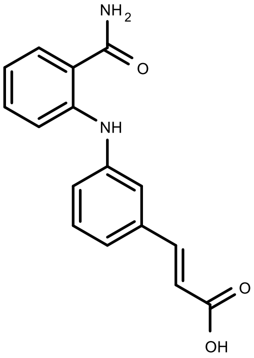 Anilinobenzamide_derivative1 7i.mol
**57**	**58**	**59**	**60**
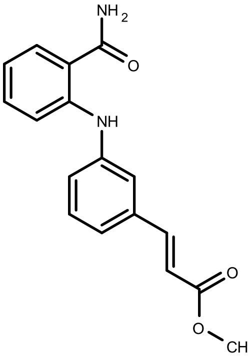 Anilinobenzamide_derivative1 7h.mol	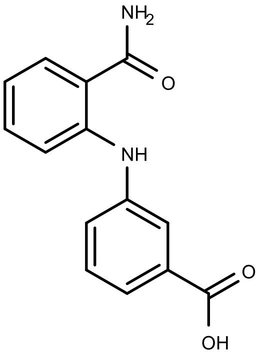 Anilinobenzamide_derivative1 7g.mol	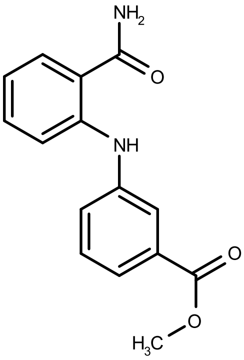 Anilinobenzamide_derivative1 7f.mol	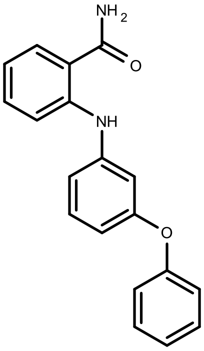 Anilinobenzamide_derivative1 7e.mol
**61**	**62**	**63**	**64**
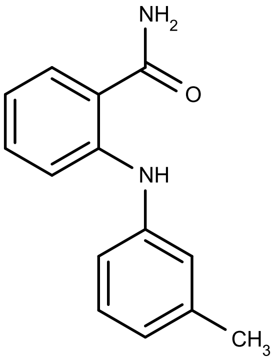 Anilinobenzamide_derivative1 7c.mol	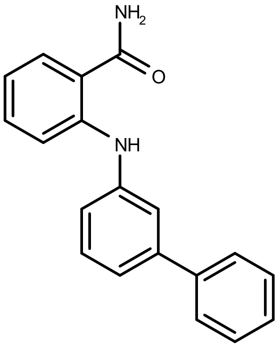 Anilinobenzamide_derivative1 7b.mol	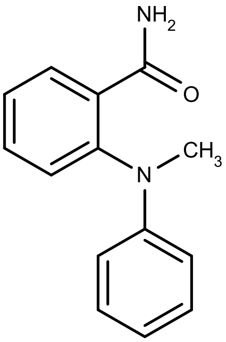 Anilinobenzamide_derivative1 7ag.mol	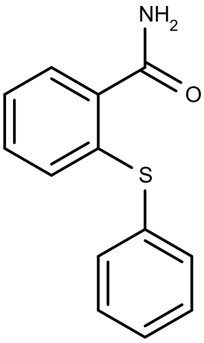 Anilinobenzamide_derivative1 7af.mol

65

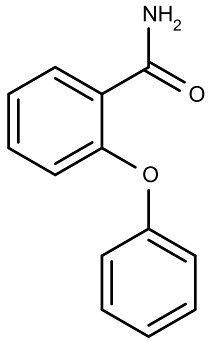

Anilinobenzamide_derivative1 7ae.mol

66

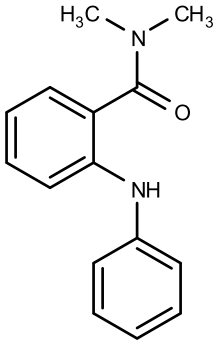

Anilinobenzamide_derivative1 7ad.mol

67

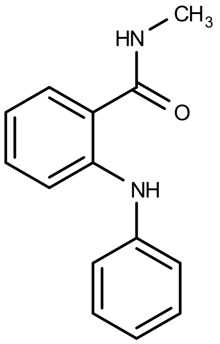

Anilinobenzamide_derivative1 7ac.mol

68

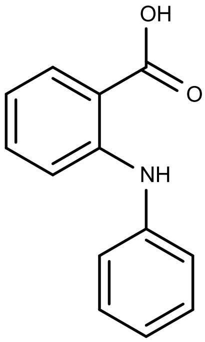

Anilinobenzamide_derivative1 7ab.mol

69

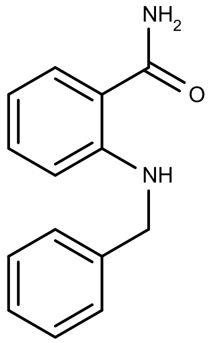

Anilinobenzamide_derivative1 7aa.mol

70

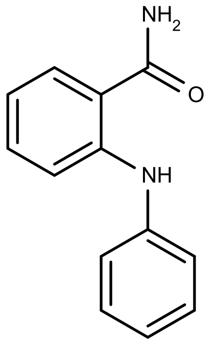

Anilinobenzamide_derivative1 7a.mol

71

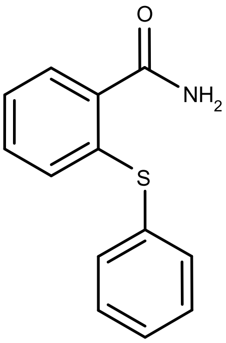

Anilinobenzamide_derivative 12.mol

72

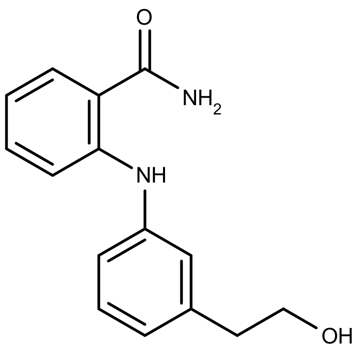

Anilinobenzamide_derivative 9.mol

73

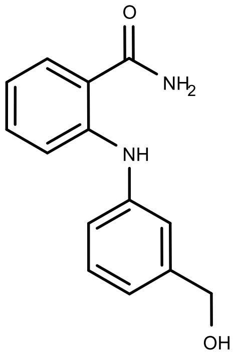

Anilinobenzamide_derivative 8.mol

74

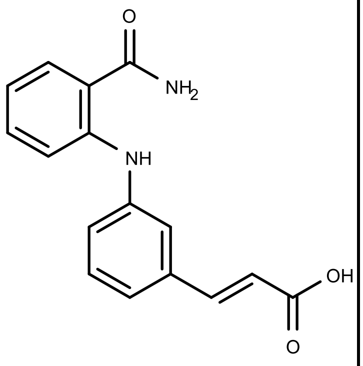

Anilinobenzamide_derivative 7.mol

75

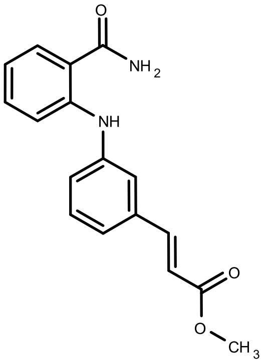

Anilinobenzamide_derivative 6.mol

76

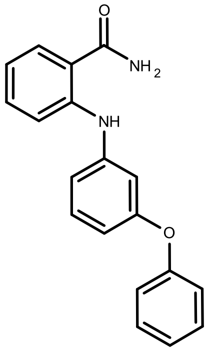

Anilinobenzamide_derivative 5.mol

77

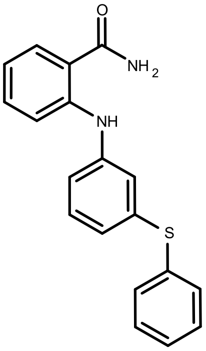

Anilinobenzamide_derivative 4.mol

78

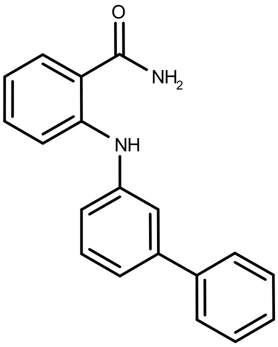

Anilinobenzamide_derivative 3.mol

79

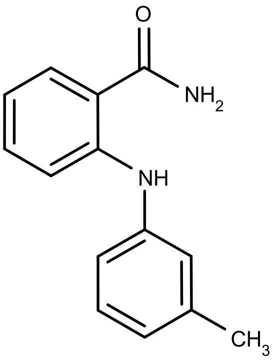

Anilinobenzamide_derivative 2.mol

80

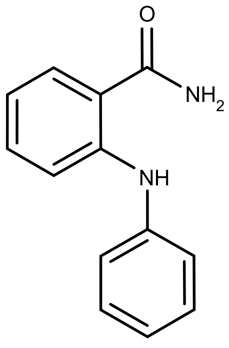

Anilinobenzamide_derivative 1.mol

Bin cluster size of 82

81	82		
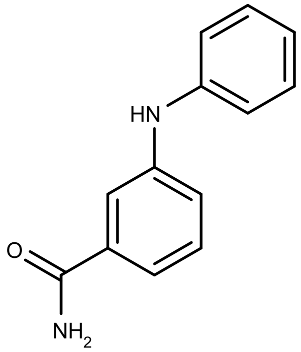 68509298 3_anilinobenzamide.mol	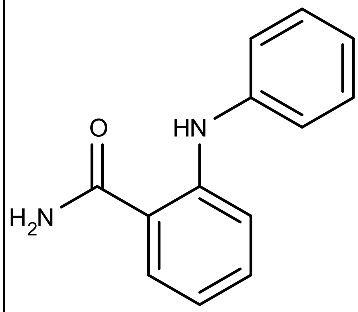 96455 2_anilinobenzamide.mol		

Bin cluster size of 83

1	2	3	4
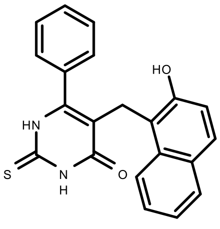	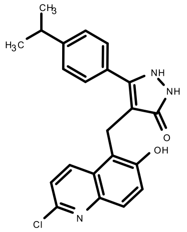	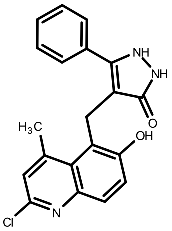	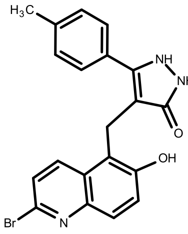
Thiobarbiturates_derivative1_3.mol	Quinolyl_pyrazolone_derivative_22.mol Quinolyl_pyrazolone_derivative_23.mol	Quinolyl_pyrazolone_derivative_21.mol Quinolyl_pyrazolone_derivative_22.mol	Quinolyl_pyrazolone_derivative_20.mol Quinolyl_pyrazolone_derivative_21.mol

5	6	7	8
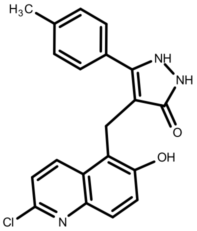	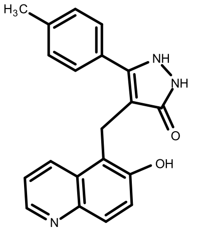	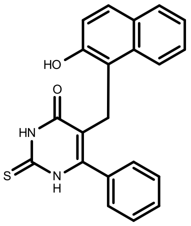	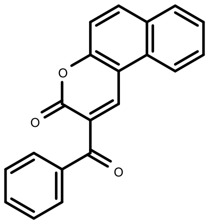
Quinolyl_pyrazolone_derivative_19.mol Quinolyl_pyrazolone_derivative_20.mol	Pyrazolone_derivative_18.mol Quinolyl_pyrazolone_derivative_19.mol	Pyrimidinediones_derivative1_cambinol.mol	Pyrimidinediones_derivative1_10.mol

9	10	11	12
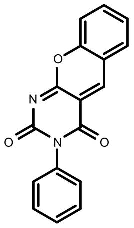	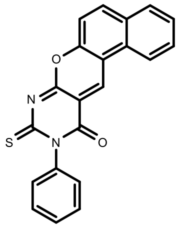	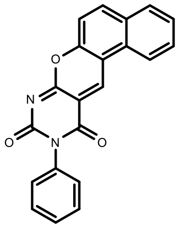	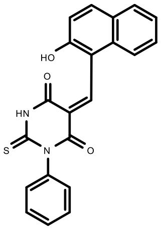
Pyrimidinediones_derivative1_8.mol	Pyrimidinediones_derivative1_6.mol	Pyrimidinediones_derivative1_5.mol	Pyrimidinediones_derivative1_4.mol

13	14	15	16
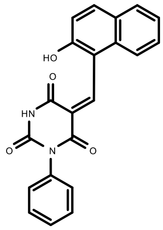	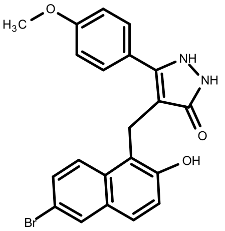	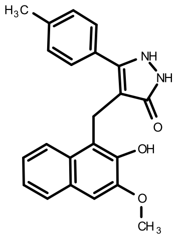	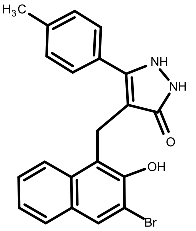
Pyrimidinediones_derivative1_3.mol	Pyrazolone_derivative_17.mol Pyrazolone_derivative_18.mol	Pyrazolone_derivative_16.mol Pyrazolone_derivative_17.mol	Pyrazolone_derivative_15.mol Pyrazolone_derivative_16.mol

Bin cluster size of 83

17	18	19	20
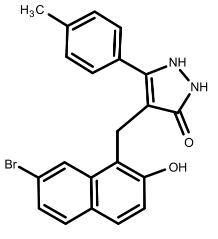	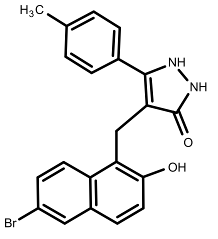	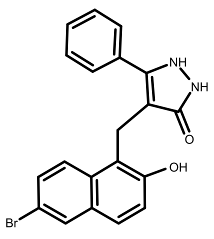	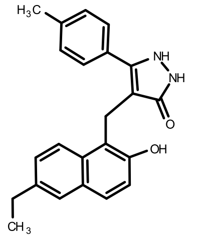
Pyrazolone_derivative_14.mol Pyrazolone_derivative_15.mol	Pyrazolone_derivative_13.mol Pyrazolone_derivative_14.mol	Pyrazolone_derivative_12.mol Pyrazolone_derivative_13.mol	Pyrazolone_derivative_11.mol Pyrazolone_derivative_12.mol
**21**	**22**	**23**	**24**
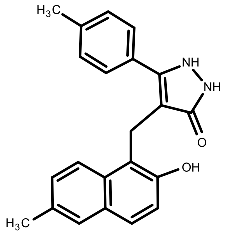	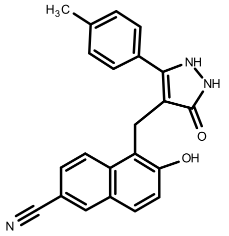	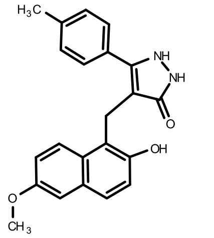	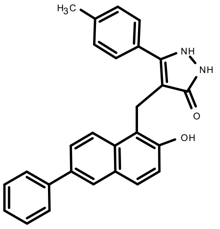
Pyrazolone_derivative_9.mol Pyrazolone_derivative_11.mol	Pyrazolone_derivative_9.mol Pyrazolone_derivative_10.mol	Pyrazolone_derivative_9.mol Pyrazolone_derivative_9.mol	Pyrazolone_derivative_8.mol Pyrazolone_derivative_8.mol
**25**	**26**	**27**	**28**
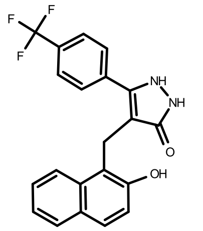	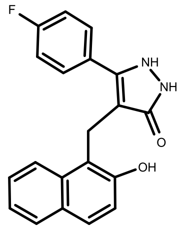	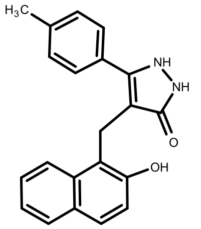	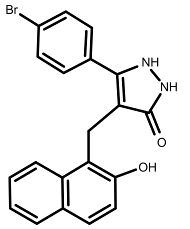
Pyrazolone_derivative_7.mol Pyrazolone_derivative_7.mol	Pyrazolone_derivative_6.mol Pyrazolone_derivative_6.mol	Pyrazolone_derivative_5.mol Pyrazolone_derivative_5.mol	Pyrazolone_derivative_4.mol Pyrazolone_derivative_4.mol
**29**	**30**	**31**	**32**
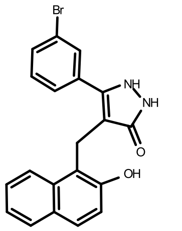	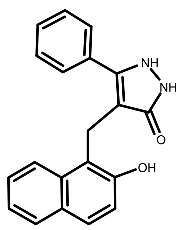	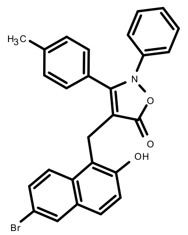	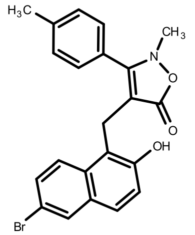
Pyrazolone_derivative_3.mol Pyrazolone_derivative_3.mol	Pyrazolone_derivative_2.mol Pyrazolone_derivative_2.mol	Napthyl_isoxazolone_derivative_25.mol Napthyl_isoxazolone_derivative_26.mol	Napthyl_isoxazolone_derivative_24.mol Napthyl_isoxazolone_derivative_25.mol

Bin cluster size of 83

33

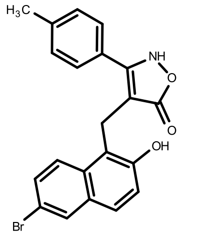

Quinolyl_pyrazolone_derivative_23.mol Napthyl_isoxazolone_derivative_24.mol

34

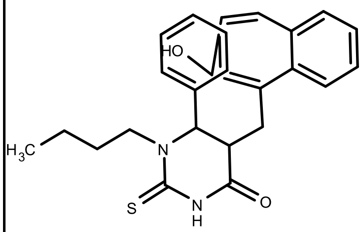

medda_cmpd_6j.mol

35

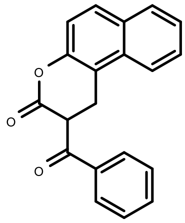

MC2141_analogue1_5t.mol

36

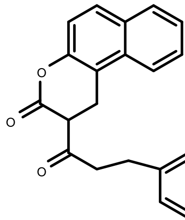

MC2141_analogue1_5s.mol

37

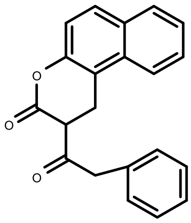

MC2141_analogue1_5r.mol

38

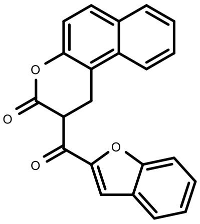

MC2141_analogue1_5q.mol

39

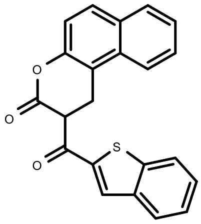

MC2141_analogue1_5p.mol

40

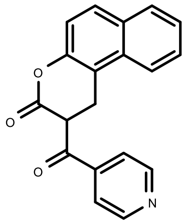

MC2141_analogue1_5o.mol

41

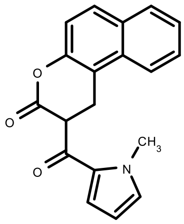

MC2141_analogue1_5n.mol

42

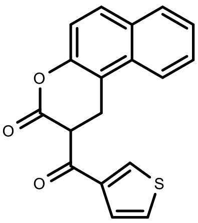

MC2141_analogue1_5m.mol

43

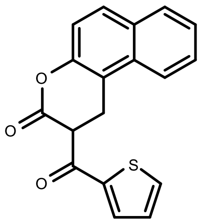

MC2141_analogue1_5l.mol

44

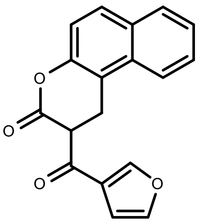

MC2141_analogue1_5k.mol

45

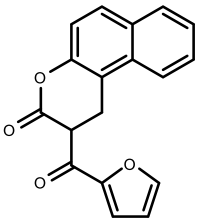

MC2141_analogue1_5j.mol

46

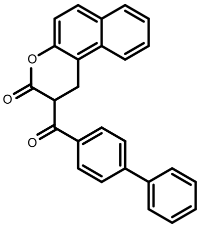

MC2141_analogue1_5i.mol

47

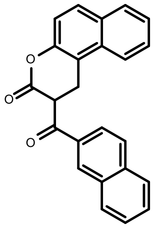

MC2141_analogue1_5h.mol

48

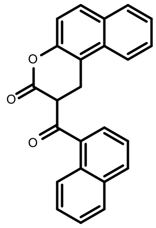

MC2141_analogue1_5g.mol

49

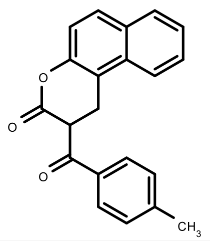

MC2141_analogue1_5f.mol

50

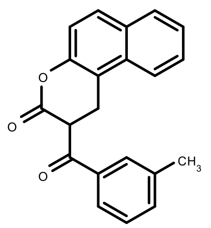

MC2141_analogue1_5e.mol

51

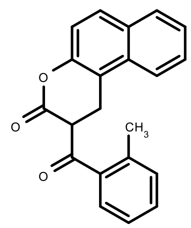

MC2141_analogue1_5d.mol

52

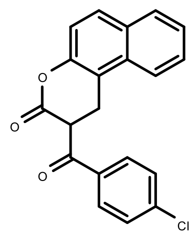

MC2141_analogue1_5c.mol

53

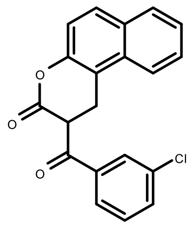

MC2141_analogue1_5b.mol

54

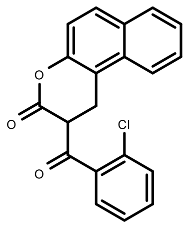

MC2141_analogue1_5a.mol

55

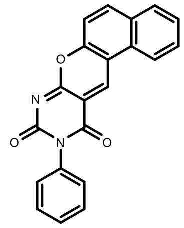

MC2141.mol

56

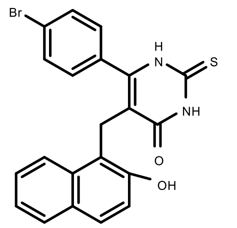

Cambinol_derivative1_13d.mol

57

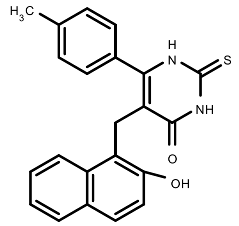

Cambinol_derivative1_13c.mol

58

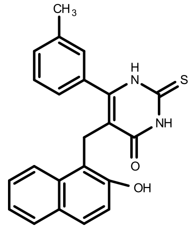

Cambinol_derivative1_13b.mol

59

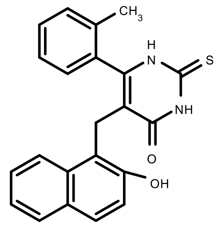

Cambinol_derivative1_13a.mol

60

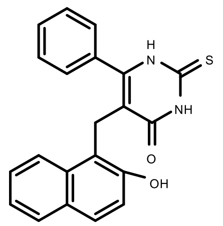

Cambinol_derivative1_12.mol

61

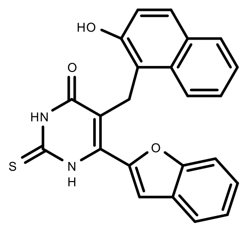

Cambinol_analogue1_6q.mol

62

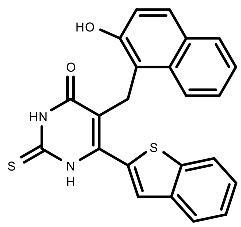

Cambinol_analogue1_6p.mol

63

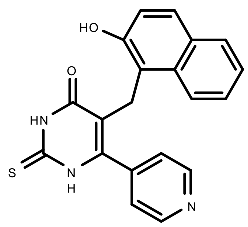

Cambinol_analogue1_6o.mol

64

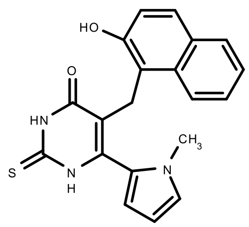

Cambinol_analogue1_6n.mol

65

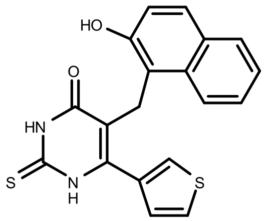

Cambinol_analogue1_6m.mol

66

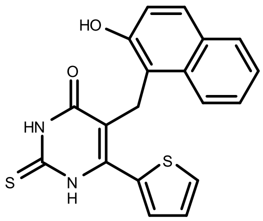

Cambinol_analogue1_6l.mol

67

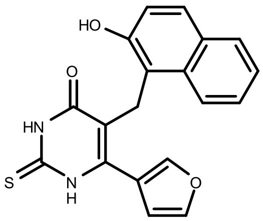

Cambinol_analogue1_6k.mol

68

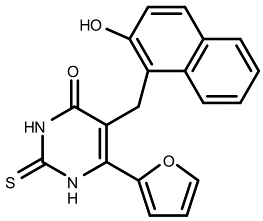

Cambinol_analogue1_6j.mol

69

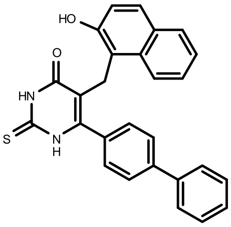

Cambinol_analogue1_6i.mol

70

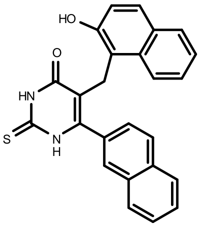

Cambinol_analogue1_6h.mol

71

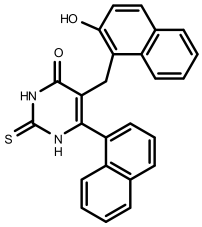

Cambinol_analogue1_6g.mol

72

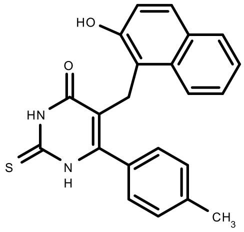

Cambinol_analogue1_6f.mol

73

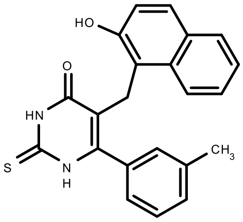

Cambinol_analogue1_6e.mol

74

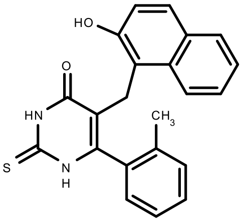

Cambinol_analogue1_6d.mol

75

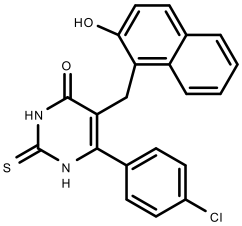

Cambinol_analogue1_6c.mol

76

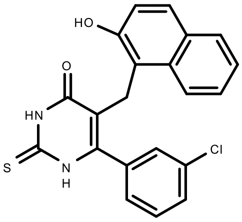

Cambinol_analogue1_6b.mol

77

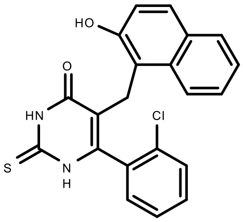

Cambinol_analogue1_6a.mol

78

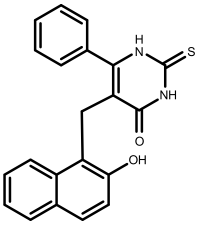

cambinol.mol

79

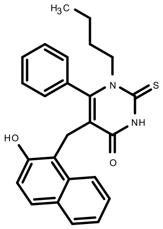

6j cambinol analog_ 6j.mol

80

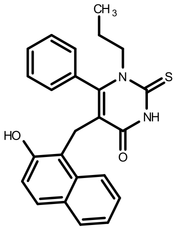

6h cambinol analog_ 6h.mol

Bin cluster size of 83

81	82	83	
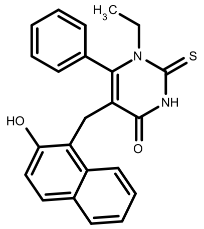	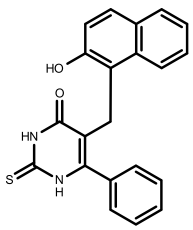	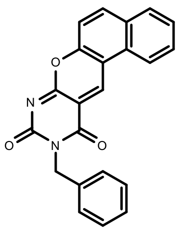	
6f cambinol analog _ 6f.mol	Benzodeazaoxaflavins_derivative1_Cambinol.mol	Benzodeazaoxaflavins_derivative1_2g.mol	
