# Supplementary figures and images for: EpiDBase: a manually curated database for small molecule modulators of epigenetic landscape
Source: Database (Oxford). 2015 Mar 16;2015:bav013. doi: 10.1093/database/bav013 (PMC4360624; doi:10.1093/database/bav013)

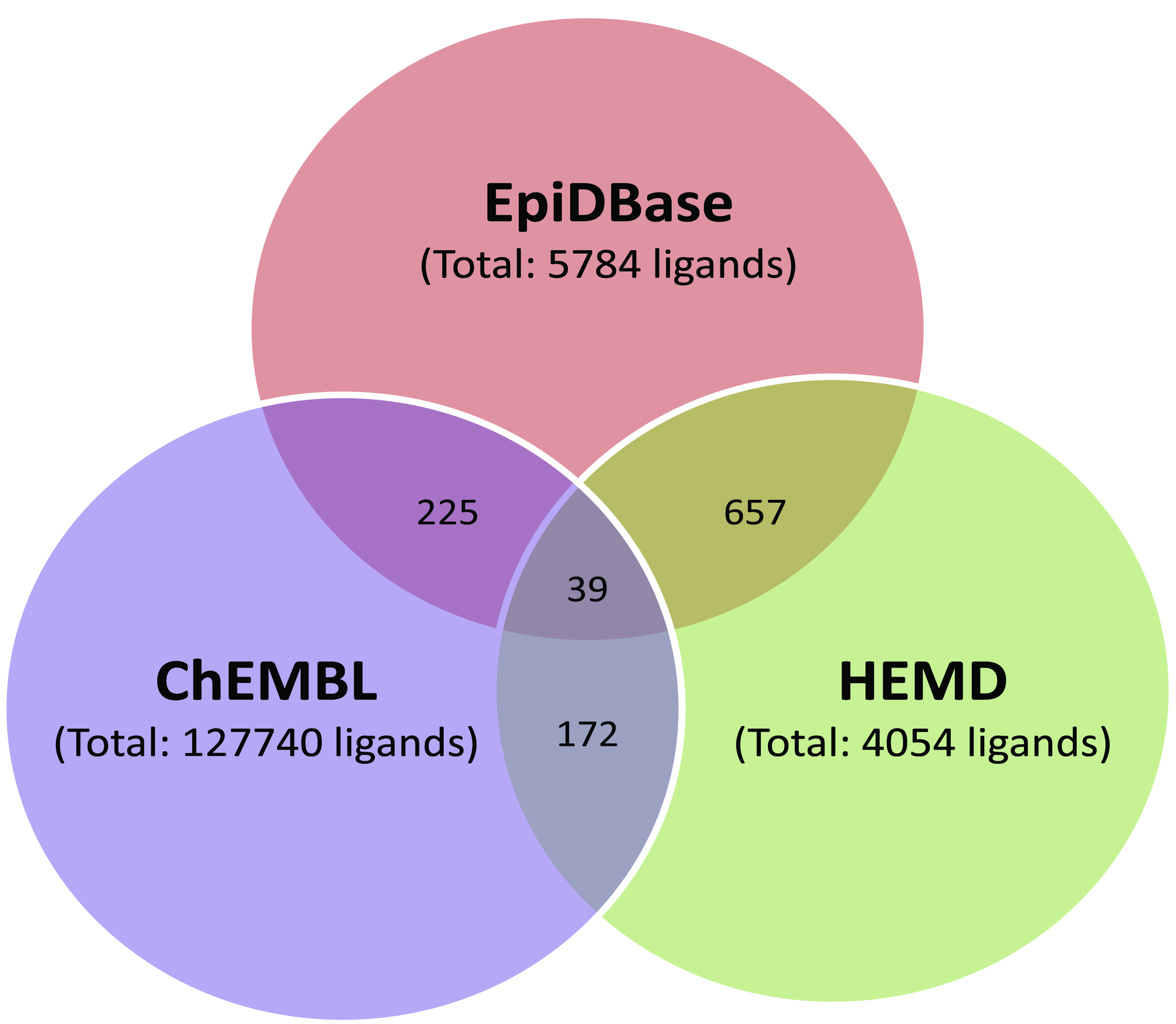

Supplement: Supplementary Data [file supp_bav013_suppl_data.zip › File_S5.tif]

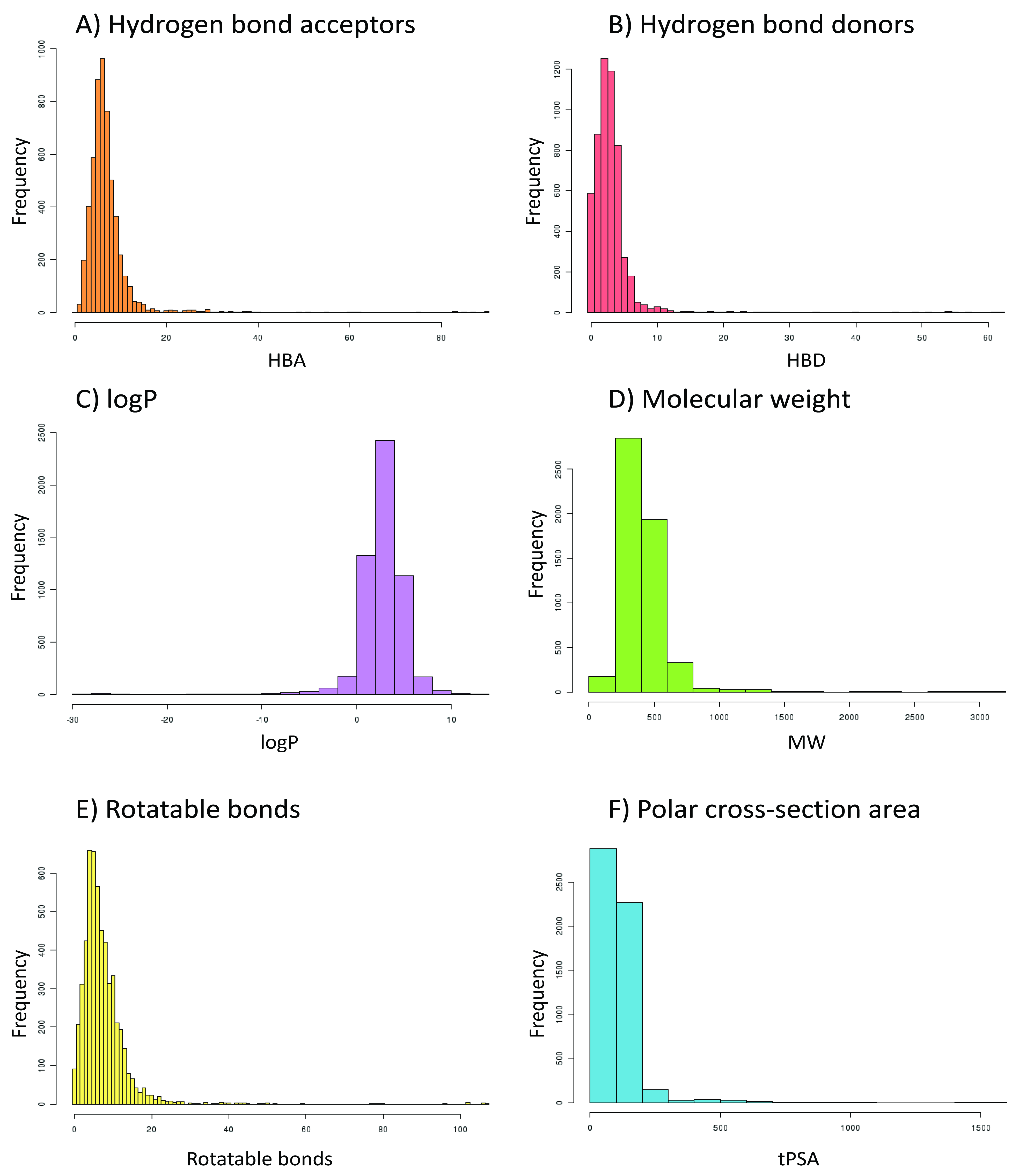

Supplement: Supplementary Data [file supp_bav013_suppl_data.zip › File_S6.tif]

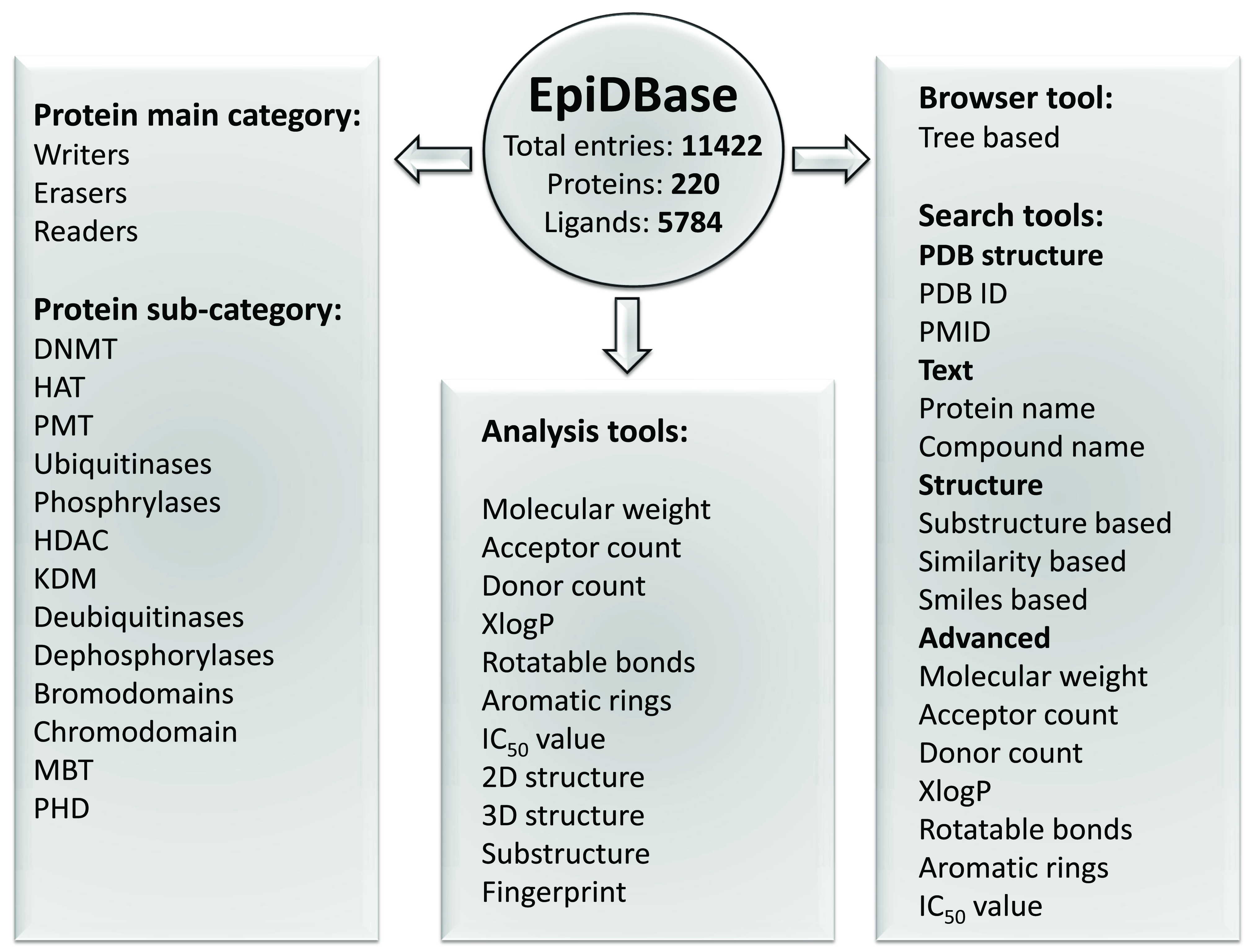

Supplement: Supplementary Data [file supp_bav013_suppl_data.zip › File_S2.tif]
