# Supplementary material for: EpiDBase: a manually curated database for small molecule modulators of epigenetic landscape
Source: Database (Oxford). 2015 Mar 16;2015:bav013. doi: 10.1093/database/bav013 (PMC4360624; doi:10.1093/database/bav013)
Supplement: Supplementary Data [file supp_bav013_suppl_data.zip › File_S1.doc]

**File S1: EpiDBase Description**

**Database description**

MySQL RDBMS (relational database management system) is used for storing data.

The main tables are as follows:

1. Category (stores information about Protein categories)

2. Compound (stores information about Ligand)

3. ProteinMainCategory

4. ProteinSubCategory

5. Protein_compound

The following notations are used in the database description:

.PRI : primary key, generally auto-generated by the database

UNI: this field will take only unique values

NULL: used to represent no value

varchar: MySQL datatype, used for storing a string of limited length efficiently

blob: MySQL datatype, used to store arbitrary binary data

int: MySQL datatype, used to represent an integer

double: MySQL datatype, used to represent a floating point number

**Table 1. Category**

| **Field** | **Type** | **Null** | **Key** | **Default** | **Extra** |
| --- | --- | --- | --- | --- | --- |
| categoryId | int(11) | NO | PRI | NULL | auto_increment |
| categoryName | text | NO |  | NULL | Protein categories(leaf node) |
| categoryDescription | text | YES | UNI | NULL |  |
| subCategoryId | int(11) | NO | MUL | NULL |  |
| proteinImage | longblob | YES |  | NULL |  |
| pdbid | text | YES |  | NULL | Proteni data bank id |
| height | Double | YES |  |  | Heigh of protein img |
| width | Double | YES |  |  | Width of protein img |

**T**

**Table 2. Compound entry**

| **Field** | **Type** | **Null** | **Key** | **Default** | **Extra** |
| --- | --- | --- | --- | --- | --- |
| compoundId | int(11) | NO | PRI | NULL | auto_increment |
| compoundName | text | NO |  | NULL | Ligand Name |
| Image | longblob | YES | UNI | NULL |  |
| moldata | longblob | YES |  | NULL | Structure file (SDF format) |
| molwt | double | YES |  | NULL | Molecular weight of ligand |
| acount | int(11) | YES |  | NULL | Acceptor count for ligand |
| dcount | int(11) | YES |  | NULL | Donor count of ligand |
| xlogp | double | YES |  | NULL | Xlogp value for Ligand |
| rbond | int(11) | YES |  | NULL | Number of rotatable bonds for Ligand |
| aromaticrings | int(11) | YES |  | NULL | Number of aromatic rings for ligand |
| structure3d | longblob | YES |  | NULL |  |
| filetype | text | YES |  | NULL |  |
| categoryId | int(11) | NO | MUL | NULL |  |
| thumbnail | longblob | YES |  | NULL |  |
| width | int(11) | YES |  | NULL |  |
| height | int(11) | YES |  | NULL |  |

**Table 3. Protein Main Category**

| **Field** | **Type** | **Null** | **Key** | **Default** | **Extra** |
| --- | --- | --- | --- | --- | --- |
| mainCategoryId | int(11) | NO | PRI | NULL | auto_increment |
| mainCategoryName | text | NO |  | NULL | (writer,reader,eraser) |

**Table 4. Protein SubCategory**

| **Field** | **Type** | **Null** | **Key** | **Default** | **Extra** |
| --- | --- | --- | --- | --- | --- |
| subCategoryId | int(11) | NO | PRI | NULL | auto_increment |
| subCategoryName | text | NO |  | NULL | HDAC,LDM etc |
| subCategoryDescription | text | YES |  | NULL |  |
| mainCategoryId | int(11) | NO | MUL | NULL |  |

**Table 5. Protein compound**

| **Field** | **Type** | **Null** | **Key** | **Default** | **Extra** |
| --- | --- | --- | --- | --- | --- |
| id | int(11) | NO | PRI | NULL | auto_increment |
| categoryId | int(11) | NO | MUL | NULL | Category id |
| compoundId | int(11) | NO | MUL | NULL | Ligand id |
| kd | text | YES |  | NULL | Kd value for the ligand- RNA association |
| ic | text | YES |  | NULL | IC50 value for the ligand- RNA association |
| pmid | text | YES |  | NULL | PubMed Id |
| comments | text | YES |  | NULL |  |
| references | text | YES |  | NULL |  |
