# Supplementary material for: EpiDBase: a manually curated database for small molecule modulators of epigenetic landscape
Source: Database (Oxford). 2015 Mar 16;2015:bav013. doi: 10.1093/database/bav013 (PMC4360624; doi:10.1093/database/bav013)
Supplement: Supplementary Data [file supp_bav013_suppl_data.zip › File_S3.doc]

**S3:ZINC parameters used for description of Physicochemical descriptors**

**Table1**. ZINC property Filter

| **Property** | **Value** |
| --- | --- |
| [MW](http://fafdrugs2.mti.univ-paris-diderot.fr/descriptors.html#MW) | 60 - 600 |
| [logP](http://fafdrugs2.mti.univ-paris-diderot.fr/descriptors.html#logP) | -4 to 6 |
| [HBA](http://fafdrugs2.mti.univ-paris-diderot.fr/descriptors.html#MW) | ≤ 11 |
| [HBD](http://fafdrugs2.mti.univ-paris-diderot.fr/descriptors.html#logP) | ≤ 6 |
| [tPSA](http://fafdrugs2.mti.univ-paris-diderot.fr/descriptors.html#tPSA) | ≤ 150 |
| [Rotatable Bonds](http://fafdrugs2.mti.univ-paris-diderot.fr/descriptors.html#RotatableBonds) | ≤ 12 |
| [Rigid Bonds](http://fafdrugs2.mti.univ-paris-diderot.fr/descriptors.html#RigidBonds) | ≤ 50 |
| [Rings](http://fafdrugs2.mti.univ-paris-diderot.fr/descriptors.html#n_SystemRing) | ≤ 7 |
| [MaxSizeSystemRing](http://fafdrugs2.mti.univ-paris-diderot.fr/descriptors.html#MaxSizeSystemRing) | ≤ 12 |
| [Carbons](http://fafdrugs2.mti.univ-paris-diderot.fr/descriptors.html#n_carbon) | ≥ 3 |
| [HeteroAtoms](http://fafdrugs2.mti.univ-paris-diderot.fr/descriptors.html#n_hetero) | ≥ 0 |
| [H/C Ratio](http://fafdrugs2.mti.univ-paris-diderot.fr/descriptors.html#ratioH_C) | ≤ 2.0 |
| [Charges](http://fafdrugs2.mti.univ-paris-diderot.fr/descriptors.html#NumCharges) | ≤ 3 |
| [TotalCharge](http://fafdrugs2.mti.univ-paris-diderot.fr/descriptors.html#TotalCharge) | -2 to 2 |
